# Development of a Highly Potent Transthyretin Amyloidogenesis Inhibitor: Design, Synthesis, and Evaluation

**DOI:** 10.1021/acs.jmedchem.2c01195

**Published:** 2022-10-28

**Authors:** Francisca Pinheiro, Irantzu Pallarès, Francesca Peccati, Adrià Sánchez-Morales, Nathalia Varejão, Filipa Bezerra, David Ortega-Alarcon, Danilo Gonzalez, Marcelo Osorio, Susanna Navarro, Adrián Velázquez-Campoy, Maria Rosário Almeida, David Reverter, Félix Busqué, Ramon Alibés, Mariona Sodupe, Salvador Ventura

**Affiliations:** † Institut de Biotecnologia i Biomedicina and Departament de Bioquímica i Biologia Molecular, Universitat Autònoma de Barcelona, Bellaterra, Barcelona 08193, Spain; ‡ Departament de Química, Universitat Autònoma de Barcelona, Bellaterra, Barcelona 08193, Spain; § Molecular Neurobiology Group, i3S−Instituto de Investigação e Inovação em Saúde, IBMC−Instituto de Biologia Molecular e Celular, Universidade do Porto, 4200-135 Porto, Portugal; ∥ Departamento de Biologia Molecular, ICBAS−Instituto de Ciências Biomédicas Abel Salazar, Universidade do Porto, 4050-313 Porto, Portugal; ⊥ Department of Biochemistry and Molecular & Cellular Biology, and Institute for Biocomputation eand Physics of Complex Systems (BIFI), Joint Unit GBsC-CSIC-BIFI, Universidad de Zaragoza, 50018 Zaragoza, Spain; # Aragon Institute for Health Research, 50009 Zaragoza, Spain; ∇ Biomedical Research Network Center in Hepatic and Digestive Diseases (CIBERehd), 28029 Madrid, Spain; ¶ ICREA, Passeig Lluis Companys 23, E-08010 Barcelona, Spain

## Abstract

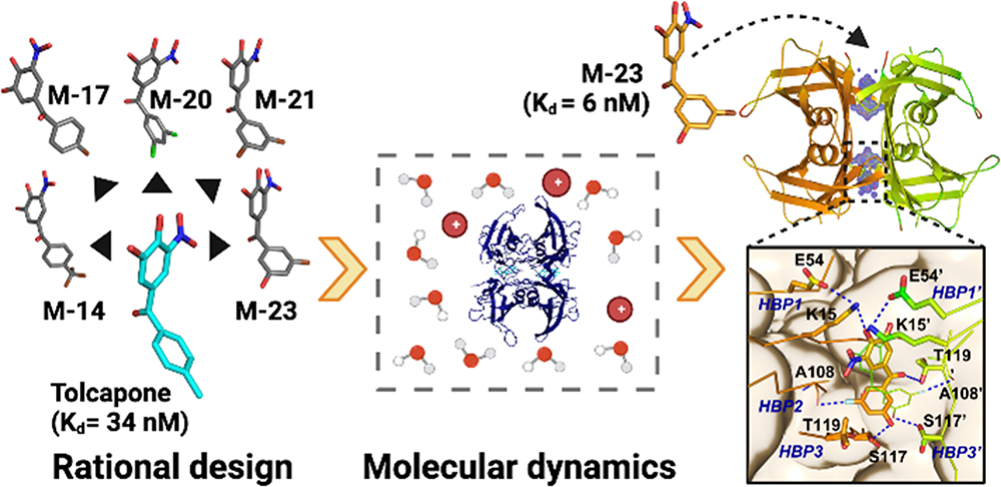

Transthyretin amyloidosis (ATTR) is a group of fatal diseases described by the misfolding and amyloid deposition of transthyretin (TTR). Discovering small molecules that bind and stabilize the TTR tetramer, preventing its dissociation and subsequent aggregation, is a therapeutic strategy for these pathologies. Departing from the crystal structure of TTR in complex with tolcapone, a potent binder in clinical trials for ATTR, we combined rational design and molecular dynamics (MD) simulations to generate a series of novel halogenated kinetic stabilizers. Among them, **M-23** displays one of the highest affinities for TTR described so far. The TTR/**M-23** crystal structure confirmed the formation of unprecedented protein–ligand contacts, as predicted by MD simulations, leading to an enhanced tetramer stability both *in vitro* and in whole serum. We demonstrate that MD-assisted design of TTR ligands constitutes a new avenue for discovering molecules that, like **M-23**, hold the potential to become highly potent drugs to treat ATTR.

## Introduction

Amyloid diseases constitute a diverse group of pathologies characterized by protein misfolding, aggregation, and the buildup of insoluble fibrils in tissues and organs throughout the body.^1^ Transthyretin (TTR) is one of many proteins related with these disorders.^2^

The liver and the choroid plexus are the major sites of TTR synthesis. TTR transports the retinol-binding protein–retinol complex and functions as a backup carrier for thyroxine (T_4_) in the blood and as a main transporter of T_4_ in the cerebrospinal fluid.^3−5^ The extracellular misfolding of TTR and subsequent accumulation of amyloid fibrils in a variety of tissues underlie the onset of a group of disorders known as transthyretin amyloidosis (ATTR).^6^

Native TTR is a homotetramer comprising four β-sheet rich subunits of 127 amino acid residues each, termed A, B, C, and D. The monomers associate via their edge β-strands, yielding two dimers (AB and CD) that further associate back-to-back to render the tetramer. The AB/CD dimer–dimer interface defines two identical funnel-shaped T_4_-binding sites at opposite sides of the molecule.^7,8^ TTR-tetramer dissociation at the T_4_-binding interface creates dimers that promptly dissociate into aggregation-prone monomers, representing the rate-limiting step during TTR misfolding and amyloid formation.^9,10^

Pathogenic mutations accelerate TTR aggregation by thermodynamic or kinetic destabilization of the protein.^11,12^ To date, more than 130 mutations in the TTR gene have been described, which result in autosomal dominant familial forms of the disease.^13^ The majority of disease-associated variants are caused by missense mutations and display tissue specificity and pathology. Familial amyloid polyneuropathy (FAP) and familial amyloid cardiomyopathy (FAC) are the most prevalent presentations of ATTR, compromising the peripheral nervous system and the heart, respectively.^14−16^ For some rare TTR mutations, central nervous system (CNS) involvement has been reported.^17,18^

Aging is another risk factor for ATTR, and deposition of wild-type (WT) TTR, preferentially in the myocardium, causes senile systemic amyloidosis (SSA), an underdiagnosed late-onset sporadic cardiomyopathy impacting up to 10–20% of the population over 65 years old.^19,20^ Notably, SSA is the leading cause of mortality in human subjects aged over 110 years.^21^

An alternative pathway for TTR amyloid formation *in vivo* proposes that TTR aggregation is triggered by proteolytic cleavage. It is supported by the fact that the amyloid deposits formed by most TTR variants *in vivo* contain the truncated 49-127 polypeptide.^22,23^ This mechanism might be especially relevant in organs with substantial shear stress, such as the heart, where the physiological fluid flow, together with the hydrophobic forces acting on the protein, might increase its susceptibility to proteolytic cleavage.^24,25^

In the last years, new therapies have been developed for the treatment of ATTR that aim to replace liver transplantation, the standard therapy for hereditary ATTR.^26,27^ Current therapeutic approaches mainly rely on reducing amyloid formation through TTR kinetic stabilization^28^ or inhibition of TTR protein synthesis (e.g., inotersen^29^ and patisiran^30^).

The kinetic stabilizer strategy gained momentum upon the identification of a TTR disease protective substitution, T119M.^31^ The T119M mutation reduces the rate of TTR tetramer dissociation and, consequently, the amyloidogenic propensity of the native ensemble.^32^

**Figure 1:**
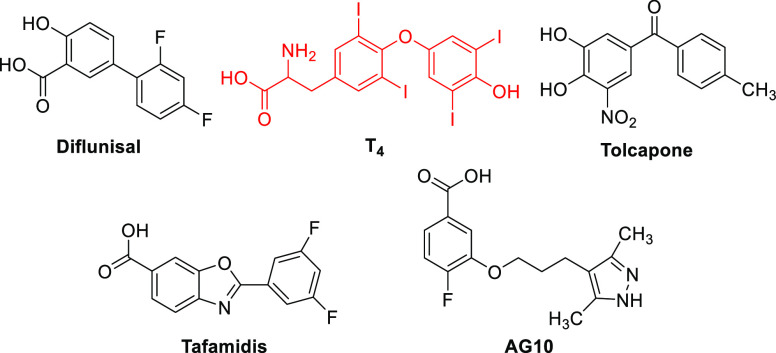
Chemical structures of T_4_^7^ (in red), the natural ligand of TTR, and examples of T_4_-inspired kinetic stabilizers.^33,37−39^

Thyroglobulin and albumin are the main transporters of T_4_ in the blood, with only 1% of TTR being bound to the hormone, and thus, T_4_ pockets are largely empty. Ligand binding at the T_4_ sites kinetically stabilizes TTR, increasing the energy barrier for tetramer dissociation. Thus, small molecules displaying selectivity and affinity for docking at T_4_ cavities have emerged as therapeutic options for treating ATTR (Figure 1). So far, only the benzoxazole tafamidis^33^ (Vyndaqel and Vyndamax) has reached the market. Treatment with tafamidis was well tolerated and has shown to delay the progression of neuropathy and cardiomyopathy in FAP^34^ and FAC,^35^ thus being approved for these indications. However, disease progression occurs in ∼30% of patients with familial ATTR,^36^ highlighting the need for developing alternative TTR kinetic stabilizers.

More recently, our group repurposed tolcapone for the treatment of ATTR. Tolcapone (Tasmar, 3,4-dihydroxy-4′-methyl-5-nitrobenzophenone) is a potent inhibitor of catechol-*O*-methyltransferase (COMT) approved in the United States and Europe as an adjunct to levodopa and carbidopa for the treatment of Parkinson’s disease. Tolcapone binds with high affinity and specificity to TTR, stabilizing the tetramer and thus preventing amyloidogenesis and protecting from TTR cytotoxicity.^39^ In a Phase IIa clinical study for FAP,^40,41^ tolcapone completely stabilized plasmatic TTR in all patients studied, and no adverse events were registered. In addition, tolcapone has been shown to inhibit TTR aggregation induced by proteolytic cleavage at physiological pH,^42^ a process that might underlie TTR amyloidogenesis *in vivo*. Noteworthily, tolcapone penetrates the blood–brain barrier^43^ and effectively inhibits the aggregation of the extremely destabilized and fast dissociating variants that cause the rare, but lethal, CNS amyloidosis.^44^

Thermodynamic analysis revealed that tolcapone binds and stabilizes TTR more effectively than tafamidis, exhibiting higher *ex vivo* anti-amyloidogenic activity. More enthalpically favorable binding to TTR, together with the lack of negative cooperativity, which in the case of tafamidis significantly reduces the affinity for the second T_4_ site, seems to underlie the higher potency of tolcapone.

Tolcapone was the best performer of our repurposed compound library. However, this does not mean that the contacts it established with the TTR T_4_-binding cavities were optimal. In a way, a new repurposed drug may be considered as a hit to be further optimized to increase its target potency and selectivity, especially if atomic structural information of the protein–drug complex is available. Here, we exploited this concept with the purpose of generating a TTR kinetic stabilizer with improved binding affinity and anti-amyloidogenic activity relative to tolcapone.

**Figure 2:**
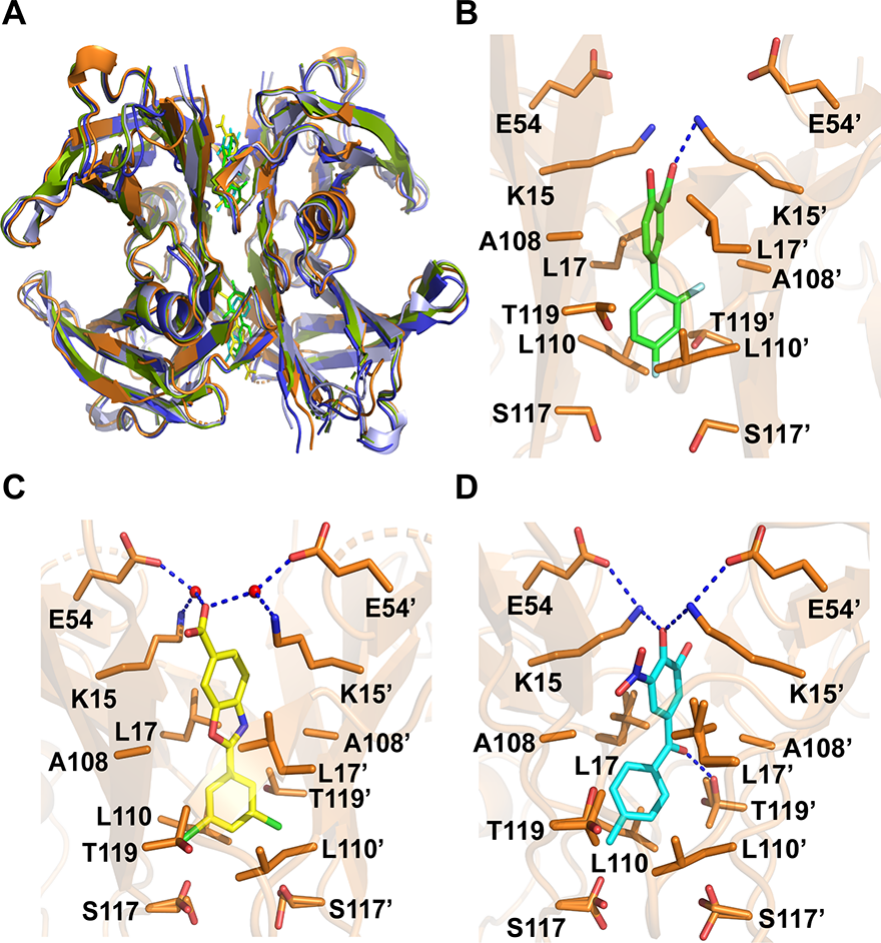
(A) Superposition of WT-TTR in Apo-form (light blue) and complexed to diflunisal (olive), tolcapone (blue), and tafamidis (orange). Overlaid tetramers are shown in cartoon, and diflunisal (green), tafamidis (yellow), and tolcapone (cyan) are represented as sticks. (B–D) Close-up view of the binding of diflunisal, tolcapone, and tafamidis to WT-TTR. Compounds are colored as in panel A. Residues interacting with TTR are shown as sticks (orange). Blue dashed lines signal the hydrogen bonds and the salt bridges. Structures prepared from PDB structures (B) 3D2T,^37^ (C) 3TCT,^33^ and (D) 4D7B.^39^

Up to now, more than 300 TTR crystal structures are available in the Protein Data Bank,^45,46^ most of them complexed to small-molecule ligands. A detailed structural study of a set of 23 high-resolution TTR structures comprising WT, non-amyloidogenic, and amyloidogenic variants concluded that they are almost exactly superimposable. Moreover, differences in the positioning of certain loops or in the side chain rotamers of some residues, including those of the T_4_-binding sites, were not significant.^47^ Accordingly, the binding of kinetic stabilizers does not result in significant TTR structural rearrangements, and indeed, the structures of TTR with or without ligands are essentially identical except for a reduced number of side chain rotamers in the vicinity of the binders (Figure 2A). This indicates that crystallographic structures correspond to static pictures where it is difficult to discern the dynamic impact of both mutations and ligands on the native TTR stability, challenging the use of structure–activity relationships to evolve stronger TTR binders.

Molecular dynamics (MD) simulations allow studying the structural dynamics of biological systems at atomic resolution.^48^ MD simulations are especially useful in modeling and assessing the binding capacity of small molecules to target proteins since they not only provide atomic information on the interaction but also allow estimation of the binding energetics.^49,50^ MD simulations have been used to investigate the impact of mutations on TTR conformational flexibility^51,52^ and to investigate the mechanism of TTR protection by existent kinetic stabilizers.^53−55^ However, to the best of our knowledge, MD-based methods for estimating binding affinities have not been employed to assist in the design of novel molecules aimed to interact with T_4_-binding sites.

Here we combined rational design and MD simulations to generate a series of tolcapone-inspired kinetic stabilizers. The candidates were chemically synthesized and experimentally validated. As a result, we describe **M-23**, a noncooperative kinetic stabilizer that binds TTR with an affinity >5-fold than tolcapone. Thermodynamic analysis indicates that this high affinity results from an optimized enthalpy of binding, whereas the TTR/**M-23** crystal structure demonstrates the effective existence of new contacts between the two moieties, as predicted by MD simulations. These interactions result in a significantly higher stabilization of the TTR tetramer *in vitro* and *ex vivo* relative to tolcapone, turning **M-23** into a promising candidate for therapeutic intervention in ATTR.

## Results and Discussion

### Rationale for the Design of Tolcapone Analogues

Each TTR T_4_-binding site contains three pairs of symmetric depressions known as halogen-binding pockets (HBPs: HBP1 and HBP1′, HBP2 and HBP2′, and HBP3 and HBP3′), wherein the four iodine atoms of the hormone reside. The innermost pocket is HBP3 and is established by Ser117, Leu110, Thr119, and Ala108 side chains. HBP1 is placed at the entrance of the T_4_-binding site and comprises Lys15, Leu17, Thr106, and Val121, whereas the central pocket HBP2 is formed by Leu17, Ala108, Ala109, and Leu110 along with the methylene carbons of Lys15.

Typically, TTR kinetic stabilizers have two aromatic rings, one ring substituted with halogens, placed at HBP2/HBP3, and the other displaying hydrophilic substituents, placed at HBP1. The nonsteroidal anti-inflammatory drug diflunisal (Figure 2B), already in clinical trials,^56^ exemplifies these properties, with the difluorophenyl group pointing to the inner part of the channel and the two fluor atoms located in HBP2. In HBP1, the carboxy group of diflunisal establishes a salt bridge with the amino group of Lys15.

In tafamidis (Figure 2C), the 3,5-dichloro moiety is surrounded by the residues in the HBPs 3/3′, whereas the carboxy end forms a water-mediated hydrogen bond with the amino group of Lys15. The highest binding affinity of tafamidis, in respect to diflunisal, is difficult to explain from the crystal structure in terms of specific protein–compound interactions and has been attributed to a stronger halogen bonding capability of the chloride moiety.

In tolcapone (Figure 2D), the 4-methyl-phenyl ring occupies HBP3, and a specific hydrogen bond is established between the linker carbonyl group of the compound and the hydroxyl side chain of Thr119. The 3,4-dihydroxy-5-nitrophenyl ring of tolcapone is positioned in HBP1, forming a hydrogen bond with Lys15, which in turn stabilizes the ionic interactions between Lys15 and Glu54. These direct interactions in the outer face of the cavity, together with hydrogen bonding to Thr119, likely contribute to the favorable enthalpic binding of this molecule, explaining why it stabilizes TTR more effectively than tafamidis.

In contrast to diflunisal and tafamidis, tolcapone does not possess halogen atoms, and although the methyl group is placed in a favorable environment in HBP3, it cannot establish the halogen bonds that characterize T_4_, diflunisal, and tafamidis. Halogenation has been shown to be effective at increasing the affinity of certain TTR ligands, like in the case of iododiflunisal.^57^ Therefore, we decided to use MD simulations to study if endorsing tolcapone with different halogen moieties, while trying to keep the optimal hydrogen bonding capability of the upper ring and the middle carbonyl group intact, might result in optimized kinetic stabilizers.

### Molecular Dynamics Simulations of Halogenated Tolcapone Analogues

**Figure 3:**
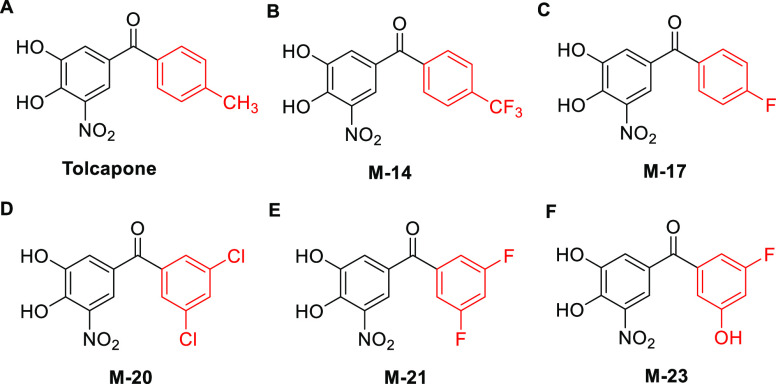
(A–F) Chemical structures of tolcapone derivatives synthesized and evaluated in this study . The second aryl ring is shown in red to highlight the differences between the compounds.

All the new ligands developed in the present work are benzophenone derivatives with two phenyl moieties, one common to all ligands, i.e., the 3,4-dihydroxy-5-nitrophenyl ring, more exposed to the solvent, and a second one including different halogen substituents pointing toward the inner binding pocket (Figure 3). Differences are thus expected to arise from the distinct contacts between this inner aryl moiety and TTR at the binding site.

In the first two ligands, the 4-CH_3_ group of tolcapone was substituted by either a −CF_3_ group or −F, rendering compounds **M-14** and **M-17**, respectively (Figure 3A–C). The computed binding energies of tolcapone, **M-14**, and **M-17** to human WT-TTR and the main ligand–TTR hydrogen bond contacts (>10% frequency along the trajectory) are given in Table 1. Simulations are expected to provide the proper trends, although not absolute values. Note that for absolute affinities, the entropy term, particularly that owing to the decrease of translational and rotational freedom when the ligand binds to the protein, should be included. However, this entropic term should not vary significantly between our candidate molecules,^58^ and as such, it is not expected to affect ligand comparisons.

**Table 1:** Ligand-TTR Binding Energies (Δ*G*_bind_), Gas Phase Binding Energies (Δ*E*_gp_), Ligand Solvation Energies (Δ*G*_L-solv_), in kcal mol^–1^, and Main Ligand–TTR H-Bond Contacts

compound	Δ*G*_bind_^a^	Δ*E*_gp_^b^	Δ*G*_L-solv_	ligand···TTR contacts
tolcapone	59.7	81.5	–10.9	C=O···T119
**M-14**	55.4	76.6	–10.6	
**M-17**	68.0	90.2	–11.1	C=O···T119, F···S117, F···T119

a Δ*G*_bind_ = Δ*E*_gp_ + 2 × Δ*G*_L-solv_.

b Δ*E*_gp_ = *E*_TTR_ + 2*E*_L_ – *E*_TTR-2L_.

As expected, all starting structures, derived from the crystal structure of the TTR–tolcapone complex (PDB: 4D7B), with the two ligands related by a C_2_ symmetry axis, lose their initial symmetry along the simulation; i.e., the interactions between each ligand with AB or CD, albeit similar, are not identical. Furthermore, in all cases, the specific interactions in the outer binding pocket between the hydroxyl groups of the 3,4-dihydroxy-5-nitrophenyl moiety and Lys15 or between Glu54 and Lys15 identified in the crystal structure are only maintained in less than 5% of the trajectory due to temperature effects. Thus, from now on, we will focus on the interaction between the central C=O of the ligand and the hydroxyl group of Thr119 and on the additional ligand–TTR specific interactions resulting from the new substitutions on the phenyl moiety. The main structures, together with the H-bond evolution along the trajectories, are provided in Figure S1. Specific ligand–TTR contacts, with their frequency and shortest and average distances, are given in Table S1.

Regarding tolcapone, the C=O···Thr119 interaction appears with a frequency of 10%, with the minimum distance value being 2.63 Å, which resembles the one observed in the crystal structure (2.55 Å). For **M-14**, this interaction is lost, appearing in less than 1% of the trajectory. This is due to the presence of the −CF_3_ bulkier substituent, which introduces larger repulsive interactions that hinder the entrance into the cavity and leave the ligand more exposed to the solvent. Indeed, the shortest distance between the two ligands in these two complexes, taken as the distance between the C atoms of the two ligands’ C=O groups, is significantly larger in **M-14** (19.9 Å) than in tolcapone (15.8 Å). Consequently, **M-14** exhibits a smaller calculated binding energy (55.4 kcal mol^–1^) than tolcapone (59.7 kcal mol^–1^). In contrast, for **M-17**, with a −F in the para position instead of a −CF_3_ group, the C=O···Thr119 interaction is maintained with an average frequency of ∼50%, with the shortest C=O···HO_T119_ distance being 2.55 Å. In addition, new contacts between the −F substituents and both Thr119 and Ser117 appear, which further enhance the calculated ligand–TTR binding energy (68.0 kcal mol^–1^) as compared to tolcapone (59.7 kcal mol^–1^).

### Binding of Halogenated Tolcapone Analogues to TTR

**Scheme 1:**
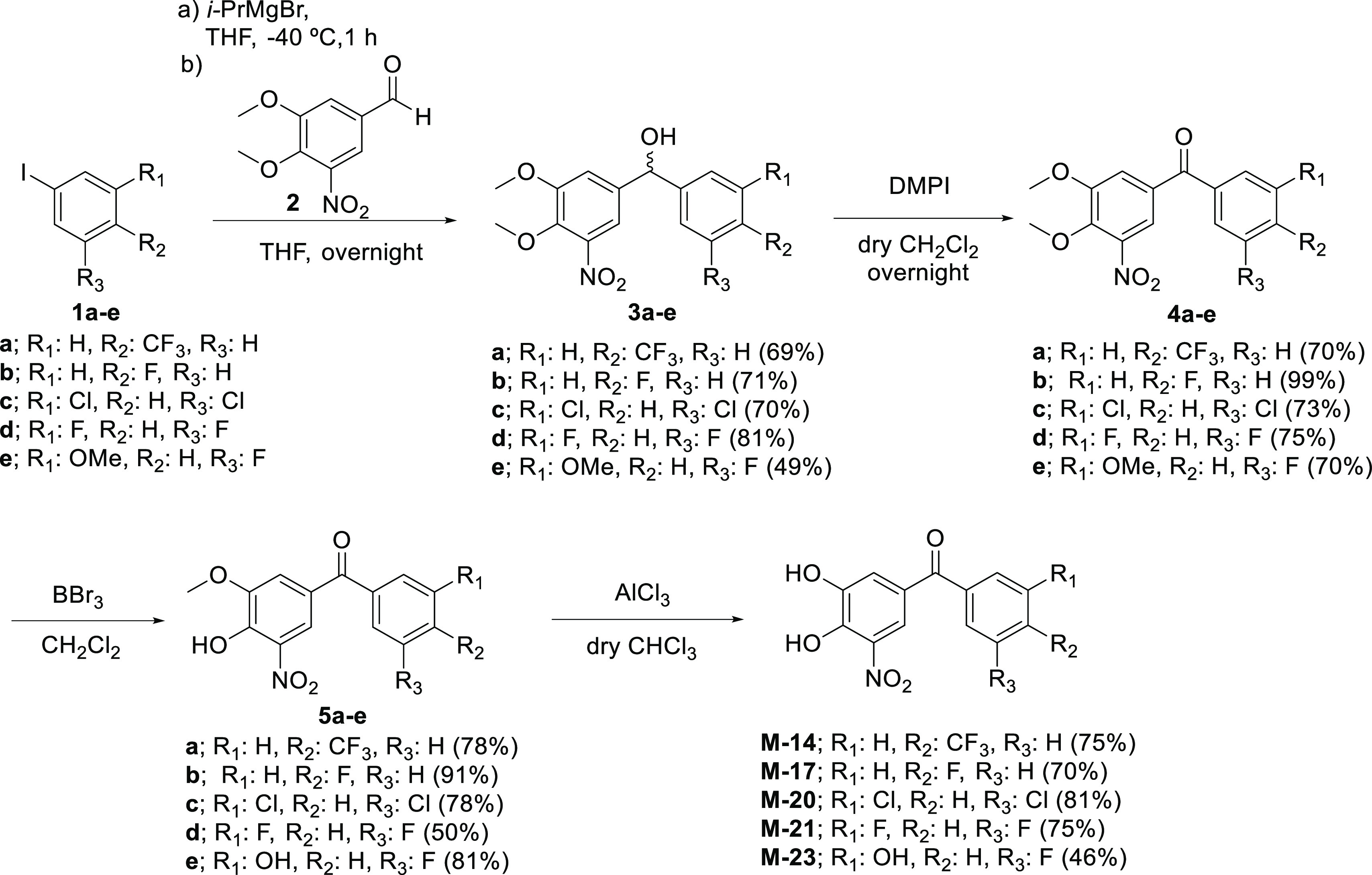
Synthesis of Tolcapone Analogues

**M-14** and **M-17** were chemically synthesized as shown in Scheme 1. The synthesis procedure is explained in detail in the Experimental Section.

**Figure 4:**
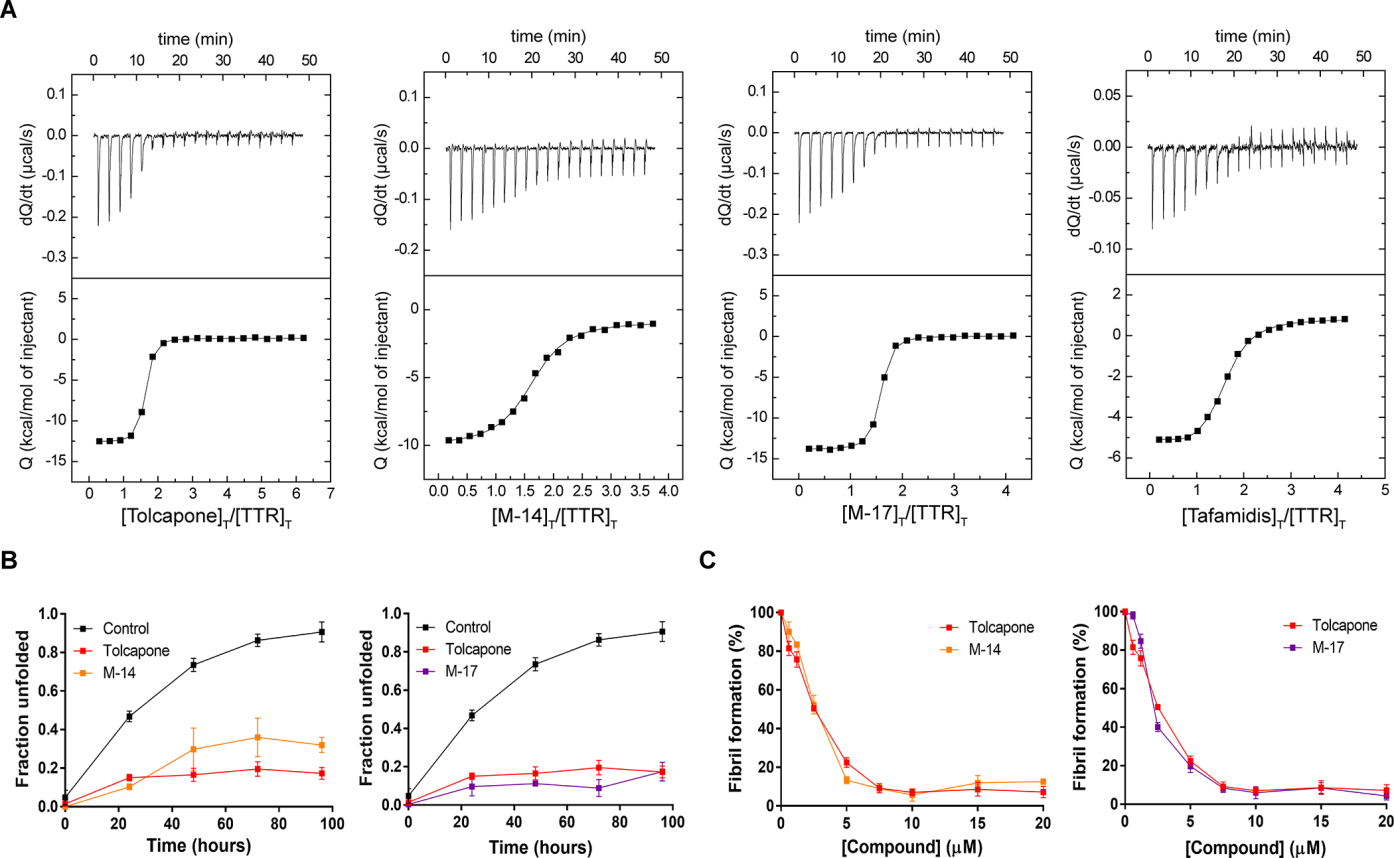
*In vitro* characterization of halogenated tolcapone analogues, **M-14**, and **M-17**. (A) Interaction of WT-TTR with tolcapone, **M-14**, and **M-17** as assessed by ITC. Tafamidis was tested as a reference. The top panels represent the raw data (thermogram), while the lower panels correspond to the integrated heat changes upon binding plotted against the ligand/TTR concentration ratio (binding isotherm). The solid line describes the best fit according to a two-site binding model (with or without cooperativity) for each test compound. (B) WT-TTR (1.8 μM) urea-induced tetramer dissociation (6 M urea) in the absence or presence of tolcapone, **M-14**, or **M-17** (at 3.6 μM), as measured by Trp fluorescence. The values correspond to mean ± SEM (*n* = 3). (C) Acid-mediated TTR (at a final assay concentration of 3.6 μM) aggregation as a function of inhibitor concentration determined by turbidity at 340 nm. The values refer to mean ± SEM (*n* = 3).

Isothermal titration calorimetry (ITC) was used to characterize their binding affinities for WT-TTR, and the thermodynamic parameters that describe the reaction were determined. Ligand binding to TTR can be cooperative (positive or negative cooperativity) or noncooperative.^59−61^ Non- or positive cooperativity is desired; however, most reported ligands exhibit negative cooperativity,^33,62,63^ which implies a loss of affinity for the second binding site after binding to the first one. Tolcapone, **M-14**, and **M-17** bound to TTR without any cooperativity (Figure 4A and Table 2), whereas tafamidis, included as a reference, exhibited the typical negative cooperative behavior (Kd_1_ = 9.9 nM and Kd_2_ = 260 nM).

**Table 2:** Thermodynamic Parameters Determined by ITC for the Binding of **M-14** and **M-17** to WT-TTR

	*K*_d_ (nM)	Δ*G* (kcal mol^–1^)	Δ*H* (kcal mol^–1^)	–*T*Δ*S* (kcal mol^–1^)
tolcapone	34	–10.2	–12.8	2.6
tafamidis	9.9^a^	–10.9^a^	–6.0^a^	–4.9^a^
	260^b^	–9.0^b^	–6.5^b^	–2.5^b^
**M-14**	310	–8.9	–9.2	0.3
**M-17**	31	–10.3	–14.0	3.7

a Correspond to the values for the first binding site of TTR.

b Correspond to the values for the second binding site of TTR.

In agreement with the trend observed in the MD binding energy calculations, **M-14** exhibited a higher *K*_d_ (310 nM) and a lower enthalpic contribution to binding (Δ*H* = – 9.2 kcal mol^–1^) than tolcapone (*K*_d_ = 34 nM and Δ*H* = −12.8 kcal mol^–1^), whereas the affinity and enthalpic binding term of **M-17** were higher (*K*_d_ = 31 nM and Δ*H* = −14.0 kcal mol^–1^). The strong binding of tolcapone and **M-17**, with dissociation constants in the low nanomolar range, was entirely enthalpically driven (Δ*H* < 0; −*T*Δ*S* > 0). As observed in MD simulations, this indicates the formation of specific noncovalent interactions between the protein and the ligand. Enthalpy–entropy compensation effects resulted in very similar Δ*G* values for **M-17** and tolcapone.

### TTR Kinetic Stabilization by Halogenated Tolcapone Analogues

We addressed whether **M-14** and **M-17** kinetically stabilize TTR, inhibiting urea-induced tetramer dissociation. Using urea concentrations in the post-transition region for tertiary structural changes allows measuring tetramer dissociation, as the monomers unfold in a few milliseconds and remain unfolded.^11,64^ Accordingly, TTR samples were incubated in the absence or presence of compounds, and TTR denaturation was triggered by adding 6 M urea. Tertiary structural changes were monitored along time by tryptophan (Trp) intrinsic fluorescence, which was used to determine the fraction of unfolded protein at any time (Figure 4B). Tolcapone was analyzed in parallel for comparative purposes.

The three molecules significantly decreased the amount of dissociated tetramer, as well as the rate of tetramer dissociation, when present at equimolar levels relative to T_4_-binding sites. Tolcapone protected up to 82.7 ± 3.1% of TTR molecules from urea induced unfolding; **M-17** provided a similar degree of protection, whereas **M-14** was less effective.

### TTR Anti-aggregation Activity of Halogenated Tolcapone Analogues

The anti-amyloidogenic activity of **M-14** and **M-17** was evaluated using a well-established fibril-formation assay^11,65^ and compared with that of tolcapone. TTR (7.2 μM) was mixed with increasing concentrations of the compound (0–40 μM) for 30 min (pH 7.4, at 37 °C), and then, the pH was lowered to 4.2, which is the most favorable pH for TTR fibrilization.^66^ After an additional incubation of 72 h, the percentage of conversion of native TTR into amyloid fibrils was calculated by measuring turbidity at 340 nm and is reported relative to TTR incubated in the same conditions in the absence of an inhibitor (100%) in Figure 4C.

The three molecules displayed a strong anti-amyloidogenic activity, decreasing TTR aggregation in a concentration-dependent manner. In all cases, the protection was >60% at the equimolar total concentration (one molecule of the test compound bound per molecule of the TTR tetramer) and ≥87.4 when the compound concentration was greater than or equal to the one of T_4_-binding sites. These results indicate that tolcapone is already a very potent inhibitor of TTR aggregation, reaching up to 92.8% inhibition at 20 μM; accordingly, despite **M-17** being a slightly better binder and kinetic stabilizer than the original molecule, these properties do not translate into an optimization of its anti-aggregation properties, at least at acidic pH.

### Molecular Dynamics Simulations of Demethylated 3,5-Disubstituted and Halogenated Tolcapone Analogues

In **M-17**, replacing tolcapone −CH_3_ by −F resulted in increased affinity and a higher enthalpic contribution to the binding. This is in line with the suggestion that, in tafamidis, the −Cl atoms in the 3,5-dichloro moiety contribute significantly to binding in the innermost TTR HBP3 pocket. Therefore, we designed **M-20**, a chimeric molecule in which the 4-methyl-phenyl ring of tolcapone was replaced by the 3,5-dichloro-phenyl ring in tafamidis (Figure 3D). The idea was to combine the favorable noncovalent contacts of tolcapone in the outer and middle sections of the T_4_ cavity with those established by tafamidis in the inner pocket. In addition, because −Cl and −F seemed to contribute differentially to binding in HPB3,^67^ as deduced from the affinities for TTR of diflunisal and tafamidis, we designed **M-21**, in which the lower tolcapone ring was substituted by a 3,5-difluoro-phenyl ring (Figure 3E). Thus, in a way, **M-21** is a chimera of tolcapone and diflunisal, although the −F substituents lay in different relative positions.

The computed binding energies of **M-20** and **M-21** to human WT-TTR and the main ligand–TTR hydrogen bond contacts (>10% frequency along the trajectory) are provided in Table 3. The main structures for all systems, together with the H-bond evolution along the trajectories, are shown in Figure S2A,B. Specific ligand–TTR contacts, with their frequency and shortest and average distances, are given in Table S2.

**Table 3:** Ligand–TTR Binding Energies (Δ*G*_bind_), Gas Phase Binding Energies (Δ*E*_gp_), Ligand Solvation Energies (Δ*G*_L-solv_), in kcal mol^–1^, and Main Ligand–TTR H-Bond Contacts

compound	Δ*G*_bind_^a^	Δ*E*_gp_^b^	Δ*G*_L-solv_	ligand···TTR contacts
**M-20**	57.0	79.0	–11.0	Cl···T119
**M-21**	74.0	95.2	–10.6	C=O···T119, F···S117
**M-23**	84.4	116.2	–15.9	C=O···T119, OH···S117, F···S117

a Δ*G*_bind_ = Δ*E*_gp_ + 2 × Δ*G*_L-solv_.

b Δ*E*_gp_ = *E*_TTR_ + 2*E*_L_ – *E*_TTR-2L_.

For **M-21**, the C=O···Thr119 interaction appears with a frequency of 20%, with the shortest C=O···HO_T119_ distance being 2.63 Å. In **M-20**, this interaction is lost, occurring only in less than 1% of the trajectory. As in **M-14**, this seems to respond to the bulkier substituents of **M-20** (−Cl) relative to **M-21** (−F) since the distance between the C atoms of the two ligands’ C=O groups is significantly larger in **M-20** (19.4 Å) than in **M-21** (15.3 Å) or tolcapone (15.8 Å). Because of this displacement toward the upper part of the T_4_ cavity, one of the −Cl atoms contacts Thr119 in **M-20**. As in **M-17**, the −F atoms in **M-21** establish additional interactions with Ser117 in the HBP3 pocket, whereas no such contacts were observed for **M-20**. The interplay of interactions results in the calculated binding energies for **M-20** (57.0 kcal mol^–1^) and **M-21** (74.0 kcal mol^–1^) being lower and higher than the one of tolcapone (59.7 kcal mol^–1^), respectively.

The high binding energy of **M-21** indicates that the contacts between the phenyl ring substituents and the inner Ser117 make important contributions to the molecule–TTR complex. With this idea in mind, we designed **M-23** (Figure 3F), in which −F in R3 was substituted by an −OH to favor the formation of a specific hydrogen bond with the side chain of Ser117. The MD simulations indicate that this interaction is formed with a frequency that ranges from 15 to 90% (Table S2), with the shortest distance being 2.52 Å. In addition, the C=O···Thr119 contact is maintained in **M-23**, with a frequency of 37 and 49% for ligands 1 and 2, respectively, and a minimum distance of 2.59 Å. As a result, **M-23** displays the highest binding energy among the modeled compounds (84.4 kcal mol^–1^) and is expected to be a significantly better binder than tolcapone (Table 3, Figure S2C, and Table S2). Furthermore, and as found for **M-21**, a slightly larger internalization of the ligand is observed for **M-23** compared to tolcapone, with the shortest distance between the C atoms of the two ligands’ C=O groups being 15.3 Å for **M-23** and 15.8 Å for tolcapone.

Of note, in addition to the molecule–protein interactions, potential new residue–residue contacts induced by the binding of the compound might contribute to the tetramer stability. In particular, the buried Ser117 residue in each TTR subunit can establish an intersubunit hydrogen bond with Ser117 in the other monomer in the dimer (A–B or C–D contacts). MD simulations indicate that these interactions are far more frequent when TTR is bound to the ligands than when the T_4_-binding cavity is empty (Table S3). Noticeably, among the double substituted compounds, **M-23** is the one rendering the smaller average distances between the Ser117 residues in the weaker AB/CD dimer–dimer interface (Table S4).

### Binding of Demethylated 3,5-Disubstituted and Halogenated Tolcapone Analogues

**Figure 5:**
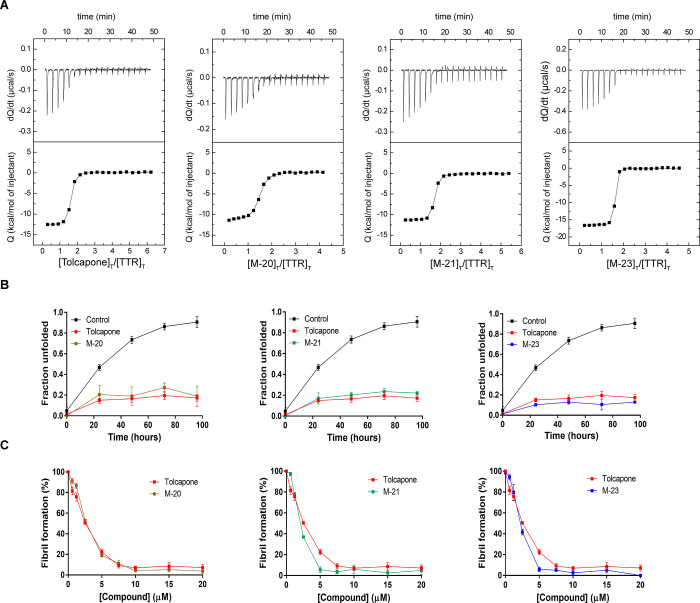
*In vitro* characterization of demethylated 3,5-disubstituted and halogenated tolcapone analogues, **M-20**, **M-21**, and **M-23**. (A) Interaction of WT-TTR with **M-20**, **M-21**, and **M-23** as assessed by ITC. The top and the lower panels correspond to the thermogram and the binding isotherm, respectively. The solid line describes the best fit according to a two-site binding model without cooperativity for each compound. (B) WT-TTR (1.8 μM) urea-induced tetramer dissociation (6 M urea) in the absence or presence of **M-20**, **M-21**, or **M-23** (at 3.6 μM), as measured by Trp fluorescence. The values correspond to mean ± SEM (*n* = 3). (C) Acid-mediated TTR (at a final assay concentration of 3.6 μM) aggregation as a function of inhibitor concentration determined by turbidity at 340 nm. The values represent mean ± SEM (*n* = 3).

Compounds **M-20**, **M-21**, and **M-23** were obtained according to Scheme 1. The chemical synthesis is detailed in the Experimental Section. ITC experiments indicated that the three molecules bound strongly to TTR without apparent cooperativity (Figure 5A and Table 4). In excellent agreement with MD simulations, **M-20** exhibited a higher *K*_d_ (85 nM) and lower enthalpic contribution to binding (Δ*H* = – 11.7 kcal mol^–1^) than tolcapone (*K*_d_ = 34 nM and Δ*H* = −12.8 kcal mol^–1^). The affinity of **M-21** was higher (*K*_d_ = 26 nM) than that of tolcapone, whereas the enthalpic binding term was lower (Δ*H* = −11.3 kcal mol^–1^). However, a lower entropic penalty for binding in **M-21** results in a slightly higher Δ*G* (−10.4 kcal mol^–1^) relative to tolcapone (−10.2 kcal mol^–1^).

**Table 4:** Thermodynamic Parameters Determined by ITC for the Binding of **M-20**, **M-21**, and **M-23** to WT-TTR

	*K*_d_ (nM)	Δ*G* (kcal mol^–1^)	Δ*H* (kcal mol^–1^)	–*T*Δ*S* (kcal mol^–1^)
tolcapone	34	–10.2	–12.8	2.6
**M-20**	85	–9.7	–11.7	2.0
**M-21**	26	–10.4	–11.3	0.9
**M-23**	6.2	–11.2	–16.6	5.4

The *K*_d_ of **M-23** is exceptionally low (6.2 nM), corresponding to a binding affinity >5-fold than that of tolcapone, with a very high enthalpy for binding (Δ*H* = −16.6 kcal mol^–1^). The binding of **M-23** is completely enthalpically driven, with an entropy penalty (5.4 kcal mol^–1^) higher than tolcapone (2.6 kcal mol^–1^), likely due to its higher polarity, but a still higher ΔG (−11.2 kcal mol^–1^). Compared with tafamidis, **M-23** displays a higher affinity for the first and especially for the second binding site where its binding is >40-fold stronger. In addition, the enthalpy contribution for binding to any of the two sites is at least 2.5-fold higher in **M-23** than in tafamidis. Overall, the thermodynamic analysis perfectly agrees with MD simulations and demonstrates a significantly optimized TTR binding in **M-23**.

### TTR Kinetic Stabilization by Demethylated 3,5-Disubstituted and Halogenated Tolcapone Analogues

Analysis of the kinetic stability induced by **M-20**, **M-21**, and **M-23** by monitoring TTR tertiary structural changes in the presence of 6 M urea indicated that all of them significantly decreased the amount of dissociated tetramer, as well as the rate of tetramer dissociation, when present at an equimolar ratio relative to T_4_-binding sites (Figure 5B). **M-20** and **M-21** performed worse and equal to tolcapone, respectively. **M-23** appears as the strongest kinetic stabilizer in the assay conditions, protecting as much as 87.0 ± 0.1% of TTR molecules from urea-induced unfolding.

### TTR Anti-aggregation Activity of Demethylated 3,5-Disubstituted and Halogenated Tolcapone Analogues

**M-20**, **M-21**, and **M-23** effectively prevented TTR amyloid formation at acidic pH (Figure 5C). They decreased TTR aggregation in a concentration-dependent mode, with >60% reduction at 1:1 TTR/compound ratio and a decrease of >90% when the compound concentration was greater than or equal to that of T_4_-binding sites. As mentioned above, tolcapone is a very potent TTR aggregation inhibitor. The weaker binder in this series (**M-20**) equals its potency, while **M-21** and **M-23** perform better, completely abolishing amyloid fibril formation when present at 20 μM.

### **M-23** Stabilizes TTR in Human Plasma

Altogether, the previous results suggested **M-23** as the most promising compound in our set and encouraged us to investigate its activity further. First, we assessed the performance of **M-23** in human plasma, a complex biological fluid where different issues, including unspecific binding to other plasma proteins, can compromise ligand efficacy.

The capacity of **M-23** to inhibit TTR tetramer dissociation in human plasma was evaluated by isoelectric focusing (IEF) electrophoresis under partially denaturing conditions (4 M urea). Tolcapone was used as a control. The assay allows one to quantify the proportion of monomer and tetramer in the sample and to calculate the extent of tetramer stabilization.

**Figure 6:**
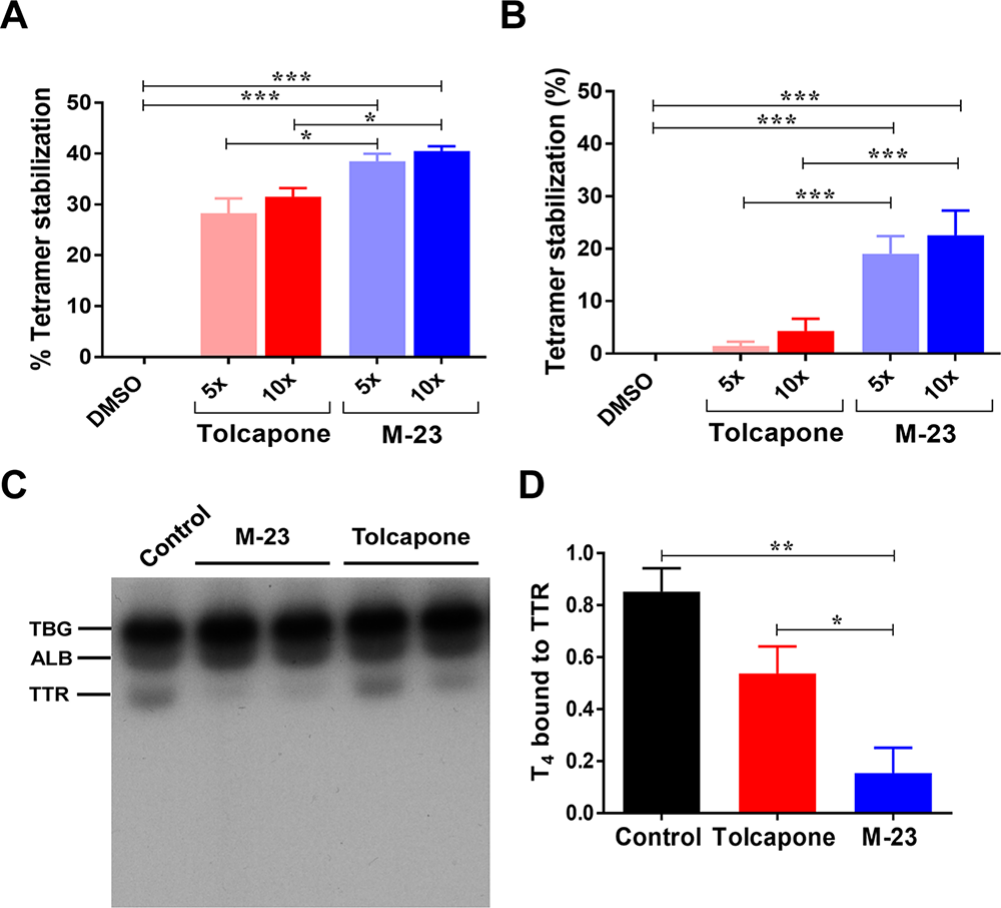
**M-23** tetramer stabilization effect assessed by IEF and its binding to TTR in human plasma. (A, B) Percentage of TTR tetramer stabilization upon incubation of **M-23** with recombinant (A) WT-TTR or (B) human plasma as evaluated by IEF under partially denaturing conditions. **M-23** was 5 or 10 times more concentrated than WT-TTR. Error bars represent SEM of mean values (*n* = 3 for recombinant protein; *n* = 6 for plasma samples); *p < 0.05; **p < 0.01; ***p < 0.001. (C) Representative native gel electrophoresis showing the distribution of [^125^I]-T_4_ after incubation with human plasma in the absence or presence of compounds. In the absence of compounds, three bands can be observed that correspond to the major plasma T_4_ binding proteins: T_4_-binding globulin (TBG), albumin (ALB), and TTR. Plasma incubated with DMSO was used as negative control. (D) Fraction of T_4_ bound to TTR in the plasma of individuals incubated with or without compounds as determined by densitometry. The values were normalized to the control, which corresponds to the maximum. The values represent mean ± SEM (*n* = 4).

First, we wanted to assess if this assay can detect differences in tetramer stabilization by **M-23** and tolcapone when using purified recombinant proteins. TTR (6 μM) was incubated in the presence or absence of 30 and 60 μM compound overnight at 4 °C. As shown in Figure 6A, **M-23** exerted a higher stabilizing effect than tolcapone at both tested concentrations. Then, the same assay was performed in human plasma (Figure 6B). **M-23** was significantly more effective than tolcapone, with a stabilizing effect >5-fold the one exerted by the original molecule. This indicates selective and tight binding of **M-23** to circulating TTR in plasma, as confirmed using the T_4_ binding competition assay. **M-23** decreased the binding of T_4_ to TTR in human plasma by 84.6 ± 9.7%, outperforming tolcapone in the same conditions (Figure 6C,D). The aqueous solubility of the two molecules was determined to rule out that differences in solubility could underlie the observed differences in binding (Table 5). **M-23** and tolcapone showed similar solubility values (0.045 mg/mL for **M-23** and 0.056 mg/mL for tolcapone), suggesting that these parameters do not influence the results obtained. Importantly, in our assays, **M-23** has a stabilizing effect in human plasma >10-fold the one exerted by tafamidis (Figure S3), the only marketed molecule for ATTR.

**Table 5:** Solubility Experiments Performed for **M-23** and Tolcapone^a^

	weight (g)	weight (mg)	water (mL)	solubility (mg/mL)
**M-23** assay 1	0.0154	15.4	350	0.044
**M-23** assay 2	0.0147	14.7	326	0.045
**M-23** assay 3	0.0150	15.0	333	0.045
**M-23** average				0.045
tolcapone assay 1	0.0150	15.0	267	0.056
tolcapone assay 2	0.0144	14.4	261	0.055
tolcapone assay 3	0.0153	15.3	273	0.056
tolcapone average				0.056

a The experiments were performed in triplicate.

The increased binding selectivity and stabilization potency of **M-23** in human plasma, relative to tolcapone, likely result from its higher enthalpy for binding, as deduced from ITC data. This is in accordance with recently reported data that suggest that the correlation between Δ*H* and the selectivity and efficacy of TTR stabilizers in human plasma is greater than with Δ*G* or *K*_d_,^68^ two parameters that in any case are also better in **M-23**.

### **M-23** Is Innocuous for Human Cells

Cytotoxicity analyses were performed for evaluating the potential M-23 chemical toxicity to human cells. Two cell lines were chosen for this purpose: HeLa, a human epithelioid cervix carcinoma cell line, and HepG2, a human hepatocellular carcinoma cell line. These cell lines are well characterized and have been widely used for *in vitro* assessment of compound toxicity.^69−71^ In particular, HepG2 is the most frequently used cell line in the testing and investigation of drug-induced liver damage,^72,73^ which is especially relevant in the context of this study, as tolcapone has been associated with cases of hepatotoxicity.^74,75^

**Figure 7:**
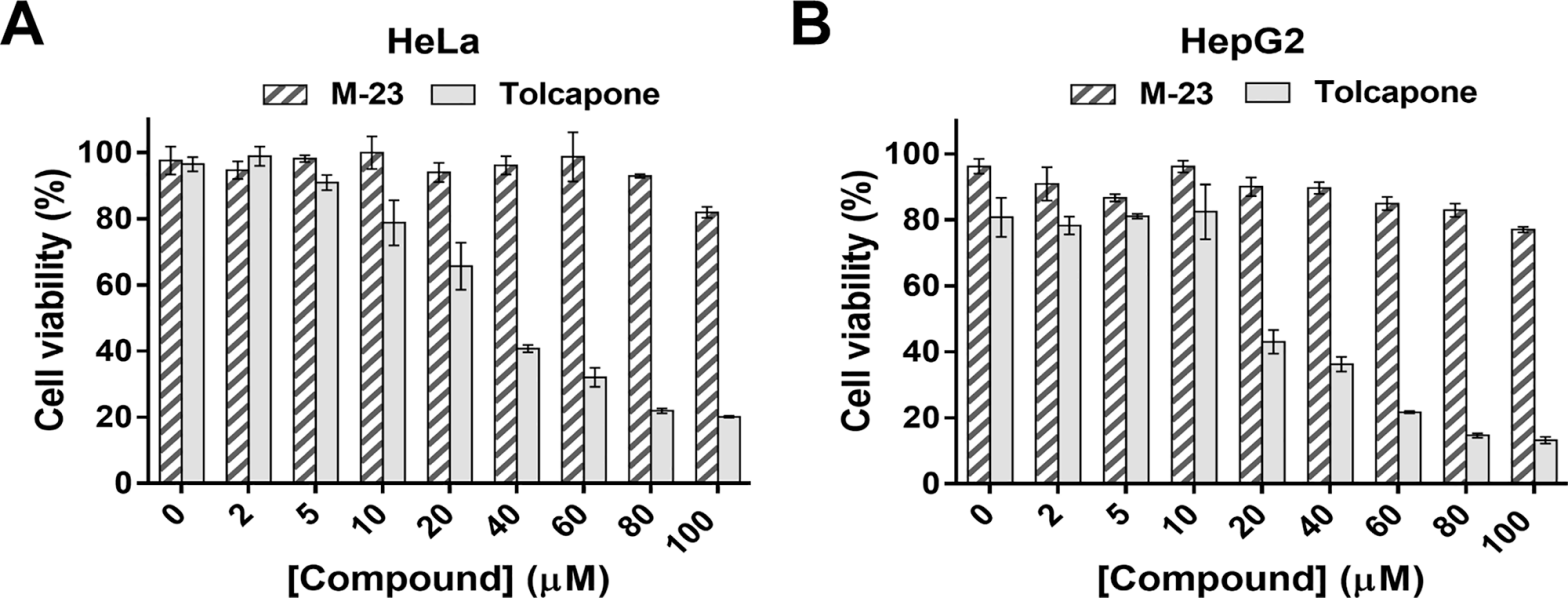
(A) HeLa and (B) HepG2 cell viability of cells exposed to increasing concentrations of **M-23** (striped bars) or tolcapone (empty bars) as measured by the PrestoBlue assay. The values correspond to mean ± SEM (*n* = 3).

In this study, HeLa and HepG2 cells were exposed to increasing concentrations of **M-23** or tolcapone for 72 h at 37 °C using the PrestoBlue cell viability reagent. For both cell lines, **M-23** showed significantly lower toxicity than tolcapone above 10 μM compound concentration (Figure 7). These results are biologically relevant, particularly for HepG2, as they recapitulate tolcapone’s *in vivo* hepatotoxicity and suggest that M-23 is not only a better TTR stabilizer but might also overcome one of the major concerns related with tolcapone therapy.

### **M-23** Stabilizes the TTR Dimer–Dimer Interface

**Figure 8:**
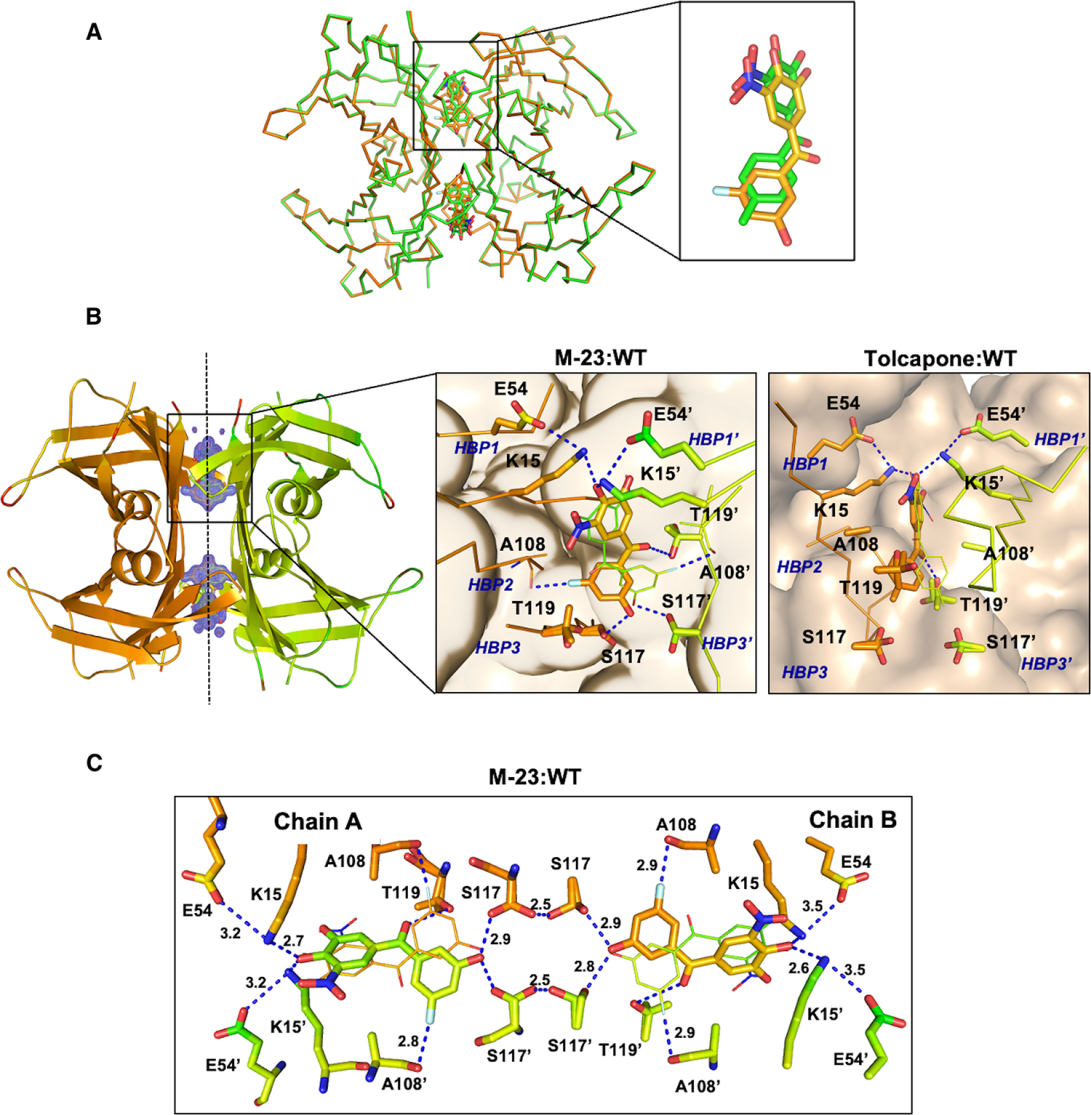
Crystal structure of WT-TTR complexed with **M-23**. (A) Superposition of WT-TTR complexed with **M-23** (orange) or tolcapone (green). Cα rmsd from 116 residues is 0.19 Å. A close-up view of one conformation of the superposed compounds is shown on the inset. (B) General view of WT-TTR bound to **M-23** at 1.2 Å, represented as cartoon. The electron density maps of the two binding sites of **M-23** are shown. Dashed line depicts the twofold symmetry axis of the dimer–dimer interface. The insets represent the close-up view of one of the WT-TTR T_4_-binding sites for **M-23** and for tolcapone (PDB: 4D7B). Ligands and the most important interacting residues are illustrated as sticks. (C) **M-23** binding at the WT-TTR dimer–dimer interface. **M-23** and some of the TTR residues interacting with the ligand are represented by sticks. Dashed lines represent key interactions between **M-23** and WT-TTR and between the hydroxyl groups of S117/S117′.

The MD simulations, together with the thermodynamic analysis, point to a higher number and/or strength of interactions established between **M-23** and the residues within the TTR T_4_-binding sites as responsible for the enhanced affinity and the high enthalpic contribution to binding in this molecule with respect to tolcapone. To inspect this possibility, we determined the crystal structure of TTR with **M-23** at 1.2 Å resolution (Figure 8). The atomic coordinates have been deposited in the PDB (PDB code 7QC5). This high-resolution crystal allows one to unequivocally place **M-23** in the AB/CD dimer–dimer interface in the forward binding mode. As a result of the twofold symmetry of the binding sites, **M-23** adopts two equivalent binding modes related by a 180° rotation. As designed, **M-23** sits deeper in the innermost section of the hormone-binding cavity relative to tolcapone (Figure 8).

As in tolcapone, the 3,4-dihydroxy-5-nitrophenyl ring of **M-23** is oriented to the outer binding cavity, being surrounded by the hydrophobic residues from the HBPs 2/2′ and 1/1′ (Figure 8B). Remarkably, the ε-amino group of Lys15 is placed between the two hydroxyl groups of the phenyl ring of **M-23** and the carboxylate group of Glu54, establishing important electrostatic interactions. These interactions close the cavity around **M-23**, protecting the compound and the interactions it establishes with the protein, from the solvent. Although **M-23** is more buried into the cavity than tolcapone, the distances between Lys15 and the −OH are equal or shorter, resulting in Lys15 moving inward the cavity, whereas Glu54 keeps essentially the same position (Figure S4).

As in the TTR–tolcapone structure, the **M-23** central carbonyl group establishes a specific H-bond with the hydroxyl side chain of Thr119. As indicated by MD simulations, the 3-fluoro-5-hydroxyphenyl ring projects deep within the inner cavity where it participates in hydrophobic and van der Waals interactions with residues forming the two symmetrical T_4_-HBPs, HBP2 and HBP3 (Ala108, Leu110, Ser117, and Thr119). In addition, the **M-23** 5-OH group forms a short hydrogen bond with Ser117 (2.7/2.8 Å), and the 3-F substituent is in contact with Ala108 (2.8/2.8 Å). Such a short F···Ala108 distance matches the shortest one observed in the MD simulation (2.76 Å). These two interactions are absent in the TTR–tolcapone and the TTR–tafamidis structures, confirming that, as designed, **M-23** establishes a higher number of noncovalent H-bond contacts with the protein. They are expected to be stronger because they are buried in the low dielectric hydrophobic interior of the T_4_-binding cavity. These new contacts are likely the driving force for **M-23** higher affinity and exceptional enthalpy for binding.

Interestingly, the H-bond interactions of **M-23** with Ser117 in HBP3 and HBP3′ mimic the ones observed between these two residues in the kinetically stable T119M-TTR variant. These H-bond interactions in the two symmetric cavities help bring the dimer subunits closer and strengthen the molecular contacts between them, increasing the energy barrier for dissociation.^76,77^ Indeed, the O–O distances between facing Ser117 residues in the two dimers are shorter in T119M-TTR (A–C = 4.7 Å, B–D = 4.8 Å) than in WT-TTR (A–C = 5.5 Å, B–D = 5.4 Å). In the same manner, these distances are shorter in the TTR/**M-23** structure (A–C = 5.0 Å, B–D = 4.8 Å) than in the TTR/tolcapone complex (A–C = 5.4 Å, B–D = 5.4 Å). Actually, Ser117-compound contacts seem to hold the key for the enthalpy-driven stabilization of TTR by AG10,^38,68^ a novel TTR ligand that has already shown effectivity in the clinic for TTR cardiomyopathy.^78^ In one of the Ser117 side-chain conformations, they can establish a short hydrogen intradimer H-bond (2.5 Å), which might further contribute to stabilize the quaternary structure (Figure 8C).

## Conclusions

We have developed **M-23**, a disubstituted and halogenated tolcapone congener. This novel TTR kinetic stabilizer keeps the interactions established by the parental molecule in the outer and central sides of the binding cavity while being more internalized and establishing new and specific contacts with the innermost residues. Under the same conditions, tolcapone exhibits a superior stabilizing and anti-aggregational activity than tafamidis. The advantage of tolcapone is that, although it binds worse than tafamidis to the first TTR T_4_-binding site, it binds significantly better to the second one due to its lack of negative cooperativity. Here, we show that due to the unique network of interactions it establishes with TTR, **M-23** displays a higher affinity than any of these two therapeutically relevant molecules for both binding sites, becoming one of the strongest TTR ligands described so far. Accordingly, **M-23** stabilizes and protects TTR from aggregation *in vitro* at very low compound concentrations. Furthermore, the binding of **M-23** is entirely enthalpy driven, displaying an enthalpic contribution to the binding significantly higher than those of tafamidis and tolcapone, a parameter associated with the molecule potency and selectivity. Consequently, **M-23** strongly binds to TTR in human plasma, exhibiting a higher TTR stabilizing activity than the two reference molecules, thus becoming a candidate for further preclinical and clinical investigation. Crucial to identify **M-23** has been the application of MD simulations on top of rationally designed tolcapone variants since modeling the flexibility of the T_4_-binding cavity has allowed one to anticipate protein–compound interactions at atomic resolution and rank the molecules according to the energetics of binding. This integral approach constitutes a time- and cost-effective strategy to assist in the search of potent ATTR disease modifiers, allowing the evaluation of small molecules rapidly and accurately.

## Experimental Section

### Computational Simulations

Binding energies have been estimated from gas-phase interaction energies of a collection of frames generated from a classical molecular dynamics (MD) simulation and including solvent effects as the ligand solvation energy. This means that we assumed that the complex and the receptor have approximately the same solvation energy and that the solvation contribution to the binding energy only arises from ligands’ desolvation. MD simulations were performed with the Amber suite^79^ using the ff14SB force field.^80^ Organic ligands were parametrized using the gaff2 force field.^81^

Production was run for 150 ns in the NPT ensemble at a constant temperature of 300 K, and binding energies were estimated from 140 structures evenly sampled from the last 140 ns. Ligand solvation energies were computed with the SMD continuum model^82^ at the DFT (B3LYP/6-31+G(d,p)) level of theory with the Gaussian09 program.^83^

Models were built by replacing tolcapone molecules with their derivatives in the high-resolution X-ray diffraction tetrameric WT-TTR-tolcapone complex structure PDB: 4D7B.^39^ Complex structures were neutralized with the appropriate number of Na^+^ counterions, and water molecules were added up to a minimum distance of 8 Å from the protein. It should be noted that while in the tetrameric WT-TTR-tolcapone crystal structure the two ligands are symmetrically placed, simulations without any structural constraints led to an asymmetric organization with one ligand at the center of the TTR tetramer and the second in the binding channel, though further exposed to the solvent than in the crystallographic structure (Figure S5). This striking difference between our simulations and the X-ray structure may be due either to (i) a dynamic disorder of the two ligands in the complex in solution, which collapses to a symmetric organization upon crystallization, or (ii) the inadequacy of the molecular mechanics model to properly describe the system’s structural features. Thus, we decided to enforce a small harmonic constraint on the protein backbone so that the structure does not drift significantly apart from the crystallographic one. After extensive testing, we found that when including a constraint of 2 kcal/mol^−1^ Å^−2^ on the backbone, the symmetry of the two binding sites is preserved while allowing a reorganization of the ligand binding residues. Thus, this is the protocol adopted all along this work.

#### Molecular Dynamics Protocol

The molecular dynamics protocol includes (i) a 200 ps equilibration run in the NVT ensemble, raising the temperature from 0 to 100 K; (ii) a 2 ns equilibration run in the NPT ensemble, raising the temperature from 100 to 300 K; and (iii) a 150 ns production run in the NPT ensemble with the temperature kept constant to 300 K. A Langevin thermostat and a Monte Carlo barostat were employed. Hydrogen bonds were calculated with cpptraj default values (distance cutoff of 3 Å) in all cases except for Cl···X contacts for which the threshold was set to 3.5 Å. Visualization and postprocessing were done with VMD and MDtraj packages.^84,85^

### General Methods and Compound Characterization

Commercially available reagents were used as received. Solvents were dried by distillation over the appropriate drying agents. All reactions were monitored by analytical thin-layer chromatography (TLC) using silica gel 60 precoated aluminum plates (0.20 mm thickness). Flash column chromatography was performed using silica gel Geduran SI 60 (40–63 μm). ^1^H NMR and ^13^C NMR spectra were recorded at 250, 360, 400 MHz and 90, 100 MHz, respectively. ^19^F NMR spectra were recorded at 250 MHz. Proton chemical shifts are reported in ppm (δ) (CDCl_3_; δ 7.26, acetone-*d*_6_; δ 2.05, dimethyl sulfoxide (DMSO)-*d*_6_; δ 2.50, methanol-*d*_4_; δ 3.31). Carbon chemical shifts are reported in ppm (δ) (CDCl_3_; δ 77.16, acetone-*d*_6_; δ 29.84, DMSO-*d*_6_; δ 39.52 methanol-*d*_4_; δ 49.00). NMR signals were assigned with the help of HSQC, HMBC, and DEPT135. ^1^H NMR and ^13^C NMR spectra of new compounds are shown in Figures S6-S27. Melting points were determined on a hot stage and are uncorrected. HRMS was recorded using electrospray ionization. All final compounds are >95% pure by HPLC analysis (Figures S28-S32).

### General Synthesis Procedure to Prepare Compounds **M-14** to **M-23**

Tolcapone derivatives **M-14** to **M-23** were chemically synthesized as described in Scheme 1. The synthesis started with the coupling reaction of different preformed aryl Grignard reagents, from the corresponding iodoarene derivatives **1a**–**e**, to the known methyl diprotected catechol **2** to furnish alcohols **3a**–**e** in 49–81% yield. The Grignard reagents were freshly prepared by an iodine–magnesium exchange reaction^86^ with *i*-PrMgBr at −40 °C in THF of the iodoarene derivatives **1a**–**e** that were commercially available except for 3-fluoro-5-iodomethoxybenzene **1e**, obtained from 3-fluoro-5-methoxyaniline through the appropriated arenediazonium chloride and an ion exchange reaction with KI in 77% yield.^87^ Catechol **2** was prepared in excellent yield by the methylation of commercially available 5-nitrovainillin using a phase-transfer catalytic process.^88,89^ The following Dess–Martin periodinane oxidation on **3a**–**e** provided ketones **4a**–**e** in 70–99% yield.

Removal of both methyl protecting groups of the catechol moiety was first attempted under conventional conditions using boron tribromide in CH_2_Cl_2_.^90^ However, under these standard conditions, a complex mixture of products was obtained. The presence of the relatively sensitive nitro-group prevents the use of other routine reagents. After some experimentation, it was found that by controlling the reaction time and the equivalents of BBr_3_, a single ether cleavage was promoted delivering the expected monoprotected catechol. These optimized conditions were applied to the methyl deprotected compounds **4a**–**d** to furnish monoprotected catechols **5a**–**d** in 50–91% yield. For compound **4e**, the applied reaction conditions also led to the removal of the methyl protecting group of R_1_, delivering compound **5e** in 81% yield.

It has been described that the demethylation of ortho-hydroxy nitroarylmethyl ethers can be accomplished in good yields by a milder procedure in the presence of aluminum chloride (AlCl_3_).^91,92^ Accordingly, treatment of **5a**–**e** with AlCl_3_ and pyridine in refluxing chloroform smoothly provided the target tolcapone analogues **M-14**, **M-17**, **M-20**, **M-21**, and **M-23** in reasonably good yields (46–81%).

### Synthesis Procedure

Tolcapone was purchased from Fisher. All tolcapone derivatives used in this study were prepared as described below.

#### 3,4-Dimethoxy-5-nitrobenzaldehyde, **2**

To a solution of 5-nitrovanillin (500 mg, 2.50 mmol), NaOH 3 M (1.7 mL, 5.00 mmol), and TBAB (82 mg, 0.25 mmol) in a mixture of CH_2_Cl_2_/H_2_O (1:1, 10 mL), dimethyl sulfate (1.3 mL, 13.40 mmol) was added slowly under a nitrogen atmosphere. The final mixture was stirred vigorously for 24 h (TLC, hexane/EtOAc 3:2). The aqueous layer was extracted with CH_2_Cl_2_ (3 × 10 mL). Then, the organic layer was concentrated under a vacuum and washed with water (20 mL), a 2 M ammonia solution (20 mL), and a 2 M NaOH solution (20 mL) to remove unreacted phenol and dimethyl sulfate. The organic layer was dried over anhydrous Na_2_SO_4_ and concentrated under a vacuum. Purification by flash column chromatography using a mixture of hexane/EtOAc (3:2) furnished **2** as a pale brown solid (498 mg, 2.36 mmol, 93% yield). ^1^H NMR (250 MHz, CDCl_3_) δ 9.91 (s, 1H, −COH), 7.83 (d, *J*_6,2_ = 1.8 Hz, 1H, H-6), 7.62 (d, *J*_2,6_ = 1.8 Hz, 1H, H-2), 4.08 (s, 3H, C*H*_3_O-4), 4.00 (s, 3H, C*H*_3_O-3). The spectroscopic data were consistent with the literature.^88^

#### 1-Fluoro-3-iodo-5-methoxybenzene, **1e**

To a solution of 3-fluoro-5-methoxyaniline (1.02 g, 7.08 mmol) in H_2_O (3.3 mL) was added concentrated HCl (3.3 mL) at 0 °C. After stirring for 30 min, a 1.8 M solution of NaNO_2_ in H_2_O (4.5 mL, 8.14 mmol) was added dropwise. The resulting mixture was stirred for 15 min at 0 °C, and then an ice-cold 3 M solution of KI (5 mL) was added slowly. The ice bath was removed, and the reaction mixture was heated at the reflux temperature for 1 h. The reaction mixture was allowed to cool to room temperature (rt) and extracted with EtOAc (3 × 33 mL). The organic layer was washed with brine (2 × 50 mL), dried over anhydrous Na_2_SO_4_, and concentrated under a vacuum. The residue was purified by flash column chromatography (hexanes 100%) to afford **1e** as a colorless oil (1.37 g, 5.41 mmol, 77% yield). ^1^H NMR (400 MHz, CDCl_3_) δ 7.05–7.02 (m, 2H, H-2, H-4), 6.58 (dt, *J*_6,F_ = 10.6 Hz, *J*_6,4_ = 2.3 Hz, 1H, H-6), 3.78 (s, 3H, C*H*_3_O-5); ^19^F NMR (250 MHz, CDCl_3_) δ −110.76 (s, F-3″); ^13^C NMR (100.5 MHz, CDCl_3_) δ 163.2 (d, *J*_1,F_ = 250.0 Hz, C_1_), 161.4 (d, *J*_5,F_ = 11.1 Hz, C_5_), 119.5 (d, *J*_4,F_ = 3.2 Hz, C_4_), 117.4 (d, *J*_2,F_ = 24.0 Hz, C_2_), 102.0 (d, *J*_6,F_ = 25.2 Hz, C_6_), 93.3 (d, *J*_3,F_ = 11.0 Hz, C_3_), 55.9 (*C*H_3_O-5). IR (ATR) ν 2941, 1738, 1601, 1578, 1423, 1277, 1143 cm^–1^.

#### (3,4-Dimethoxy-5-nitrophenyl)[4-(trifluoromethyl)phenyl]methanol, **3a**

To a solution of 4-iodobenzotrifluoride, **1a** (100 μL, 0.68 mmol), in dry THF (1 mL) at −40 °C, *i-*PrMgBr (1 M in THF, 680 μL, 0.68 mmol) was added dropwise in 5 min under a nitrogen atmosphere, and the reaction mixture was stirred at the same temperature for 1 h. Then, a solution of 3,4-dimethoxy-5-nitrobenzaldehyde, **2** (151 mg, 0.72 mmol), in dry THF (1.2 mL) was added. The final mixture was warmed to rt and stirred overnight (ON). The reaction was quenched by slow addition of brine (4 mL), and the aqueous layer was extracted with EtOAc (3 × 10 mL). All organic layers were collected, dried over anhydrous Na_2_SO_4_, and concentrated under a vacuum, obtaining a yellow oil that was purified by flash column chromatography (hexanes/EtOAc, 2:1) to furnish **3a** (167 mg, 0.47 mmol, 69% yield) as an orange oil. ^1^H NMR (400 MHz, CDCl_3_) δ 7.61 (d, *J_3″,2″_* = 8.1 Hz, 2H, H-3″), 7.48 (d, *J*_2*″*,3″_ = 8.1 Hz, 2H, H-2″), 7.29 (dd, *J_6′,2_* = 2.0 Hz, *J_6′,1_* = 0.7 Hz, 1H, H-6′), 7.09 (d, *J*_2*′*,6_ = 2.0 Hz, 1H, H-2′), 5.83 (s, 1H, H-1), 3.93 (s, 3H, C*H*_3_O-4′), 3.88 (s, 3H, C*H*_3_O-3′), 2.77 (br s, 1H, −OH); ^19^F NMR (250 MHz, CDCl_3_) δ −63.04 (s, −CF_3_); ^13^C NMR (100.6 MHz, CDCl_3_) δ 154.4 (C_3*′*_), 146.5 (C_1″_), 144.6 (C_5*′*_), 142.2 (C_4*′*_), 139.5 (C_1*′*_), 130.4 (q, *J*_4″,F_ = 32.2 Hz, C_4″_), 126.8 (C_2″_, C_6″_, C_5″_), 125.9 (q, *J*_3″,F_ = 3.8 Hz, C_3″_), 124.1 (q, *J*_*C*F3,F_ = 272.0 Hz, −CF_3_), 114.0 (C_2*′*_), 113.8 (C_6*′*_), 74.7 (C_1_), 62.1 (*C*H_3_O-4′), 56.5 (*C*H_3_O-3′). IR (ATR) ν 3422, 1534, 1325, 1165, 1124 cm^–1^. HRMS (ESI+) calcd for [C_16_H_14_F_3_NO_5_ + H]^+^ ([M + H]^+^) 358.2932, found 358.0900.

#### (3,4-Dimethoxy-5-nitrophenyl)(4-fluorophenyl)methanol, **3b**

Compound **3b** was prepared as described for **3a** by using a solution of 4-fluoroiodobenzene, **1b** (200 μL, 0.90 mmol), in dry THF (2 mL), *i*-PrMgBr (1 M in THF, 900 μL, 0.90 mmol), and a solution of **2** (210 mg, 0.99 mmol) in dry THF (2.2 mL). Purification by flash column chromatography (hexanes/EtOAc, 4:1) afforded **3b** (213 mg, 0.69 mmol, 77% yield) as a colorless oil. ^1^H NMR (400 MHz, acetone-*d*_6_) δ 7.50 (br ddd, *J*_*2*″*,3*_ = *J*_*6*″*,5*_ = 9.0 Hz, *J*_*2*″*,F*_ = *J*_*6*″*,1*_ = 5.5 Hz, *J*_2″,1_ = *J*_6″,1_ = 0.6 Hz, 2H, H-2″, H-6″), 7.41 (d, *J*_2*′*,6_ = 2.0 Hz, 1H, H-2′), 7.38 (dd, *J*_6*′*,2_ = 2.0 Hz, H-6′), 7.09 (br t, *J*_2″,F_ = *J*_2*′*,6_ = *J*_3″,2_ = *J*_5″,6_ = 9.0 Hz, 1H, H-3″, H-5″), 5.91 (d, *J*_1,OH_ = 3.3 Hz, 1H, H-1), 5.26 (d, *J*_1,OH_ = 3.3 Hz, 1H, −OH), 3.93 (s, 3H, C*H*_3_O-4′), 3.90 (s, 3H, C*H*_3_O-3′); ^19^F NMR (250 MHz, CDCl_3_) δ −117.36 (m, F-4″); ^13^C NMR (100.6 MHz, acetone-*d*_6_) δ 162.9 (d, *J*_4″,F_ = 243.6 Hz, C_4″_), 154.8 (C_3*′*_), 145.8 (C_5*′*_), 142.9 (C_1*′*_), 141.7 (C_3*′*_), 141.5 (C_1″_), 129.3 (d, *J*_2″,F_ = *J*_6″,F_ = 7.8 Hz, C_2″_, C_6″_), 115.8 (d, *J*_4″,F_ = 21.6 Hz, C_3″_, C_5″_), 115.0 (C_2*′*_), 113.6 (C_6*′*_), 74.4 (C_1_), 62.0 (*C*H_3_O-4′), 56.9 (*C*H_3_O-3′). IR (ATR) ν 3416, 1531, 1359, 1280, 1223 cm^–1^. HRMS (ESI+) calcd for [C_15_H_14_FNO_5_ + H]^+^ ([M + H]^+^) 308.0934, found 308.0931.

#### (3,5-Dichlorophenyl)(3,4-dimethoxy-5-nitrophenyl)methanol, **3c**

Compound **3c** was prepared as described for **3a** by using a solution of 3,5-dichloroiodobenzene, **1c** (558 mg, 2.04 mmol), in dry THF (5 mL), *i*-PrMgBr (1 M in THF, 2.0 mL, 2.00 mmol), and a solution of **2** (360 mg, 1.70 mmol) in dry THF (5 mL). Purification by flash column chromatography (hexanes/EtOAc, 1:1) furnished **3c** (427 mg, 1.19 mmol, 70% yield) as an orange solid. Mp 100–103 °C (from acetone). ^1^H NMR (400 MHz, acetone-*d*_6_) δ 7.50 (dd, *J*_2″,4_ = *J*_6″,4_ = 1.9 Hz, *J*_2″,1_ = *J*_6″,1_ = 0.6 Hz, 2H, H-2″, H-6″), 7.48 (d, *J_2′,6_* = 2.0 Hz, 1H, H-2′), 7.44 (dd, *J*_6*′*,2_ = 2.0 Hz, *J*_6*′*,1_ = 0.6 Hz, 1H, H-6′), 7.35 (t, *J*_4″,2_ = *J*_4″,6_ = 1.9 Hz, 1H, H-4″), 5.95 (d, *J*_1,OH_ = 3.8 Hz, 1H, H-1), 5.50 (d, J_OH,1_ = 3.9 Hz, 1H, −OH), 3.95 (s, 3H, C*H*_3_O-3′), 3.90 (s, 3H, C*H*_3_O-4′); ^13^C NMR (90.5 MHz, acetone-*d*_6_) δ 155.0 (C_3*′*_), 149.5 (C_1*′*_), 145.9 (C_5*′*_), 142.0 (C_4*′*_), 141.7 (C_1″_), 135.5 (C_3″_, C_5″_), 127.9 (C_4″_)_,_ 125.9 (C_2″_, C_6″_), 115.0 (C_2*′*_), 113.7 (C_6*′*_), 73.9 (C_1_), 62.0 (*C*H_3_O-4′), 57.0 (*C*H_3_O-3′). IR (ATR) ν 3470, 2948, 1530, 1431, 1370 cm^–1^. HRMS (ESI−) calcd for [C_15_H_13_Cl_2_NO_5_-H_2_O]^−^ ([M-H_2_O]^−^) 340.0143, found 340.0145.

#### (3,5-Difluorophenyl)(3,4-dimethoxy-5-nitrophenyl)methanol, **3d**

Compound **3d** was prepared as described for **3a** by using a solution of 3,5-difluoroiodobenzene, **1d** (555 mg, 2.31 mmol), in dry THF (5 mL), *i*-PrMgBr (1 M in THF, 2.5 mL, 2.5 mmol), and a solution of **2** (582 mg, 2.78 mmol) in dry THF (5 mL). Purification by flash column chromatography (hexanes/EtOAc, 3:1) afforded **3d** (612 mg, 1.88 mmol, 81% yield) as an orange oil. ^1^H NMR (400 MHz, CDCl_3_) δ 7.25 (d, *J_6′,2_* = 2.0 Hz, 1H, H-6′), 7.07 (d, *J*_2*′*,6_ = 2.0 Hz, 1H, H-2′), 6.87 (m, *J*_2″,F_ = *J*_6″,F_ = 6.0 Hz, 2H, H-2″, H-6″), 6.70 (tt, *J*_4″,2_ = *J*_4″,6″_ = 2.4 Hz, *J*_4″,F_ = 8.9 Hz, 1H, H-4″), 5.71 (br s, 1H, H-1), 3.92 (s, 3H, C*H*_3_O-4′), 3.88 (s, 3H, C*H*_3_O-3′), 3.10 (br s, 1H, −OH); ^19^F NMR (250 MHz, CDCl_3_) δ −108.97 (m, F-3″, F-5″); ^13^C NMR (100.6 MHz, CDCl_3_) δ 163.2 (dd, *J*_3″,F_ = *J*_5″,F_ = 12.5 Hz, *J*_3″,F_ = *J*_5″,F_ = 249.7 Hz, C_3″_,C_5″_), 154.3 (C_3*′*_), 146.6 (t, *J*_1″,F_ = 8.6 Hz, C_1″_), 144.4 (C_5*′*_), 142.2 (C_4*′*_), 139.3 (C_1*′*_), 114.0 (C_2*′*_), 113.8 (C_6*′*_), 109.4 (m, *J*_2″,F_ = *J*_6″,F_ = 18.7 Hz, C_2″_, C_6″_), 103.4 (t, *J*_4″,F_ = 25.3 Hz, C_4″_), 74.2 (C_1_), 62.1 (*C*H_3_O-4′), 56.5 (*C*H_3_O-3′). IR (ATR) ν 3415, 1598, 1534, 1281 cm^–1^. HRMS (ESI+) calcd for [C_15_H_13_F_2_NO_5_ + Na]^+^ ([M + Na]^+^) 348.0659, found 348.0663.

#### (3,4-Dimethoxy-5-nitrophenyl)(3-fluoro-5-methoxyphenyl)methanol, **3e**

Compound **3e** was prepared as described for **3a** by using a solution of 1-fluoro-3-iodo-5-methoxybenzene, **1e** (308 mg, 1.22 mmol), in dry THF (3 mL), *i*-PrMgBr (1 M in THF, 1.9 mL, 1.90 mmol), and a solution of **2** (310 mg, 1.20 mmol) in dry THF (4 mL). Purification by flash column chromatography (hexanes/EtOAc, 5:1) afforded **3e** (191 mg, 0.57 mmol, 49% yield) as a white solid. Mp 100–101 °C (from CHCl_3_). ^1^H NMR (400 MHz, CDCl_3_) δ 7.28 (dd, *J_6′,2_* = 2.0 Hz, *J_6′,1_* = 0.6 Hz, 1H, H-6′), 7.11 (d, *J*_2*′*,6_ = 2.0 Hz, 1H, H-2′), 6.69 (m, H-6″), 6.65 (br dm, *J*_2″,F_ = 9.1 Hz, *J*_2″,4_ = *J*_2″,6_ = 2.0 Hz, 1H, H-2″), 6.53 (dt, *J*_4″,F_ = 10.5 Hz, *J*_4″,2_ = *J*_4″,2_ = 2.3 Hz, 1H, H-4″), 5.70 (s, 1H, H-1), 3.94 (s, 3H, C*H*_3_O-4′), 3.88 (s, 3H, C*H*_3_O-3′), 3.78 (s, 3H, C*H*_3_O-5″), 2.72 (br s, 1H, −OH); ^19^F NMR (250 MHz, CDCl_3_) δ −110.76 (s, F-3″); ^13^C NMR (100.6 MHz, CDCl_3_) δ 163.9 (d, *J*_3″,F_ = 246.3 Hz, C_3″_), 161.3 (d, ^3^*J*_5″,F_ = 11.2 Hz, C_5″_), 154.3 (C_3*′*_), 145.8 (d, *J*_1″,F_ = 8.8 Hz, C_1″_), 144.6 (s, C_5*′*_), 142.2 (s, C_4*′*_), 139.5 (C_1*′*_), 114.0 (C_2*′*_), 113.8 (C_6*′*_), 108.4 (d, *J*_6″,F_ = 3.8 Hz, C_6″_), 105.7 (d, *J*_2″,F_ = 22.7 Hz, C_2″_), 101.1 (d, *J*_4″,F_ = 25.4 Hz, C_4″_), 74.8 (d, *J*_1,F_ = 2.3 Hz, C_1_), 62.1 (*C*H_3_O-4′), 56.6 (*C*H_3_O-3′), 55.7 (*C*H_3_O-5″). IR (ATR) ν 3230, 2837, 1594, 1530, 1453, 1344, 1133 cm^–1^. HRMS (ESI−) calcd for [C_16_H_16_FNO_6_-H_2_O]^−^ ([M-H_2_O]^−^) 320.0934, found 320.0936.

#### (3,4-Dimethoxy-5-nitrophenyl)[4-(trifluoromethyl)phenyl]methanone, **4a**

To a solution of alcohol **3a** (167 mg, 0.52 mmol) in dry CH_2_Cl_2_ (2 mL) under a nitrogen atmosphere, a solution of DMPI (338 mg, 1.07 mmol) in CH_2_Cl_2_ (3 mL) was added dropwise, and the mixture was stirred ON at RT. The reaction was quenched with the addition of 4 mL of a prepared solution of Na_2_S_2_O_3_ (1.13 g) in a saturated aqueous solution of NaHCO_3_ (6 mL), and the mixture was stirred for 15 min. The aqueous phase was extracted with CH_2_Cl_2_ (3 × 15 mL), and the combined organic extracts were dried over anhydrous Na_2_SO_4_ and concentrated under a vacuum. Purification by flash column chromatography (hexanes/EtOAc, 3:1) of the resulting residue provided ketone **4a** (131 mg, 0.37 mmol, 70% yield) as a white solid. Mp 118–119 °C (from CH_2_Cl_2_). ^1^H NMR (400 MHz, CDCl_3_) δ 7.87 (d, *J*_2″,3_ = 8.2 Hz, 2H, H-2″), 7.79 (d, *J*_3″,2_ = 8.2 Hz, 2H, H-3″), 7.67 (d, *J_2′,6_* = 2.0 Hz, 1H, H-2′), 7.65 (d, *J*_6*′*,2_ = 2.0 Hz, 1H, H-6′), 4.08 (s, 3H, C*H*_3_O-4′), 4.00 (s, 3H, C*H*_3_O-3′); ^19^F NMR (250 MHz, CDCl_3_) δ −63.58 (s, −CF_3_); ^13^C NMR (100.6 MHz, CDCl_3_) δ 192.7 (C_1_), 154.6 (C_3*′*_), 147.0 (C_4*′*_), 144.2 (C_5*′*_), 139.8 (C_1″_), 134.4 (q, *J*_4″,F_ = 33.2 Hz, C_4″_), 131.7 (C_1*′*_), 130.1 (C_2″_), 125.9 (q, *J*_3″,F_ = 3.9 Hz, C_3″_), 123.6 (q, *J*_*C*F3,F_ = 272.6 Hz, −*C*F_3_), 118.9 (C_6*′*_), 116.2 (C_2*′*_), 62.4 (*C*H_3_O-4′), 56.9 (*C*H_3_O-3′). IR (ATR) ν 3086, 2950, 2840, 1655, 1531, 1364, 1323, 1292, 1243, 1163 cm^–1^. HRMS (ESI+) calcd for [C_16_H_12_F_3_NO_5_ + H]^+^ ([M + H]^+^) 356.0746, found 356.0740.

#### (3,4-Dimethoxy-5-nitrophenyl)(4-fluorophenyl)methanone, **4b**

Compound **4b** was prepared as described for **4a** by using alcohol **3b** (230 mg, 0.75 mmol) in CH_2_Cl_2_ (3 mL) and a solution of DMPI (475 mg, 1.12 mmol) in CH_2_Cl_2_ (5 mL). Purification by flash column chromatography (hexanes/EtOAc, 3:1) afforded **4b** (226 mg, 0.74 mmol, 99% yield) as a brown solid. Mp 73–75 °C (from CHCl_3_). ^1^H NMR (400 MHz, DMSO-*d*_6_) δ 7.89 (br dd, *J*_*2*″*,3*_ = *J*_*6*″*,5*_ = 8.9 Hz, *J*_*2*″*,F*_ = *J*_*6*″*,F*_ = 5.5 Hz, 2H, H-2″, H-6″), 7.70 (d, *J*_6*′*,2_ = 2.0 Hz, 1H, H-6′), 7.66 (d, *J*_2*′*,6_ = 2.0 Hz, 1H, H-2′), 7.41 (br t, *J*_3″,F_ = *J*_5″,F_ = *J*_3″,2_ = *J*_5″,6_ = 8.9 Hz, 2H, H-3″, H-5″), 3.97 (s, 3H, C*H*_3_O-4′), 3.96 (s, 3H, C*H*_3_O-3′); ^19^F NMR (250 MHz, DMSO-*d*_6_) δ −106.21 (F-4″); ^13^C NMR (100.6 MHz, DMSO-*d*_6_) δ 191.8 (C_1_), 165.0 (d, *J*_4″,F_ = 252.0 Hz, C_4″_), 153.4 (C_3*′*_), 144.7 (C_4*′*_), 143.8 (C_5*′*_), 132.8 (d, *J*_2″,F_ = *J*_6″,F_ = 9.3 Hz, C_2″_, C_6″_), 132.7 (C_1″_), 132.4 (C_1*′*_), 117.2 (C_6*′*_), 116.9 (C_2*′*_), 115.9 (d, *J*_3″,F_ = *J*_5″,F_ = 22.1 Hz, C_3″_, C_5″_), 61.8 (*C*H_3_O-4′), 56.8 (*C*H_3_O-3′). IR (ATR) ν 2954, 1650, 1598, 1531, 1229 cm^–1^. HRMS (ESI+) calcd for [C_15_H_12_FNO_5_ + Na]^+^ ([M + Na]^+^) 328.0597, found 328.0595.

#### (3,5-Dichlorophenyl)(3,4-dimethoxy-5-nitrophenyl)methanone, **4c**

Compound **4c** was prepared as described for **4a** by using alcohol **3c** (374 mg, 1.00 mmol) in CH_2_Cl_2_ (4.7 mL) and a solution of DMPI (505 mg, 1.60 mmol) in CH_2_Cl_2_ (4.5 mL). Purification by flash column chromatography (hexanes/EtOAc, 3:1) afforded **4c** (268 mg, 0.75 mol, 73% yield) as a white solid. Mp 112–114 °C (from acetone). ^1^H NMR (400 MHz, DMSO-*d*_6_) δ 7.96 (m, 1H, H-4″), 7.75 (m, 2H, H-2″, H-6″), 7.75 (m, 1H, H-6′), 7.69 (d, *J*_2*′*,6_ = 2.0 Hz, H-2′), 3.98 (s, 3H, C*H*_3_O-4′), 3.96 (s, 3H, C*H*_3_O-3′); ^13^C NMR (100.6 MHz, DMSO-*d*_6_) δ 190.8 (C_1_), 153.5 (C_3*′*_), 145.3 (C_4*′*_), 143.9 (C_5*′*_), 139.6 (C_1″_), 134.6 (C_3″_, C_5″_), 132.1 (C_4″_), 131.3 (C_1*′*_), 128.0 (C_2″_, C_6″_), 117.9 (C_6*′*_), 116.9 (C_2*′*_), 61.9 (*C*H_3_O-4′), 56.9 (*C*H_3_O-3′). IR (ATR) ν 3077, 1659, 1535, 1365, 1290 cm^–1^. HRMS (ESI+) calcd for [C_15_H_11_Cl_2_NO_5_ + H]^+^ ([M + H]^+^) 356.0093, found 356.0087.

#### (3,5-Difluorophenyl)(3,4-dimethoxy-5-nitrophenyl)methanone, **4d**

Compound **4d** was prepared as described for **4a** by using alcohol **3d** (233 mg, 716 μmol) in CH_2_Cl_2_ (4 mL) and a solution of DMPI (456 mg, 1.07 mmol) in CH_2_Cl_2_ (5 mL). Purification by flash column chromatography (hexanes/EtOAc, 3:1) afforded **4d** (259 mg, 0.80 mmol, 75% yield) as a white solid. Mp 151–152 °C (from CH_2_Cl_2_). ^1^H NMR (360 MHz, CDCl_3_) δ 7.67 (d, *J_6′,2_* = 2.0 Hz, 1H, H-6′), 7.63 (d, *J*_2*′*,6_ = 2.0 Hz, 1H, H-2′), 7.28 (m, *J*_2″,F_ = *J*_6″,F_ = 5.2 Hz, 2H, H-2″, H-6″), 7.09 (tt, *J*_4″,2_ = *J*_4″,6_ = 2.2 Hz, *J*_4″,F_ = 8.5 Hz, 1H, H-4″), 4.09 (s, 3H, C*H*_3_O-4′), 4.00 (s, 3H, C*H*_3_O-3′); ^19^F NMR (250 MHz, CDCl_3_) δ −107.50 (t, *J* = 6.9 Hz, F-3″, F-5″); ^13^C NMR (90.5 MHz, CDCl_3_) δ 191.2 (br t, *J*_CO,F_ = 2.3 Hz, C_1_), 163.0 (dd, *J*_3″,F_ = *J*_5″,F_ = 12.0 Hz, *J*_3″,F_ = *J*_5″,F_ = 252.5 Hz, C_3″_, C_5″_), 154.6 (C_3*′*_), 147.0 (C_4*′*_), 144.2 (C_5*′*_), 139.6 (t, *J*_1″,F_ = 7.8 Hz, C_1″_), 131.4 (C_1*′*_), 118.7 (C_6*′*_), 116.2 (C_2*′*_), 112.9 (m, *J*_2″,F_ = *J*_6″,F_ = 18.6 Hz, C_2″_, C_6″_), 108.5 (t, *J*_4″,F_ = 25.5 Hz, C_4″_), 62.4 (*C*H_3_O-4′), 56.9 (*C*H_3_O-3′). IR (ATR) ν 3084, 1665, 1589, 1437, 1320 cm^–1^. HRMS (ESI+) calcd for [C_15_H_11_F_2_NO_5_ + Na]^+^ ([M + Na]^+^) 346.0503, found 346.0501.

#### (3,4-Dimethoxy-5-nitrophenyl)(3-fluoro-5-methoxyphenyl)methanone, **4e**

Compound **4e** was prepared as described for **4a** by using alcohol **3e** (287 mg, 0.85 mmol) in CH_2_Cl_2_ (4 mL) and a solution of DMPI (546 mg, 1.28 mmol) in CH_2_Cl_2_ (6 mL). Purification by flash column chromatography (hexanes/EtOAc, 5:2) afforded **4e** (199 mg, 0.59 mmol, 70% yield) as a white solid. Mp 95 °C (from CHCl_3_). ^1^H NMR (400 MHz, CDCl_3_) δ 7.69 (d, *J_6′,2_* = 2.0 Hz, 1H, H-6′), 7.63 (d, *J*_2*′*,6_ = 2.0 Hz, 1H, H-2′), 7.08 (m, H-6″), 7.02 (ddd, *J*_2″,F_ = 8.4 Hz, *J*_2″,4_ = 2.3 Hz, *J*_2″,6_ = 1.4 Hz, 1H, H-2″), 6.86 (dt, *J*_4″,F_ = 10.2 Hz, *J*_4″,2_ = *J*_4″,2_ = 2.3 Hz, 1H, H-4″), 4.07 (s, 3H, C*H*_3_O-4′), 3.99 (s, 3H, C*H*_3_O-3′), 3.85 (s, 3H, C*H*_3_O-5″); ^19^F NMR (250 MHz, CDCl_3_) δ −110.1 (s, F-3″); ^13^C NMR (90.5 MHz, CDCl_3_) δ 192.4 (d, *J*_1,F_ = 2.8 Hz C_1_), 163.3 (d, *J*_3″,F_ = 248.3 Hz, C_3″_), 161.1 (d, *J*_5″,F_ = 10.8 Hz, C_5″_), 154.4 (C_3*′*_), 146.7 (C_4*′*_), 144.2 (C_5*′*_), 139.0 (d, *J*_1″,F_ = 8.4 Hz, C_1″_), 132.0 (C_1*′*_), 118.7 (C_6*′*_), 116.3 (C_2*′*_), 111.2 (d, *J*_6″,F_ = 2.8 Hz, C_6″_), 109.1 (d, *J*_2″,F_ = 23.1 Hz, C_2″_), 106.4 (d, *J*_4″,F_ = 25.0 Hz, C_4″_), 62.4 (*C*H_3_O-4′), 56.8 (*C*H_3_O-3′), 56.1 (*C*H_3_O-5″). IR (ATR) ν 2921, 1597, 1530, 1429, 1318, 1146 cm^–1^. HRMS (ESI+) calcd for [C_16_H_14_FNO_6_ + Na]^+^ ([M + Na]^+^) 358.0703, found 358.0705.

#### (4-Hydroxy-3-methoxy-5-nitrophenyl)[4-(trifluoromethyl)phenyl]methanone, **5a**

To a solution of **4a** (359 mg, 1.01 mmol) in CH_2_Cl_2_ (5.5 mL) was added dropwise BBr_3_ (1 M in CH_2_Cl_2_, 8.0 mL, 8.0 mmol) at −10 °C. The reaction was allowed to proceed at RT for 2 h. The reaction mixture was quenched carefully with water (5 mL), and the resulting aqueous layer was extracted with EtOAc (2 × 20 mL). The organic extracts were dried with Na_2_SO_4_, and the solvent was removed under a vacuum to give the crude product. Purification by flash column chromatography (CH_2_Cl_2_/MeOH, 30:1) afforded **5a** (270 mg, 0.79 mmol, 78% yield) as a yellow solid. Mp 178–181 C (from MeOH). ^1^H NMR (400 MHz, DMSO-*d*_6_) δ 7.95–7.93 (m, 4H, H-2″, H-3″, H-5″, H-6″), 7.78 (d, *J_6′,2_* = 1.9 Hz, 1H, H-6′), 7.64 (d, *J*_2*′*,6_ = 1.9 Hz, 1H, H-2′), 3.96 (s, 3H, CH_3_O-3′); ^19^F NMR (250 MHz, CDCl_3_) δ −63.51 (−CF_3_); ^13^C NMR (100.6 MHz, DMSO-*d*_6_) δ 192.2 (C_1_), 149.7 (C_3*′*_), 146.9 (C_4*′*_), 140.5 (C_1″_), 136.5 (C_5*′*_), 132.0 (q, *J*_4″,F_ = 32.1 Hz, C_4″_), 130.1 (C_2″_, C_6″_), 125.9 (C_1*′*_), 125.6 (q, *J*_3″,F_ = *J*_5″,F_ = 4.2 Hz, C_3″_,C_5″_), 123.8 (q, *J*_CF3,F_ = 272.4, −*C*F_3_), 120.0 (C_2*′*_), 115.0 (C_6*′*_), 56.8 (*C*H_3_O-3′). IR (ATR) ν 3198, 2926, 1652, 1614, 1551, 1287 cm^–1^. HRMS (ESI−) calcd for [C_15_H_10_F_3_NO_5_-H]^−^ ([M – H]^−^) 340.0439, found 340.0439.

#### (4-Fluorophenyl)(4-hydroxy-3-methoxy-5-nitrophenyl)methanone, **5b**

Compound **5b** was prepared as described for **5a** by using ketone **4b** (259 mg, 0.85 mmol) in CH_2_Cl_2_ (4 mL) and BBr_3_ (1 M in CH_2_Cl_2_, 6.8 mL, 6.8 mmol). Purification by flash column chromatography (CH_2_Cl_2_/MeOH, 30:1) delivered **5b** (225 mg, 0.77 mmol, 91% yield) as a green solid. Mp 120–122 °C (from CH_2_Cl_2_). ^1^H NMR (400 MHz, methanol-*d*_4_) δ 7.92 (d, *J*_6*′*,2_ = 1.9 Hz, 1H, H-6′), 7.85 (br dd, *J*_*2*″*,3*_ = *J*_*6*″*,5*_ = 8.8 Hz, *J*_*2*″*,F*_ = *J*_*6*″*,F*_ = 5.4 Hz, 2H, H-2″, H-6″), 7.63 (d, *J*_2*′*,6_ = 1.8 Hz, 1H, H-2′), 7.28 (br t, *J*_3″,F_ = *J*_5″,F_ = *J*_3″,2_ = *J*_5″,6_ = 8.8 Hz, 2H, H-3″, H-5″), 3.98 (s, 3H, C*H*_3_O-3′); ^19^F NMR (250 MHz, methanol-*d*_4_) δ −108.63 (F-4″); ^13^C NMR (100.6 MHz, methanol-*d*_4_) δ 194.0 (C_1_), 166.8 (d, *J*_4″,F_ = 252.9 Hz, C_4″_), 151.8 (C_3*′*_), 150.2 (C_4*′*_), 136.4 (C_5*′*_), 134.9 (d, *J*_1″,F_ = 3.0 Hz, C_1″_), 133.5 (d, *J*_2″,F_ = *J*_6″,F_ = 9.2 Hz, C_2″_, C_6″_), 128.2 (C_1*′*_), 121.2 (C_6*′*_), 116.6 (d, *J*_3″,F_ = *J*_5″,F_ = 22.3 Hz, C_3″_, C_5″_), 116.5 (C_2*′*_), 57.3 (*C*H_3_O-3′). IR (ATR) ν 3170, 2924, 1599, 1547, 1229 cm^–1^. HRMS (ESI−) calcd for [C_14_H_10_FNO_5_-H]^−^ ([M – H]^−^) 290.0475, found 290.0465.

#### (3,5-Dichlorophenyl)(4-hydroxy-3-methoxy-5-nitrophenyl)methanone, **5c**

Compound **5c** was prepared as described for **5a** by using ketone **4c** (130 mg, 0.37 mmol) in CH_2_Cl_2_ (2 mL) and BBr_3_ (1 M solution in CH_2_Cl_2,_ 2.9 mL, 2.9 mmol). Purification by recrystallization from acetone furnished **5c** (98 mg, 0.28 mmol, 78% yield) as a yellow solid. Mp 204–205 °C (from acetone). ^1^H NMR (400 MHz, DMSO-*d*_6_) δ 7.94 (t, *J*_*4*″*,2*_ = *J*_*4*″*,6*_ = 2.0 Hz, 1H, H-4″), 7.78 (d, *J*_6*′*,2_ = 2.0 Hz, 1H, H-6′), 7.73 (d, *J*_*2*″*,4*_ = *J*_*6*″*,4*_ = 2.0 Hz, 2H, H-2″, H-6″), 7.59 (d, *J*_2*′*,6_ = 2.0 Hz, 1H, H-2′), 3.95 (s, 3H, C*H*_3_O-3′); ^13^C NMR (100.6 MHz, DMSO-*d*_6_) δ 190.5 (C_1_), 149.8 (C_3*′*_), 147.3 (C_4*′*_), 140.3 (C_5*′*_), 136.6 (C_1*′*_), 134.4 (C_3″_, C_5″_), 131.6 (C_4″_), 127.7 (C_2″_, C_6″_), 125.4 (C_1″_), 120.2 (C_6*′*_), 114.8 (C_2*′*_), 56.8 (*C*H_3_O-3′). IR (ATR) ν 3190, 3079, 1543, 1405, 1285 cm^–1^. HRMS (ESI−) calcd for [C_14_H_9_Cl_2_NO_5_-H]^−^ ([M – H]^−^) 339.9780, found 339.9786.

#### (3,5-Difluorophenyl)(4-hydroxy-3-methoxy-5-nitrophenyl)methanone, **5d**

Compound **5d** was prepared as described for **5a** by using ketone **4d** (216 mg, 0.67 mmol) in CH_2_Cl_2_ (4 mL) and BBr_3_ (1 M in CH_2_Cl_2_, 5.4 mL, 5.4 mmol). Purification by recrystallization from acetone afforded **5d** (103 mg, 0.33 mmol, 50% yield) as a yellow solid. Mp 190–192 °C (from acetone). ^1^H NMR (400 MHz, CDCl_3_) δ 11.18 (s, 1H, −OH), 8.08 (br d, *J_6′,2_* = 1.6 Hz, 1H, H-6′), 7.70 (br d, 1H, H-2′), 7.28 (m, 2H, H-2″, H-6″), 7.10 (tt, *J*_4″,2_ = *J*_4″,6_ = 2.2 Hz, *J*_4″,F_ = 8.5 Hz, 1H, H-4″), 4.04 (s, 3H, C*H*_3_O-3′); ^19^F NMR (250 MHz, CDCl_3_) δ −110.05 (t, *J* = 7.6 Hz, F-3″, F-5″); ^13^C NMR (100.6 MHz, CDCl_3_) δ 191.1 (C_1_), 163.0 (dd, *J*_3″,F_ = *J*_5″,F_ = 12.0 Hz, *J*_3″,F_ = *J*_5″,F_ = 252.5 Hz, C_3″_,C_5″_), 150.9 (C_3*′*_), 150.4 (C_4*′*_), 139.7 (t, *J*_1″,F_ = 7.8 Hz, C_1″_), 132.9 (C_5*′*_), 127.3 (C_1*′*_), 119.7 (C_6*′*_), 117.0 (C_2*′*_), 112.8 (m, *J*_2″,F_ = *J*_6″,F_ = 18.8 Hz, C_2″_, C_6″_), 108.4 (t, *J*_4″,F_ = 25.4 Hz, C_4″_), 57.2 (*C*H_3_O-3′). IR (ATR) ν 3098, 1590, 1326, 1242 cm^–1^. HRMS (ESI−) calcd for [C_14_H_9_F_2_NO_5_-H]^−^ ([M – H]^−^) 308.0371, found 308.0378.

#### 3-Fluoro-5-hydroxyphenyl)(4-hydroxy-3-methoxy-5-nitrophenyl)methanone, **5e**

Compound **5e** was prepared as described for **5a** by using ketone **4e** (199 mg, 0.56 mmol) in CH_2_Cl_2_ (3 mL) and BBr_3_ (1 M in CH_2_Cl_2_, 4.5 mL, 4.5 mmol). Purification by flash column chromatography (CH_2_Cl_2_/MeOH, 20:1) delivered **5e** (139 mg, 0.45 mmol, 81% yield) as a yellow solid. Mp 155–156 °C (from CHCl_3_). ^1^H NMR (250 MHz, CDCl_3_) δ 8.11 (d, *J_6′,2_* = 1.9 Hz, 1H, H-6′), 7.69 (d, *J*_2*′*,6_ = 1.9 Hz, 1H, H-2′), 7.04 (m, H-6″), 6.99 (ddd, *J*_2″,F_ = 8.4 Hz, *J*_2″,4_ = 2.3 Hz, *J*_2″,6_ = 1.4 Hz, 1H, H-2″), 6.85 (dt, *J*_4″,F_ = 9.4 Hz, *J*_4″,2_ = *J*_4″,2_ = 2.3 Hz, 1H, H-4″), 4.02 (s, 3H, C*H*_3_O-3′); ^19^F NMR (250 MHz, CDCl_3_) δ −110.01 (F-3″); ^13^C NMR (90.5 MHz, CDCl_3_) δ 192.3 (C_1_), 163.4 (d, *J*_3″,F_ = 248.5 Hz, C_3″_), 157.5 (d, *J*_5″,F_ = 11.8 Hz, C_5″_), 150.7 (C_3*′*_), 150.2 (C_4*′*_), 139.4 (d, *J*_1″,F_ = 8.1 Hz, C_1″_), 132.9 (C_5*′*_), 127.8 (C_1*′*_), 119.7 (C_6*′*_), 117.2 (C_2*′*_), 112.6 (d, *J*_6″,F_ = 2.7 Hz, C_6″_), 109.3 (d, *J*_2″,F_ = 23.1 Hz, C_2″_), 107.8 (d, *J*_4″,F_ = 25.0 Hz, C_4″_), 57.2 (*C*H_3_O-3′). IR (ATR) ν 3213, 2922, 1738, 1600, 1546, 1330, 1242, 1134 cm^–1^. HRMS (ESI−) calcd for [C_14_H_10_FNO_6_-H]^−^ ([M – H]^−^) 306.0414, found 306.0424.

#### (3,4-Dihydroxy-5-nitrophenyl)-[4-(trifluoromethyl)phenyl]methanone, **M-14**

To a mixture of monoprotected catechol **5a** (20 mg, 62 μmol) and AlCl_3_ (15 mg, 0.11 mmol) in dry CHCl_3_ (1 mL) in an argon atmosphere, pyridine (13 μL, 0.17 mmol) was added dropwise at 0 °C. The reaction mixture was heated to the reflux temperature and stirred until the starting material was consumed. The orange suspension was concentrated under a vacuum, and HCl (5 M, 2 mL) was added, keeping the temperature <25 °C. The aqueous layer was extracted with EtOAc (3 × 10 mL), and the combined organic yellow extracts were dried over anhydrous Na_2_SO_4_ and concentrated under a vacuum to give the crude product. Purification by recrystallization from acetone and water provided **M-14** (15 mg, 45 μmol, 75% yield) as a pale brown solid. Mp 68–71 °C (from CH_2_Cl_2_). ^1^H NMR (360 MHz, acetone-*d*_6_) δ 8.02–8.00 (m, *J*_2″,3_ = *J*_6″,5_ = 8.1 Hz, 3H, H-2″, H-6″, H-6′), 7.94 (br d, *J*_3″,2_ = *J*_6″,5_ = 8.1 Hz, 2H, H-3″, H-5″), 7.68 (d, *J*_2*′*,6_ = 1.9 Hz, 1H, H-2′); ^19^F NMR (250 MHz, acetone-*d*_6_) δ −68.53 (−CF_3_); ^13^C NMR (90.5 MHz, acetone-*d*_6_) δ 193.0 (C_1_), 148.9 (C_3*′*_), 148.2 (C_4*′*_), 141.6 (C_1″_), 135.3 (C_5′_), 133.8 (q, *J*_4″,F_ = 32.4 Hz, C_4″_), 130.9 (C_2″_, C_6″_), 128.5 (C_1*′*_), 126.4 (q, *J*_3″,F_ = *J*_5″,F_ = 3.8 Hz, C_3″_,C_5″_), 125.0 (q, *J*_CF3,F_ = 286.7, −CF_3_), 121.7 (C_2*′*_), 119.3 (C_6*′*_). IR (ATR) ν 3247, 1655, 1545, 1408, 1247 cm^–1^. HRMS (ESI−) calcd for [C_14_H_8_F_3_NO_5_-H]^−^ ([M – H]^−^) 326.0276, found 326.0284.

#### (3,4-Dihydroxy-5-nitrophenyl)-(4-fluorophenyl)methanone, **M-17**

Compound **M-17** was prepared as described for **M-14** by using **5b** (60 mg, 0.21 mmol), AlCl_3_ (57 mg, 0.41 mmol), and pyridine (50 μL, 0.62 mmol) in dry CHCl_3_ (3 mL). Purification by digestion in hexane afforded **M-17** (42 mg, 0.15 mmol, 70% yield) as a pale brown solid. Mp 162–165 °C (from CH_2_Cl_2_). ^1^H NMR (360 MHz, acetone-*d*_6_) δ 7.98 (d, *J_6′,2_* = 2.0 Hz, 1H, H-6′), 7.91 (br dd, *J*_2″,3_ = *J*_6″,5_ = 8.7 Hz, *J*_2″,F_ = *J*_6″,F_ = 5.5 Hz, 2H, H-2″, H-6″), 7.65 (d, *J*_2*′*,6_ = 2.0 Hz, 1H, H-2′), 7.35 (br t, *J*_3″,2_ = *J*_6″,5_ = 8.7 Hz, *J*_3″,F_ = *J*_5″,F_ = 8.7 Hz, 2H, H-3″, H-5″); ^19^F NMR (250 MHz, acetone-*d*_6_) δ −105.17 (F-4″); ^13^C NMR (90.5 MHz, acetone-*d*_6_) δ 192.5 (C_1_), 166.2 (d, *J*_3″,F_ = 252.0 Hz, C_4″_), 148.6 (C_3*′*_), 147.7 (C_4*′*_), 135.1 (C_5*′*_), 134.5 (d, *J*_1″,F_ = 2.8 Hz, C_1″_), 133.4 (d, *J*_2″,F_ = *J*_6″,F_ = 9.1 Hz, C_2″_, C_6″_), 129.2 (C_1*′*_), 121.9 (C_2*′*_), 118.9 (C_6*′*_), 116.4 (d, *J*_3″,F_ = *J*_5″,F_ = 22.0 Hz, C_3″_,C_5″_). IR (ATR) ν 3194, 2923, 1651, 1585, 1296 cm^–1^. HRMS (ESI−): calcd for [C_13_H_8_FNO_5_-H]^−^ 276.0308 ([M – H]^−^); found 276.0315.

#### (3,5-Dichlorophenyl)-(3,4-dihydroxy-5-nitrophenyl)methanone, **M-20**

Compound **M-20** was prepared as described for **M-14** by using **5c** (45 mg, 0.13 mmol), AlCl_3_ (35 mg, 0.26 mmol), and pyridine (50 μL, 0.39 mmol) in dry CHCl_3_ (4 mL). Purification by recrystallization from acetone and water provided **M-20** (35 mg, 0.10 mmol, 81% yield) as a green light solid. Mp 182–184 °C (from acetone). ^1^H NMR (250 MHz, acetone-*d*_6_) δ 10.70 (br s, 1H, −OH), 8.02 (d, *J*_6*′*,2′_ = 2.0 Hz, 1H, H-2′), 7.79 (t, *J*_4″,2″_ = *J*_4″,6″_ = 1.9 Hz, 1H, H-4″), 7.74 (d, *J*_2″,4″_ = *J*_2″,4″_ = 1.9 Hz, 2H, H-2″, H-6″), 7.66 (d, *J*_2*′*,6′_ = 2.0 Hz, H-2′); ^13^C NMR (100.6 MHz, acetone-*d*_6_) δ 191.3 (C_1_), 148.9 (C_3*′*_), 148.4 (C_4*′*_), 141.2 (C_5*′*_), 135.9 (C_3″,_ C_5″_), 135.3 (C_1*′*_), 132.6 (C_4″_), 128.7 (C_2″,_ C_6″_), 128.2 (C_1″_), 121.7 (C_2*′*_), 119.3 (C_6*′*_). IR (ATR) ν 3331, 1617, 1544, 1308 cm^–1^. HRMS (ESI−) calcd for [C_13_H_7_Cl_2_NO_5_-H]^−^ ([M – H]^−^) 325.9623, found 325.9630.

#### (3,5-Difluorophenyl)-(3,4-dihydroxy-5-nitrophenyl)methanone, **M-21**

Compound **M-21** was prepared as described for **M-14** by using **5d** (100 mg, 0.32 mmol), AlCl_3_ (86 mg, 0.65 mmol), and pyridine (105 μL, 1.29 mmol) in dry CHCl_3_ (10 mL). Purification by recrystallization from acetone and water furnished **M-21** (71 mg, 0.24 mmol, 75% yield) as a yellow light solid. Mp 135–137 °C (from acetone). ^1^H NMR (360 MHz, DMSO-*d*_6_) δ 10.96 (br s, 2H, 2 × −OH), 7.70 (d, *J_6′,2′_* = 2.0 Hz, 1H, H-6′), 7.60 (br tt, *J*_4″,2″_ = *J*_4″,6″_ = 2.2 Hz, *J*_4″,F_ = 9.3 Hz, 1H, H-4″), 7.48 (d, *J_2′,6′_* = 2.0 Hz, 1H, H-2′), 7.43 (m, *J*_2″,F_ = *J*_2″,F_ = 5.8 Hz, 2H, H-2″, H-6″); ^19^F NMR (250 MHz, DMSO-*d*_6_) δ −110.07 (s, F-3″, F-5″); ^13^C NMR (90.5 MHz, DMSO-*d*_6_) δ 190.7 (C_1_), 162.0 (dd, *J*_3″,F_ = *J*_5″,F_ = 12.6 Hz, *J*_3″,F_ = *J*_5″,F_ = 249.9 Hz, C_3″_,C_5″_), 147.9 (C_3*′*_), 146.6 (C_4*′*_), 140.4 (t, *J*_1″,F_ = 8.0 Hz, C_1″_), 136.9 (C_5*′*_), 125.4 (C_1*′*_), 118.5 (C_6*′*_), 118.5 (C_2*′*_), 112.5 (m, *J*_2″,F_ = *J*_6″,F_ = 18.5 Hz, C_2″,_ C_6″_), 107.7 (t, *J*_4″,F_ = 25.8 Hz, C_4″_). IR (ATR) ν 3363, 1617, 1587, 1436, 1323 cm^–1^. HRMS (ESI−) calcd for [C_13_H_7_F_2_NO_5_-H]^−^ ([M – H]^−^) 294.0214, found 294.0221.

#### (3,4-Dihydroxy-5-nitrophenyl)-(3-fluoro-5-hydroxyphenyl)methanone, **M-23**

Compound **M-23** was prepared as described for **M-14** by using **5e** (158 mg, 0.52 mmol), AlCl_3_ (511 mg, 3.80 mmol), and pyridine (360 μL, 4.45 mmol) in dry CHCl_3_ (10 mL). Purification by flash column chromatography (CH_2_Cl_2_/MeOH, 19:1) afforded catechol **M-23** (69 mg, 0.24 mmol, 46% yield) as a yellow solid. Mp 215–217 °C (from acetone). ^1^H NMR (360 MHz, acetone-*d*_6_) δ 10.61 (br s, 1H, −OH), 9.28 (br s, 2H, 2 × −OH), 8.02 (d, *J_6′,2′_* = 2.0 Hz, 1H, H-6′), 7.65 (d, *J*_2*′*,6′_ = 2.0 Hz, 1H, H-2′), 7.08 (s, C-6″), 7.00 (br dt, *J*_2″,F_ = 8.8 Hz, 1H, H-2″), 6.90 (br dt, *J*_4″,2″_ = 2.2 Hz, *J*_4″,F_ = 10.3 Hz, 1H, H-4″); ^19^F NMR (250 MHz, acetone-*d*_6_) δ −112.87 (t, *J* = 9.5 Hz, F-3″); ^13^C NMR (90.5 MHz, acetone-*d*_6_) δ 192.4 (d, *J*_CO,F_ = 2.7 Hz, C_1_), 164.1 (d, *J*_3″,F_ = 254.0 Hz, C_3″_), 159.9 (d, *J*_5″,F_ = 11.7 Hz, C_5″_), 148.8 (C_3*′*_), 148.1 (C_4*′*_), 140.6 (d, *J*_1″,F_ = 8.4 Hz, C_1″_), 135.2 (C_5*′*_), 128.7 (C_1*′*_), 121.8 (C_2*′*_), 119.1 (C_6*′*_), 113.5 (d, *J*_6″,F_ = 2.2 Hz, C_6″_), 108.1 (d, *J*_2″,F_ = 23.3 Hz, C_2″_), 107.4 (d, *J*_4″,F_ = 24.2 Hz, C_4″_). IR (ATR) ν 3229, 1599, 1442, 1252 cm^–1^. HRMS (ESI−) calcd for [C_13_H_8_FNO_6_-H]^−^ ([M – H]^−^) 292.0257, found 292.0265.

### Isothermal Titration Calorimetry

The thermodynamic parameters that characterize the binding of TTR ligands to WT-TTR were determined using a MicroCal Auto-iTC200 Calorimeter (MicroCal, Malvern-Panalytical), as detailed before.^44^ A 100 μM solution of the compound (in a PBS buffer pH 7.0 containing 100 mM KCl, 1 mM EDTA and 2.5% DMSO) was titrated into an ITC cell containing a 5 μM solution of WT-TTR in the same buffer at 25 °C. A stirring speed of 750 rpm and 2 μL injections were programmed, with a 150 s equilibration period between each injection to allow the calorimetric signal (thermal power) to return to baseline and a 10 μcal/s reference power. Two independent titrations were done for each TTR ligand. The experimental data were analyzed with a general model for a protein with two ligand-binding sites^93,94^ implemented in Origin 7.0 (OriginLab) accounting for cooperative and noncooperative binding. The best fit of the binding isotherm was attained with a model considering two identical binding sites (i.e., no cooperativity) for tolcapone and its derivatives.

### Urea-Induced TTR Tetramer Dissociation Kinetics

TTR solutions (1.8 μM in PBS) were incubated with the different TTR ligands (3.6 μM) for 30 min at RT, and 6 M urea was added. A control sample containing the same amount of DMSO rather than the compound was prepared. The process of unfolding was tracked by intrinsic fluorescence spectroscopy using an FP-8200 Spectrofluorometer (Jasco). Trp residues were excited at 295 nm, and emission spectra was collected from 310 to 400 nm. Trp exposure upon denaturation red shifts the wavelength of maximum fluorescence from 335 to 355 nm, approximately. The 355/335 fluorescence emission intensity was normalized from minimum (100% folded) to maximum (0% folded) and plotted as a function of time. The TTR fluorescence of the control sample after incubation at RT for 96 h in 6 M urea was considered the maximum.

### TTR *In Vitro* Aggregation Assay

The anti-aggregational activity of TTR ligands was evaluated using the established acid-mediated aggregation assay.^44^ In short, WT-TTR solutions (7 μM in 10 mM sodium phosphate, 100 mM KCl, 1 mM EDTA, 1 mM DTT, pH 7.0) were mixed with increasing concentrations of test compounds (prepared in 100% DMSO). The percentage of DMSO was adjusted to 5% (v/v) in the final reaction assay mixture. After incubating for 30 min at 37 °C, the pH of the samples was dropped to 4.2 by the addition of acetate buffer (100 mM sodium acetate, 100 mM KCl, 1 mM EDTA, 1 mM DTT, pH 4.2), and the solutions were further incubated for 72 h at 37 °C. The extent of TTR aggregation was assessed by measuring turbidity at 340 nm using a Varian Cary Eclipse Fluorescence Spectrophotometer (Agilent Technologies). As some of the compounds present dose-dependent absorbance at 340 nm, each measurement was corrected with a buffer containing the same concentration of the test compound but lacking TTR. For each inhibitor concentration, the percentage of TTR aggregation was given by the ratio of the turbidity of the sample of interest to that of a control sample incubated without compound multiplied by 100%.

### TTR Stabilization Studies by Isoelectric Focusing

Isoelectric focusing (IEF) under semidenaturing conditions was performed as described previously^95^ to evaluate the stabilizing effect of both tolcapone and **M-23** on recombinant TTR and in plasma TTR. Recombinant WT-TTR was produced using an *Escherichia coli* bacterial expression system, as detailed elsewhere.^96^ For the plasma assays, human plasma from control individuals (*n* = 6), carrying WT-TTR (≈3.9 μM), was incubated ON at 4 °C with tafamidis, tolcapone, or **M-23** at two different concentrations (19.5 and 39 μM). Similarly, recombinant WT-TTR (6 μM) was also treated ON at 4 °C with the same compounds at concentrations of 30 and 60 μM. DMSO (5%) was used as vehicle. Control samples were incubated in similar conditions without the compounds. After incubation, the samples were loaded into a native PAGE, and the gel band containing TTR was excised and applied to an IEF gel. The IEF gel contained 4 M urea (semidenaturing conditions) and 5% (v/v) ampholytes, pH 4–6.5 (Sigma-Merck), and was run at 1200 V for 5 h. Proteins were fixed and stained with Coomassie blue. The gels were scanned using a GS-900 calibrated densitometer (Bio-Rad) and analyzed by densitometry using the QuantityOne software version 4.6.6 (Bio-Rad). The ratio of the TTR tetramer over total TTR (TTR tetramer + monomer) was calculated for each plasma sample, and the percentage of tetramer stabilization was calculated as ((ratio treated sample – ratio control sample)/ratio control sample) × 100. Treated and control plasma samples come from the same donor.

### T_4_ Binding Competition Assay

Binding competition assays with radioactive T_4_ were performed by incubation of 5 μL of human plasma samples of WT-TTR carriers (*n* = 4) with 1 μL of [^125^I]-T_4_ (specific radioactivity 1250 μCi/μg; concentration 320 μCi/mL; Perkin Elmer) in the presence of 39 μM compounds. The negative control was performed by adding the same percentage of DMSO in the samples. After 1 h incubation at rt, plasma proteins were fractionated by native PAGE,^97^ and the gels were dried and exposed to an autoradiography film. The films were scanned, and the intensity of the bands was determined by densitometry using Image Lab software version 5.2.1 (Bio-Rad). The ratio of T_4_ bound to TTR over total T_4_ (TBG + ALB + TTR) was calculated for each sample and normalized to the maximum value, which corresponds to the negative control sample.

### Solubility Measurements

The solubility tests were carried out by weighing **M-23** and tolcapone (solid samples). Then, distilled or deionized water was added under gentle agitation until total dissolution. All the experiments were performed in triplicate.

### Cytotoxicity Assay

Cytotoxicity analyses were performed for evaluating the potential **M-23** chemical toxicity to human cells. Tolcapone was used for comparison. Two cell lines, HeLa and HepG2 cells, were cultured in MEM ALPHA (Gibco) and 10% FBS at 37 °C in 5% CO_2_ humidified atmosphere. HeLa cells were seeded at 3500 cells/well and HepG2 cells at 4500 cells/well in 96-well plates and incubated with increasing concentrations of compound at a range of concentrations from 2 to 100 μM during 72 h at 37 °C. Controls were performed with the equivalent amount of DMSO relative to each concentration of compound diluted in MilliQ water. Then, cell viability was determined by adding 10 μL of the PrestoBlue reagent (Thermo Fisher Scientific), and after an incubation period of 15 min at 37 °C, the fluorescence intensity was collected using a 590/20 filter with an excitation wavelength of 535 in a Victor3 Multilabel Reader (PerkinElmer). Experiments were carried out in triplicate, and the percentage of cell viability for each well was calculated as (intensity sample – mean intensity blank)/(mean intensity control – mean intensity blank) × 100, where ″mean intensity blank″ corresponds to the mean intensity of wells with PrestoBlue alone and ″mean intensity control″ is the mean intensity of wells that contain the corresponding percentage of DMSO.

### Crystallization and Structure Determination of the WT-TTR/M-23 Complex

Co-crystals of WT-TTR/M-23 were obtained as described previously.^44^ In short, WT-TTR (85 μM) was mixed with 10-fold molar excess of **M-23** and co-crystallized at 18 °C by the hanging-drop vapor diffusion method (1:1, complex and reservoir solution). Crystals were grown from a solution containing 30% PEG 400, 200 mM CaCl_2_, and 100 mM HEPES (pH 8.0) and directly flash-frozen in liquid nitrogen before analysis. Diffraction data were recorded from PEG400 cryo-cooled crystals (100 K) at the ALBA Synchrotron in Barcelona (BL13-XALOC beamline).^98^ Data were integrated and merged using XDS^99^ and scaled, reduced, and further analyzed using Ccp4.^100^ The structure of TTR/M-23 complex was determined by molecular replacement with Phenix (version 1.19.2-4158)^101^ using the crystal structure of TTR (PDB code 1F41) as a search model. Refinement was performed with Phenix, and model building was performed with Coot.^102^ Refinement and data statistics are provided in Table S5. Figures were prepared with Pymol (The PyMOL Molecular Graphics System, Version 2.0, Schrödinger, LLC).

### Statistical Analysis

All the graphs were generated with GraphPad Prism software version 6.0 (GraphPad Software, La Jolla, California, USA). Data are shown as means ± standard error of mean (SEM). The results obtained from TTR stabilization studies in human plasma were analyzed by one-way ANOVA Tukey’s test using GraphPad Prism 6.0. *p* < 0.05 was considered statistically significant (* statistically significant at *p* < 0.05; ** statistically significant at *p* < 0.01; *** statistically significant at *p* < 0.001).

## Supporting Information Available

Main structures together with H-bond evolution along the MD trajectories for all the systems; TTR tetramer stabilization effect of tafamidis, tolcapone, and **M-23** in human plasma as assessed by IEF; overlay of the WT-TTR binding pocket when bound to **M-23** and tolcapone; tolcapone–TTR binding poses from MD simulations in the absence of backbone constrains; ^1^H and ^13^C NMR spectra of all synthesized compounds; and the HPLC traces for the final compounds (PDF)
PDB coordinates for tolcapone (PDB)
PDB coordinates for **M-14** (PDB)
PDB coordinates for **M-17** (PDB)
PDB coordinates for **M-20** (PDB)
PDB coordinates for **M-21** (PDB)
PDB coordinates for **M-23** (PDB)
Molecular formula strings for the new compounds described in the study (CSV)

## Supplementary Material

jm2c01195_si_001.pdf

jm2c01195_si_002.pdb

jm2c01195_si_003.pdb

jm2c01195_si_004.pdb

jm2c01195_si_005.pdb

jm2c01195_si_006.pdb

jm2c01195_si_007.pdb

## Accession Codes

The atomic coordinates have been deposited into the Protein Data Bank with accession code 7QC5. The authors will release the atomic coordinates and experimental data upon article publication.

## ABBREVIATIONS

ATTR: transthyretin amyloidosis
TTR: transthyretin
T_4_: thyroxine
FAP: familial amyloid polyneuropathy
FAC: familial amyloid cardiomyopathy
SSA: senile systemic amyloidosis
HBP: halogen binding pocket
ITC: isothermal titration calorimetry
IEF: isoelectric focusing
