# Supplementary material for: Development of a Highly Potent Transthyretin Amyloidogenesis Inhibitor: Design, Synthesis, and Evaluation
Source: J Med Chem. 2022 Oct 28;65(21):14673–91. doi: 10.1021/acs.jmedchem.2c01195 (PMC9661476; doi:10.1021/acs.jmedchem.2c01195)
Supplement: Supplementary file 1 — jm2c01195_si_001.pdf [file jm2c01195_si_001.pdf]

Supporting Information

Development of a highly potent transthyretin amyloidogenesis inhibitor. Design, synthesis and evaluation.

Francisca Pinheiro[†], Irantzu Pallarès[†], Francesca Peccati[§], Adrià Sánchez-Morales[§], Nathalia Varejão[†], Filipa Bezerra[‡,‖], David Ortega-Alarcon[⊥,#], Danilo Gonzalez[§], Marcelo Osorio[§], Susanna Navarro[†], Adrián Velázquez-Campoy[⊥,#], Maria Rosário Almeida[‡,‖], David Reverter[†], Félix Busqué[§], Ramon Alibés[§], Mariona Sodupe[§] and Salvador Ventura[†,¶]*

[†] Institut de Biotecnologia i Biomedicina and Departament de Bioquímica i Biologia Molecular, Universitat Autònoma de Barcelona, Bellaterra, Barcelona 08193, Spain.
[§] Departament de Química, Universitat Autònoma de Barcelona, Bellaterra, Barcelona 08193, Spain.
[‡] Molecular Neurobiology Group, i3S – Instituto de Investigação e Inovação em Saúde, IBMC – Instituto de Biologia Molecular e Celular, Universidade do Porto, 4200-135 Porto, Portugal.
[‖] Departamento de Biologia Molecular, ICBAS – Instituto de Ciências Biomédicas Abel Salazar, Universidade do Porto, 4050-313 Porto, Portugal.
[⊥]Department of Biochemistry and Molecular & Cellular Biology, and Institute for Biocomputation and Physics of Complex Systems (BIFI), Joint Unit GBsC-CSIC-BIFI, Universidad de Zaragoza, 50018 Zaragoza (Spain).
[#] Aragon Institute for Health Research, 50009 Zaragoza (Spain) and Biomedical Research Network Center in Hepatic and Digestive Diseases (CIBERehd), 28029 Madrid (Spain).
[¶] ICREA, Passeig Lluis Companys 23, E-08010 Barcelona, Spain.

* Correspondence to: Salvador Ventura (salvador.ventura@uab.es)

Table of contents

Hydrogen bond contacts, frequency, and average and shortest H-bond distances for WT-TTR in complex with tolcapone and halogenated tolcapone analogues	S3
Hydrogen bond contacts, frequency, and average and shortest H-bond distances for WT-TTR in complex with demethylated 3,5-disubstituted and halogenated tolcapone analogues	S3
Ser117···Ser17 hydrogen bond contacts, frequency and average and shortest H-bond distances for WT-TTR in the absence or presence of tolcapone and its analogs	S4

Ser117···Ser117 interdimer average and shortest distances for WT-TTR in the absence or presence of tolcapone and its analogs	S5
Data collection and refinement statistics	S6
Tolcapone – TTR, **M-14** – TTR and **M-17** – TTR binding site and main contacts evolution along the 150 ns trajectory	S7
**M-20** – TTR, **M-21** – TTR and **M-23** – TTR binding site and main contacts evolution along the 150 ns trajectory	S8
TTR tetramer stabilization effect of tafamidis, tolcapone and **M-23** in human plasma as assessed by IEF	S10
Overlay of one of the WT-TTR T_4-binding sites when bound to **M-23** and tolcapone	S10
Tolcapone-TTR binding-poses from MD simulations in the absence of backbone constraints	S11
^{1}H and ^{13}C NMR spectra of new compounds	S12
HPLC traces for the final compounds	S34
References	S39

Table S1. Hydrogen bond contacts, frequency (in %), and average and shortest H-bond distances (in Å) for WT-TTR in complex with tolcapone and halogenated tolcapone analogues.

Compound	Ligand	L-TTR contacts	Frequency	Average distance	Shortest distance
Tolcapone	L1	C=O-THR119	10.7	2.83	2.63
**M-14**	L1	NO2-THR106	7.1	2.84	2.62
**M-14**	L2	C=O-LYS15	12.9	2.84	2.68
**M-17**	L1	C=O-THR119	35.7	2.82	2.63
**M-17**	L2	C=O-THR119	62.1	2.82	2.55
**M-17**	L1	F-SER117A	17.1	2.84	2.65
**M-17**	L1	F-THR119	33.6	2.86	2.68
**M-17**	L2	F-SER117B	23.6	2.88	2.68
**M-17**	L2	F-THR118	18.2	2.91	2.73

Table S2. Hydrogen bond contacts, frequency (in %), and average and shortest H-bond distances (in Å) for WT-TTR in complex with demethylated 3,5-disubstituted and halogenated tolcapone analogues.

Compound	Ligand	L-TTR Contacts	Frequency	Average distance	Shortest distance
**M-20**	L1	Cl-THR119	59.3	3.28	2.95
**M-20**	L2	Cl-THR119	44.3	3.28	2.93
**M-21**	L1	C=O-THR119	21.4	2.86	2.68
**M-21**	L2	C=O-THR119	12.2	2.85	2.63
**M-21**	L1	F-SER117A	7.1	2.88	2.69
**M-21**	L2	F-SER117B	17.9	2.86	2.64
**M-21**	L2	F-SER117D	9.3	2.90	2.79
**M-23**	L1	C=O-THR119	48.6	2.83	2.59
**M-23**	L2	C=O-THR119	37.1	2.84	2.63
**M-23**	L1	F-SER117A	87.1	2.81	2.59
**M-23**	L1	OH-SER117B	22.1	2.77	2.59
**M-23**	L2	OH-SER117A	90.7	2.79	2.52
**M-23**	L2	OH-SER117C	15.0	2.79	2.60

Table S3. Ser117···Ser17 hydrogen bond contacts, frequency (in %) and average and shortest H-bond distances (in Å) for WT-TTR in the absence or presence of tolcapone and its analogs.

Ligand	Hydrogen bond	Frequency	Average distance	Shortest distance
TTR	SER117A-SER117B	25	2.79	2.53
TTR	SER117C-SER117D	17	2.79	2.56
Tolcapone	SER117A-SER117B	86	2.77	2.54
Tolcapone	SER117C-SER117D	83	2.78	2.58
**M-14**	SER117A-SER117B	76	2.78	2.51
**M-14**	SER117C-SER117D	76	2.79	2.53
**M-17**	SER117A-SER117B	91	2.76	2.49
**M-17**	SER117C-SER117D	61	2.81	2.56
**M-20**	SER117A-SER117B	81	2.79	2.52
**M-20**	SER117C-SER117D	84	2.79	2.54
**M-21**	SER117A-SER117B	68	2.75	2.51
**M-21**	SER117C-SER117D	69	2.79	2.59
**M-23**	SER117A-SER117B	90	2.74	2.48
**M-23**	SER117C-SER117D	77	2.77	2.57

Table S4. Ser117···Ser117 interdimer average and shortest distances (in Å) for WT-TTR in the absence or presence of tolcapone and its analogs.

Ligand	Hydrogen bond	Average distance	Shortest distance
TTR	SER117A-SER117C	5.18	4.19
TTR	SER117B-SER117D	5.49	4.70
Tolcapone	SER117A-SER117C	4.85	4.15
Tolcapone	SER117B-SER117D	5.73	4.26
**M-14**	SER117A-SER117C	4.98	3.85
**M-14**	SER117B-SER117D	5.39	4.37
**M-17**	SER117A-SER117C	4.88	4.01
**M-17**	SER117B-SER117D	5.48	4.35
**M-20**	SER117A-SER117C	5.12	3.70
**M-20**	SER117B-SER117D	5.42	4.53
**M-21**	SER117A-SER117C	5.03	3.92
**M-21**	SER117B-SER117D	5.66	4.82
**M-23**	SER117A-SER117C	5.01	4.33
**M-23**	SER117B-SER117D	5.03	4.18

Table S5. Data collection and refinement statistics. Values in parentheses are for the last shell.

	**WT-TTR/M-23**
**Data collection**	
Beamline	ALBA-XALOC
Space group	$P2_12_12$
Wavelength (nm)	0.9791
Resolution range (Å)	38.89 – 1.20
a, b, c (Å)	84.37, 43.82, 65.47
α, β, γ (°)	α = β = γ = 90
Unique reflections	76,526
Data redundancy	5.4(5.4)
R_{merge}	0.038(0.65)
$CC_{1/2}$	0.999(0.83)
(I/σ(I))	17.8(2.4)
Completeness (%)	99.9(99.9)
**Structure refinement**	
Resolution range (Å)	38.89 – 1.20
Non-anomalous reflections	76,460
R_{work}/R_{free} (%)	16.0/17.3
Number of all atoms	2,144
RMSD bond (Å)/Angle (°)	0.012/1.254
**Ramachandran plot**	
Favored (%)	97.81
Allowed (%)	2.19
Disallowed (%)	0
Overall B factors (Å^2)	15.4
**PDB code**	7QC5

A

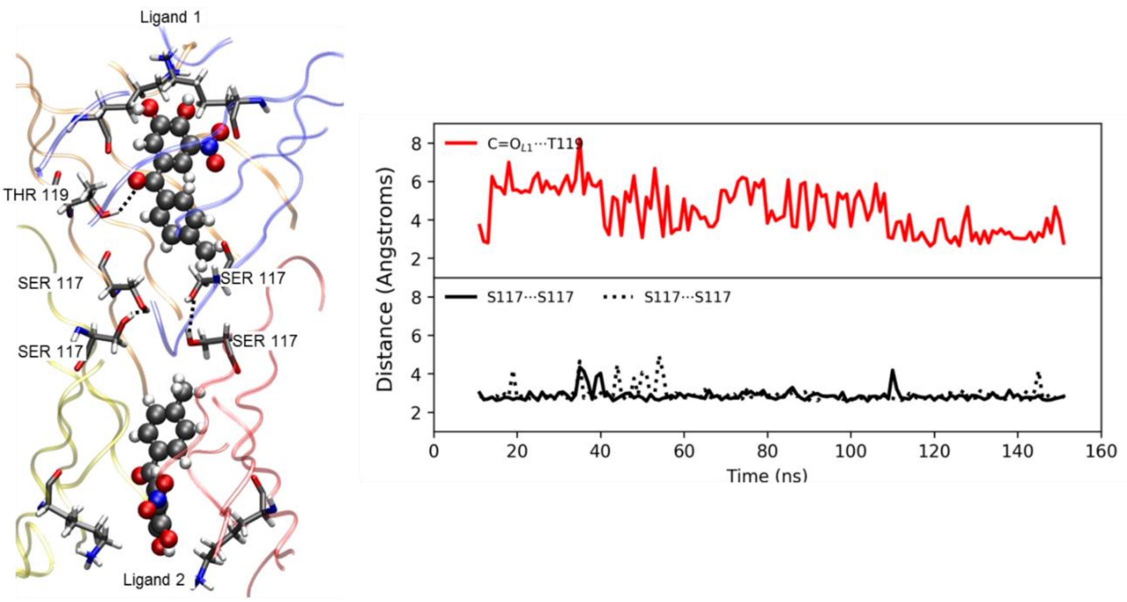

Ligand 1
THR 119
SER 117
SER 117
SER 117
SER 117
Ligand 2
C=O$_{L1}$···T119
S117···S117
S117···S117
Distance (Angstroms)
Time (ns)
8
6
4
2
0
20
40
60
80
100
120
140
160

B

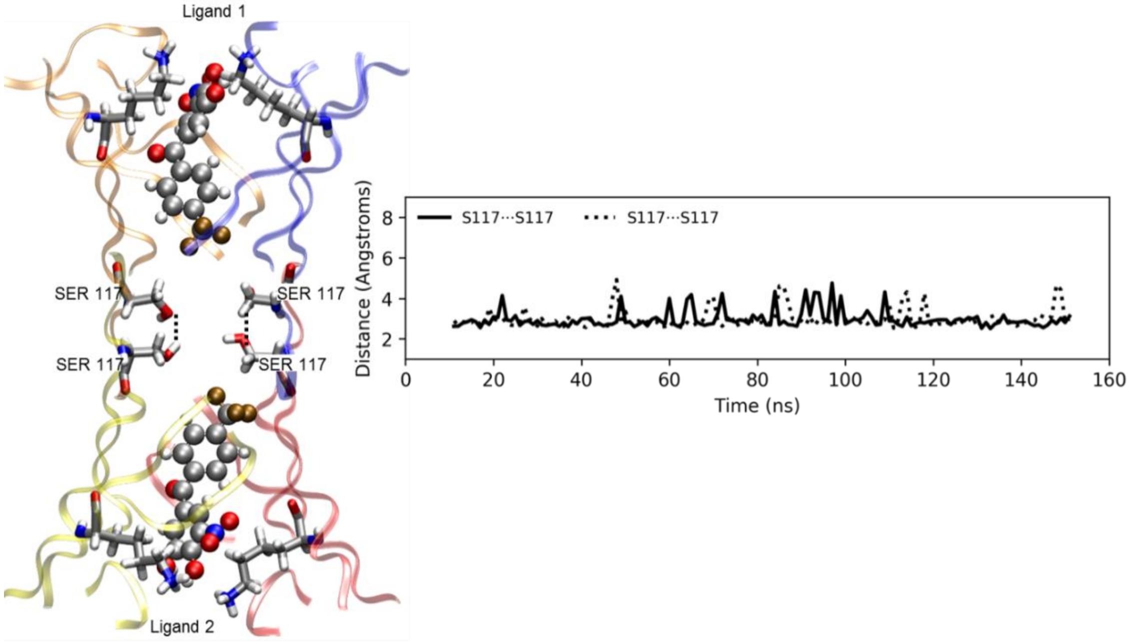

Ligand 1
SER 117
SER 117
SER 117
SER 117
Ligand 2
S117···S117
S117···S117
Distance (Angstroms)
Time (ns)
8
6
4
2
0
20
40
60
80
100
120
140
160

C

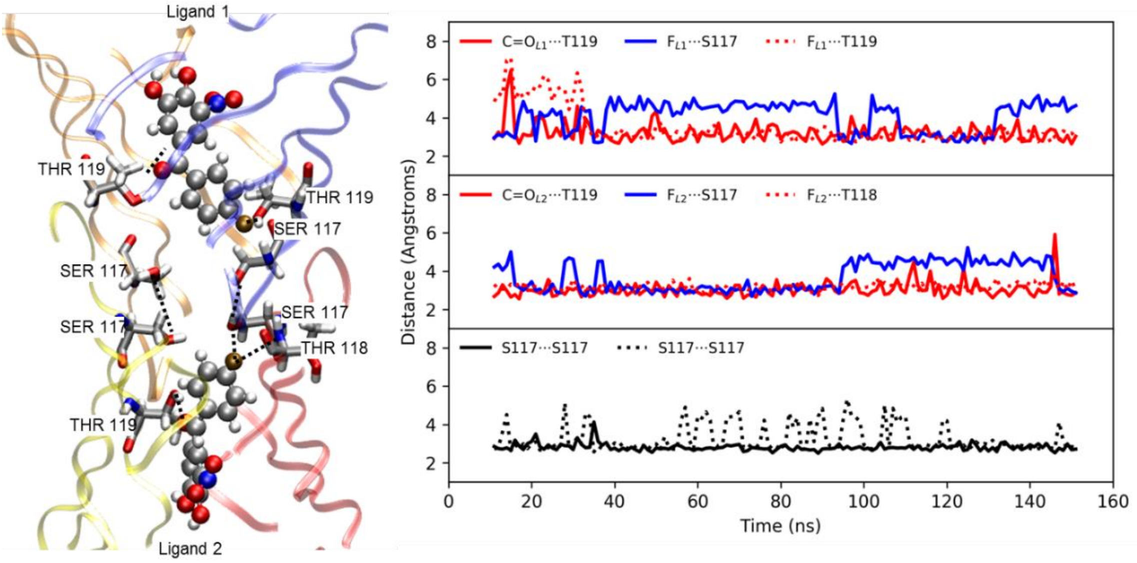

Figure S1. Tolcapone – TTR (A), **M-14** – TTR (B) and **M-17** – TTR (C) binding site and main contacts evolution along the 150 ns trajectory.

A

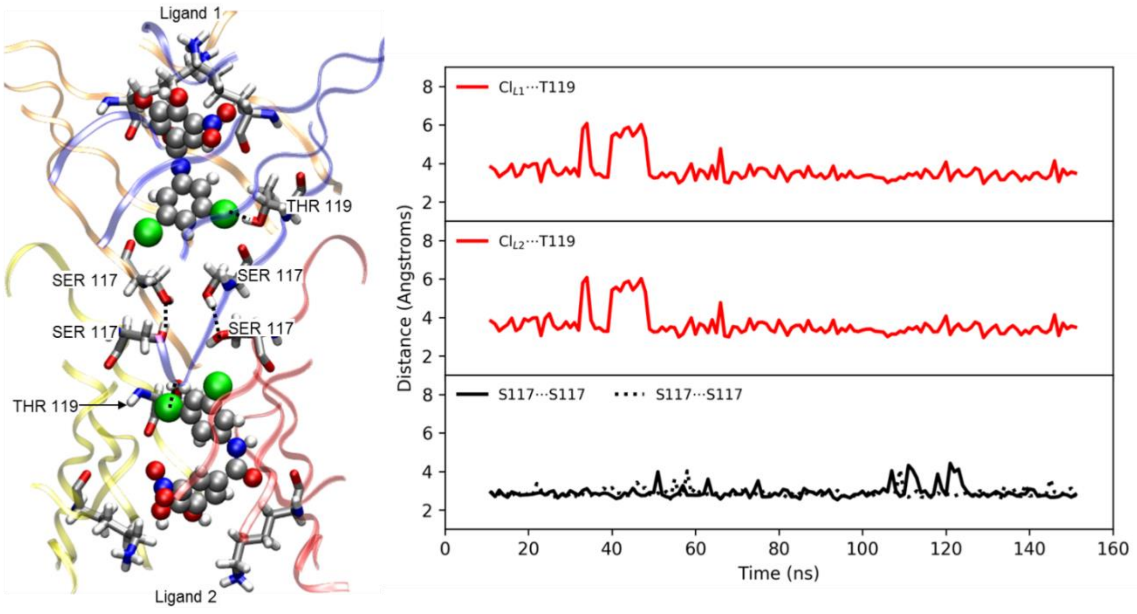

B

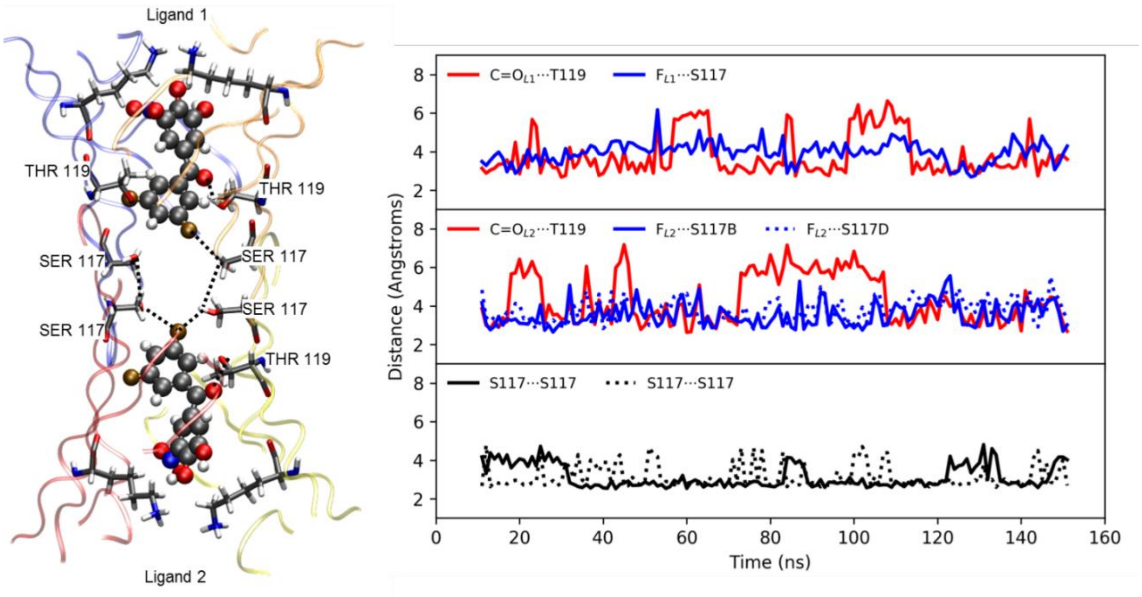

C

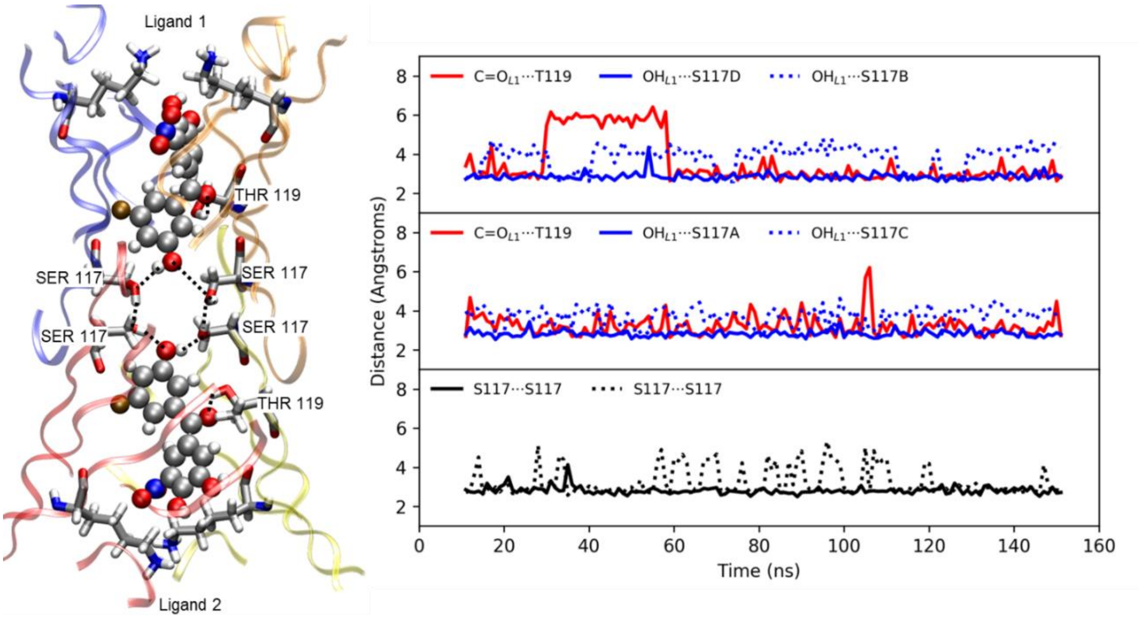

Figure S2. M-20 – TTR (A), **M-21** – TTR (B) and **M-23** – TTR (C) binding site and main contacts evolution along the 150 ns trajectory.

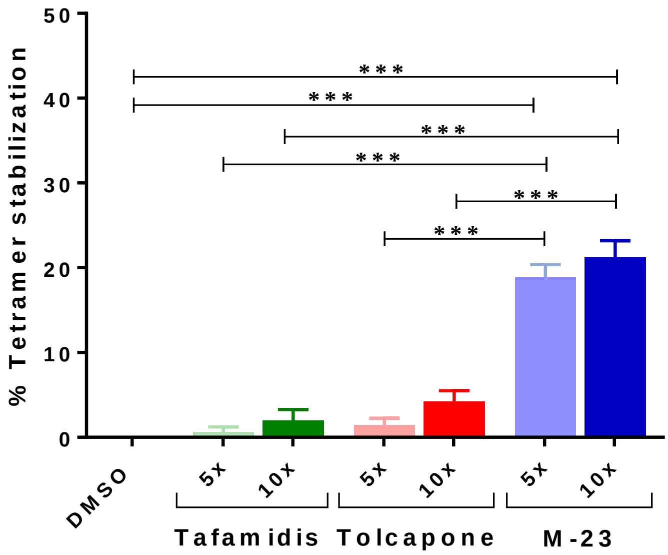

Figure S3. TTR tetramer stabilization effect of tafamidis, tolcapone and **M-23** in human plasma as assessed by IEF under semi-denaturing conditions. The test compounds were five (5x) or ten (10x) times more concentrated than WT-TTR. The values represent mean ± SEM (n=6); ***$p < 0.001$.

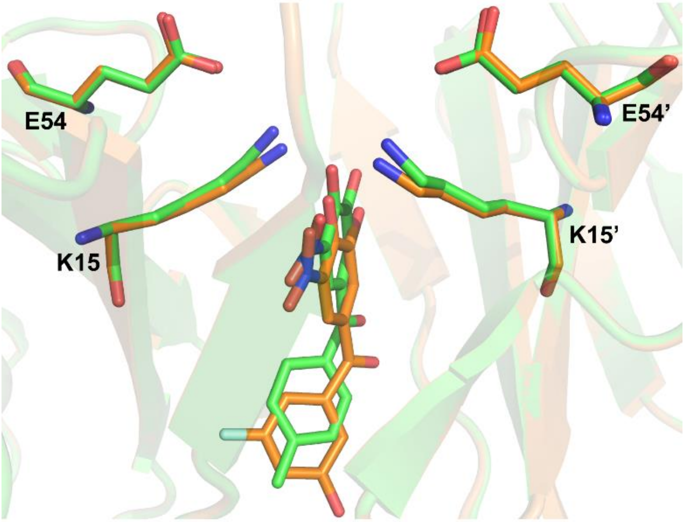

Figure S4. Overlay of one of the WT-TTR T_4-binding sites when bound to **M-23** (PDB: 7QC5) and tolcapone (PDB: 4D7B)[1], highlighting the inward movement of Lys15 from both chains when the protein is bound to **M-23**. WT-TTR/**M-23** and WT-TTR/tolcapone structures are colored in orange and green, respectively. Ligands and residues Lys15 and Glu54 are shown in sticks.

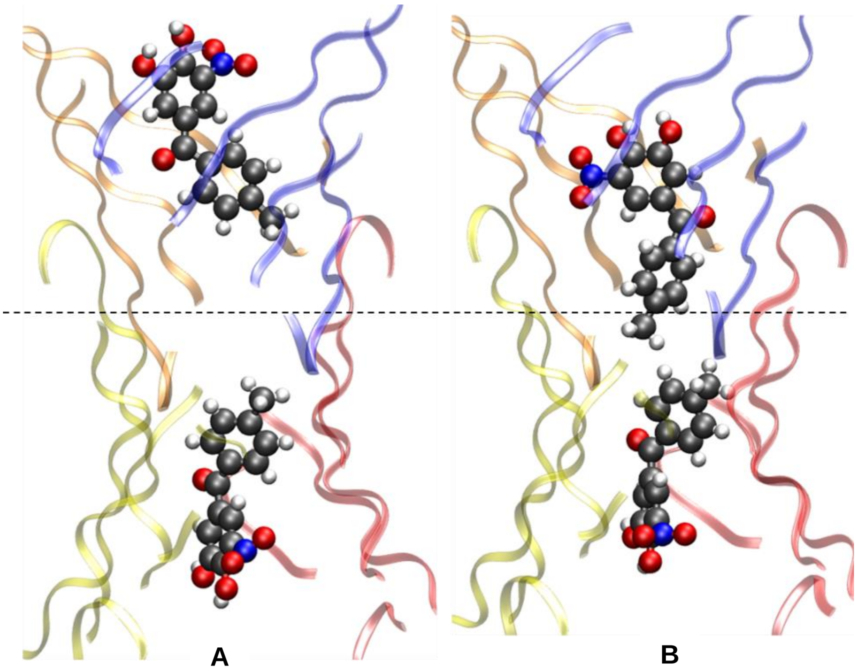

Figure S5. Tolcapone-TTR binding-poses from MD simulations in the absence of backbone constraints. Starting (A) and final (B) geometries are shown.

^{1}H and ^{13}C NMR spectra of new compounds

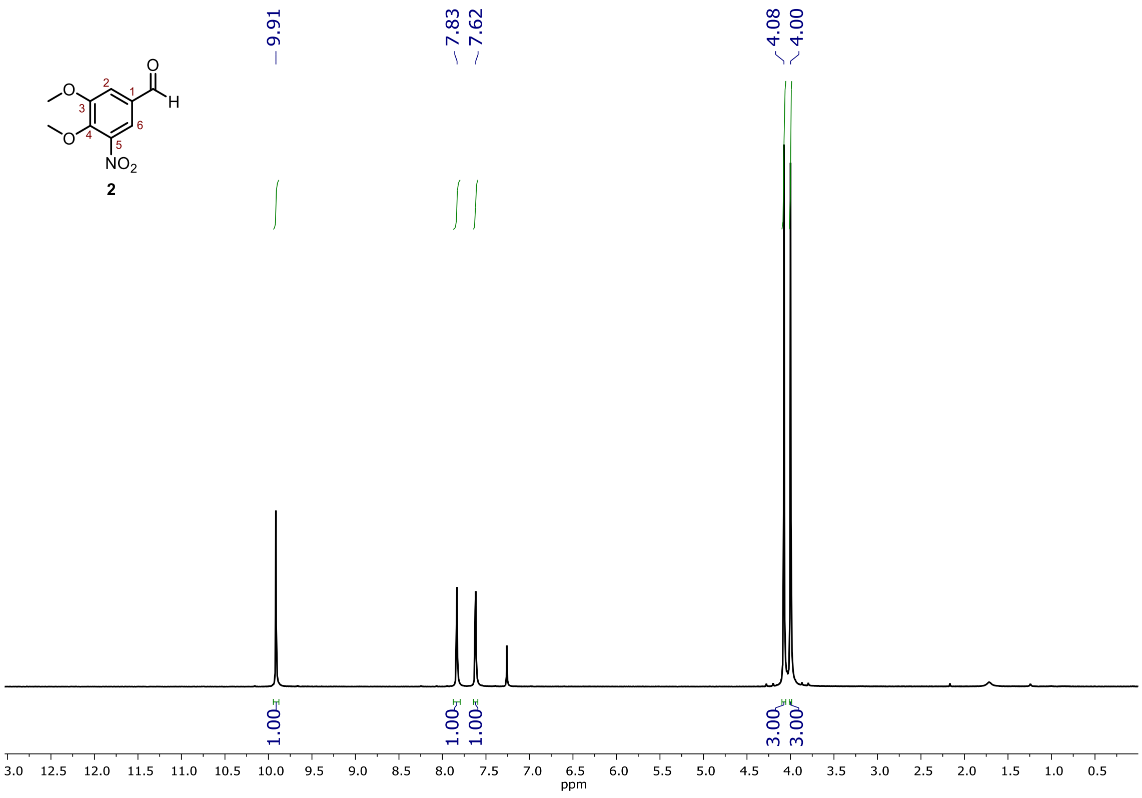

^{1}H NMR (250 MHz, $CDCl_3$)

Figure S6. ^{1}H NMR spectrum of compound **2**.

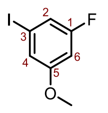

1e

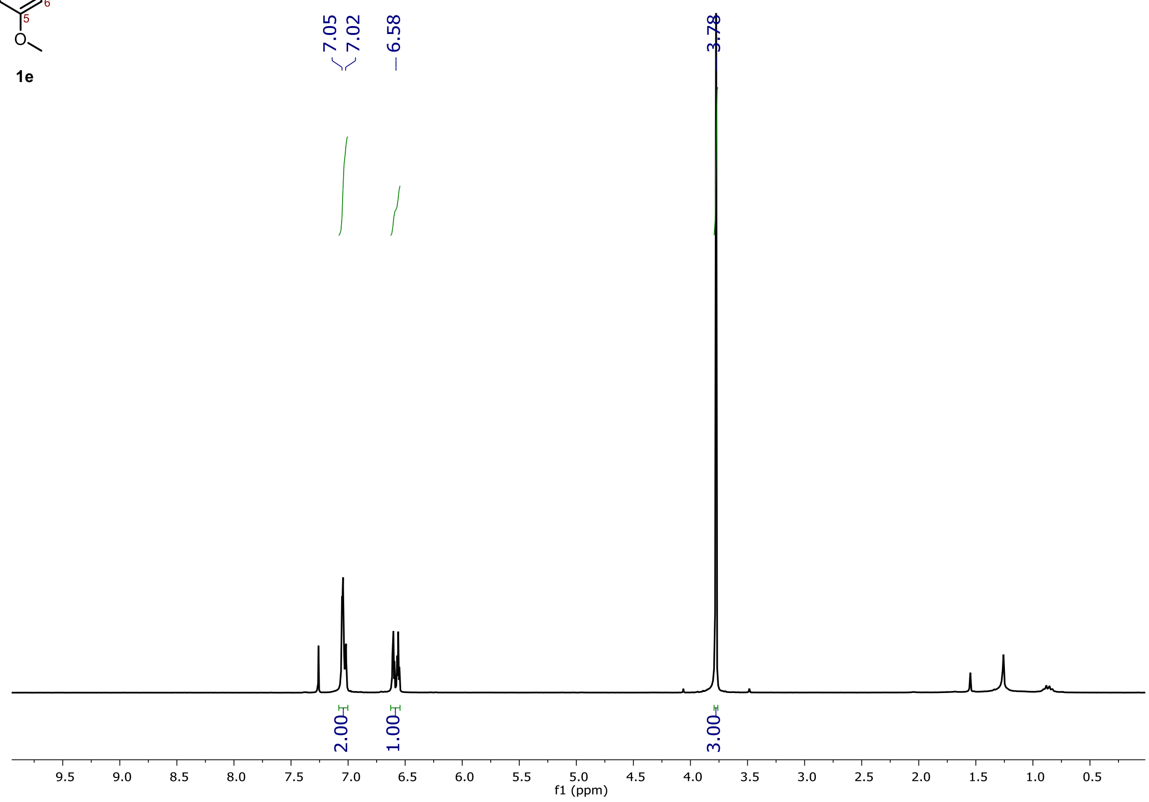

^{1}H NMR (400 MHz, $CDCl_3$)

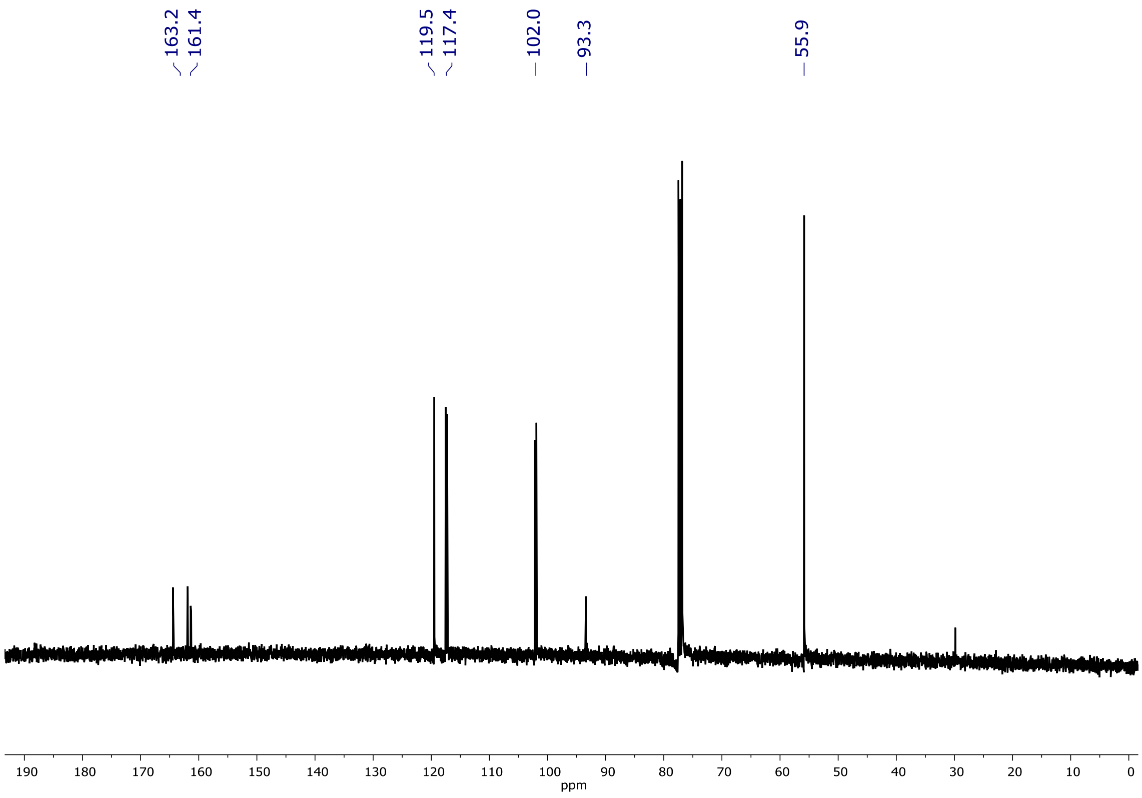

^{13}C NMR (100 MHz, $CDCl_3$)

Figure S7. ^{1}H and ^{13}C NMR spectra of compound **1e**.

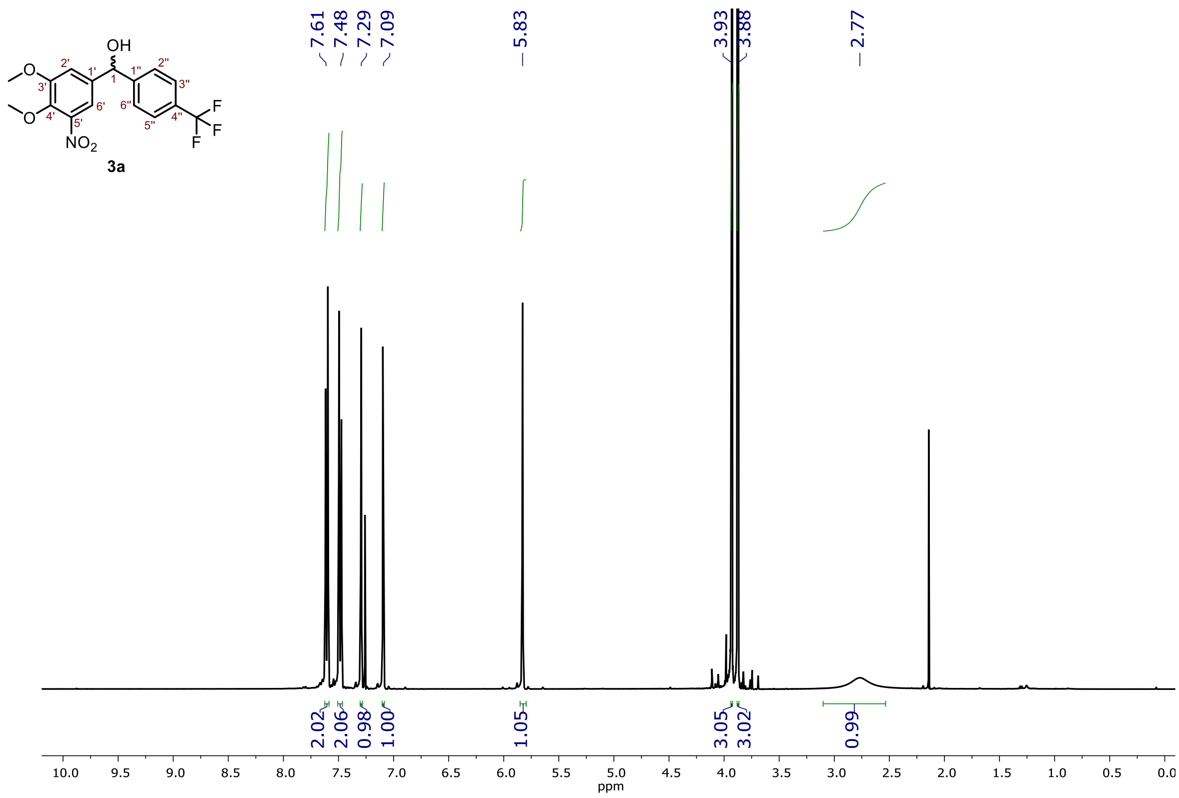

^{1}**H NMR** (400 MHz, $CDCl_3$)

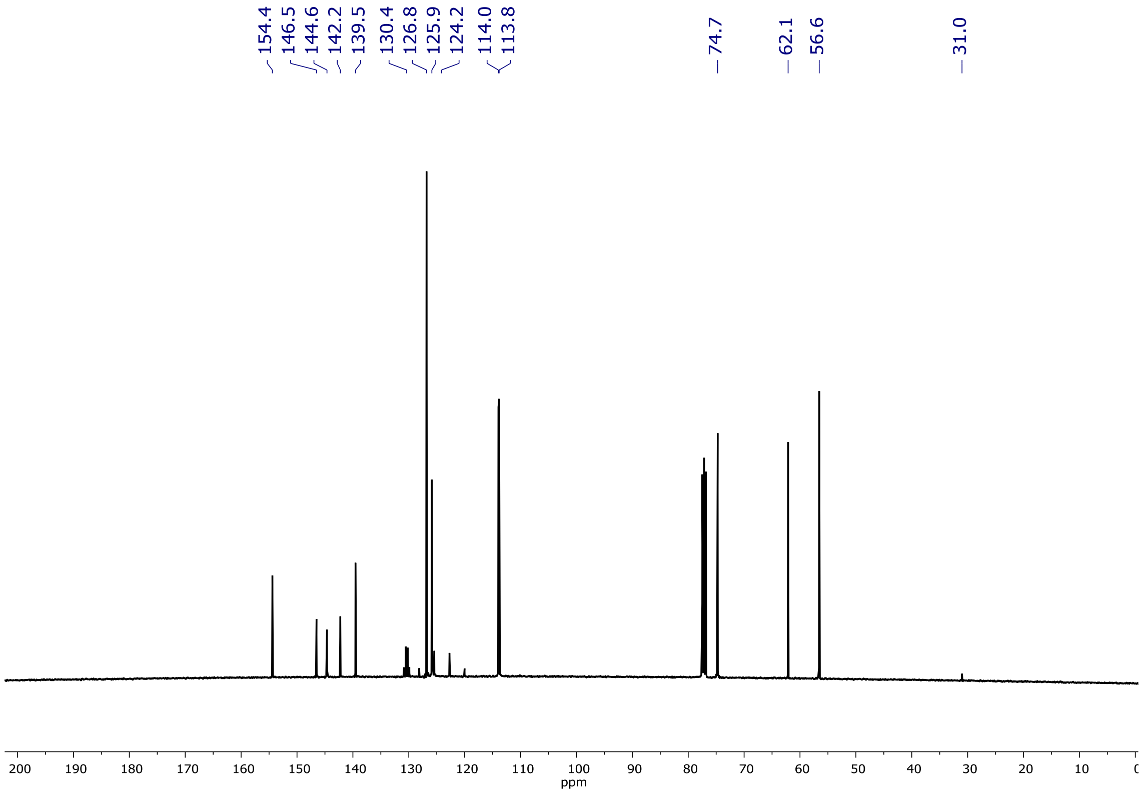

^{13}**C NMR** (100 MHz, $CDCl_3$)

Figure S8. ^{1}H and ^{13}C NMR spectra of compound **3a**.

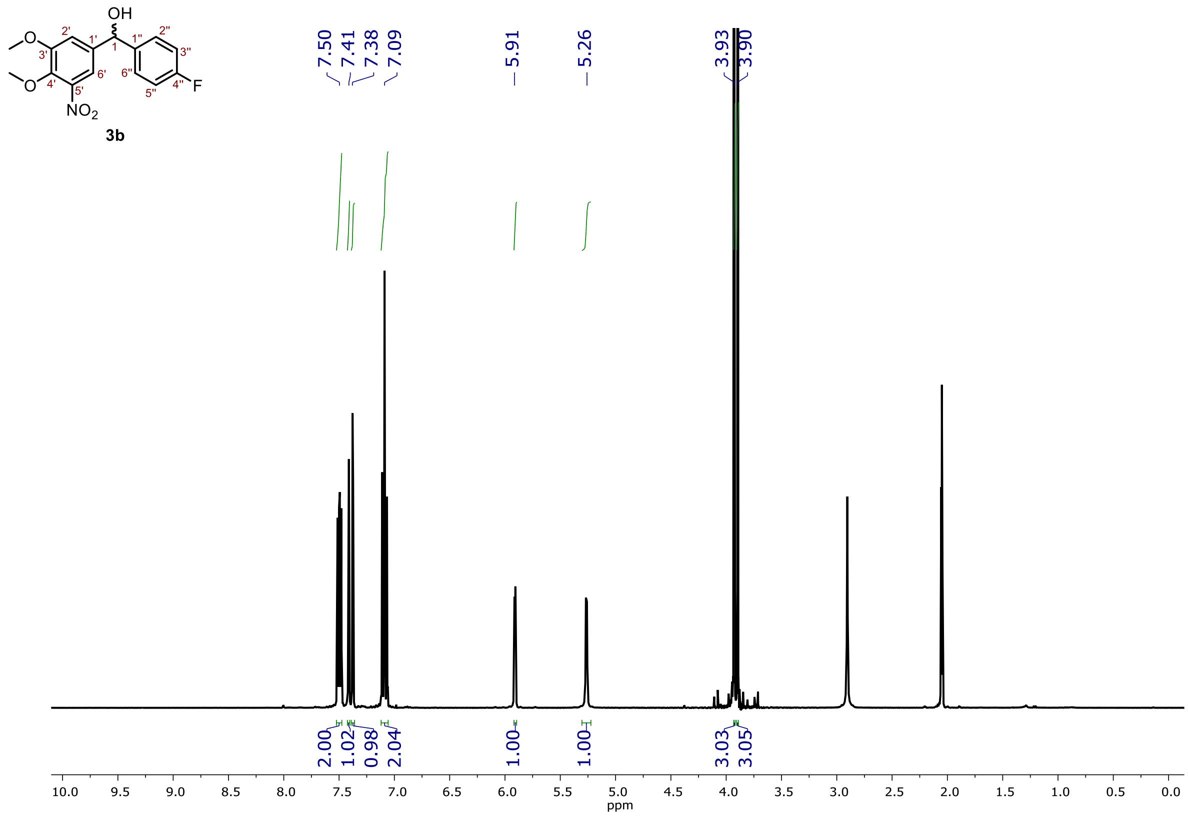

^{1}H NMR (400 MHz, acetone-d_6)

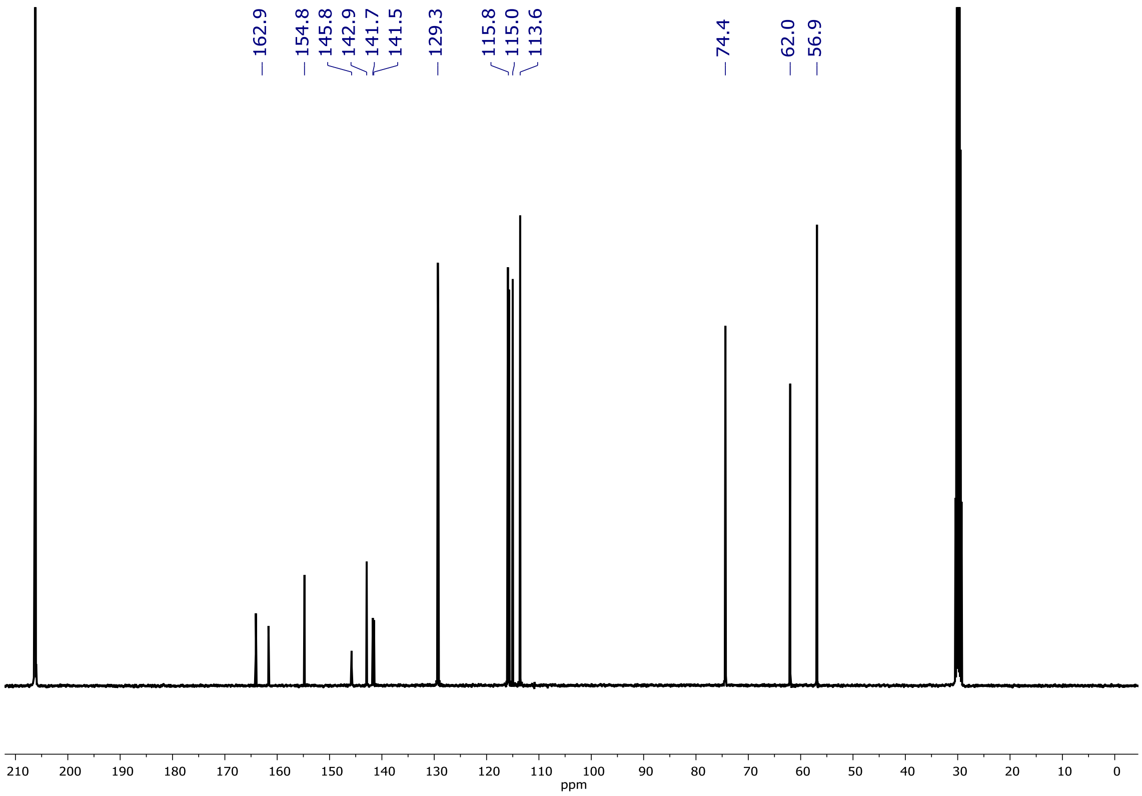

^{13}C NMR (100 MHz, acetone-d_6)

Figure S9. ^{1}H and ^{13}C NMR spectra of compound **3b**.

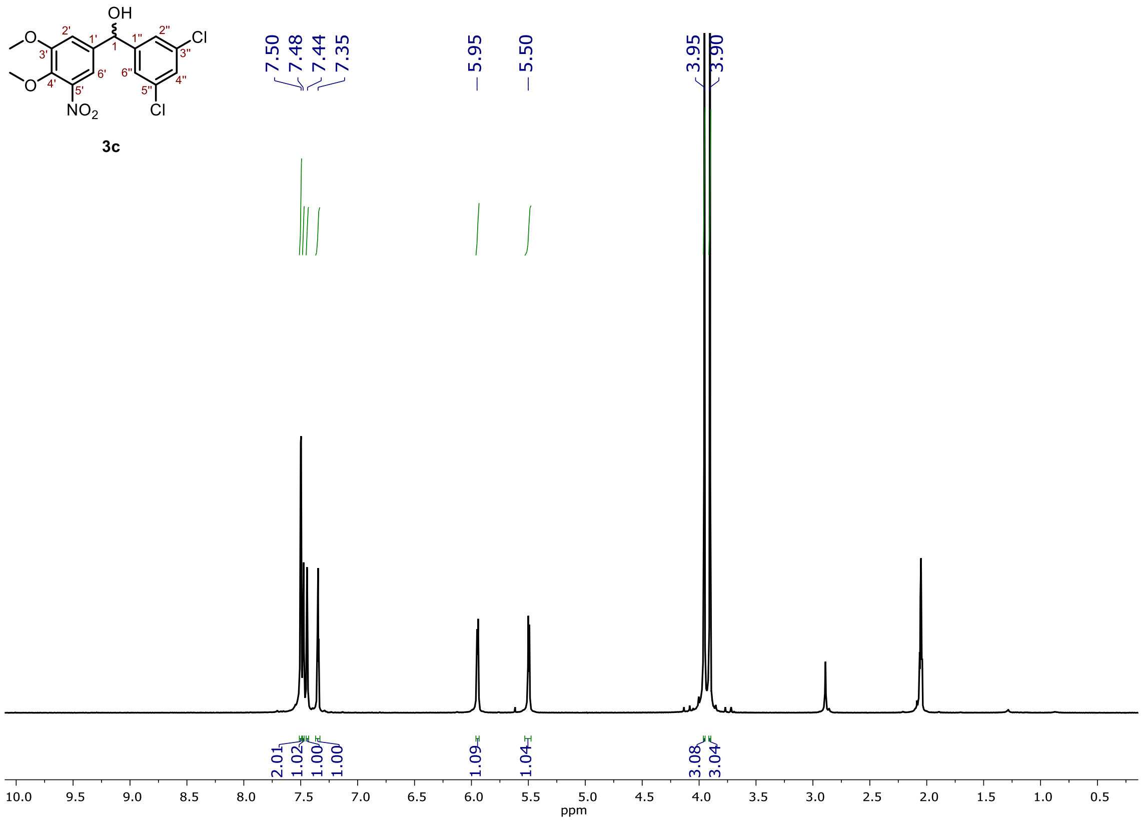

^{1}H NMR (400 MHz, acetone-d$_6$)

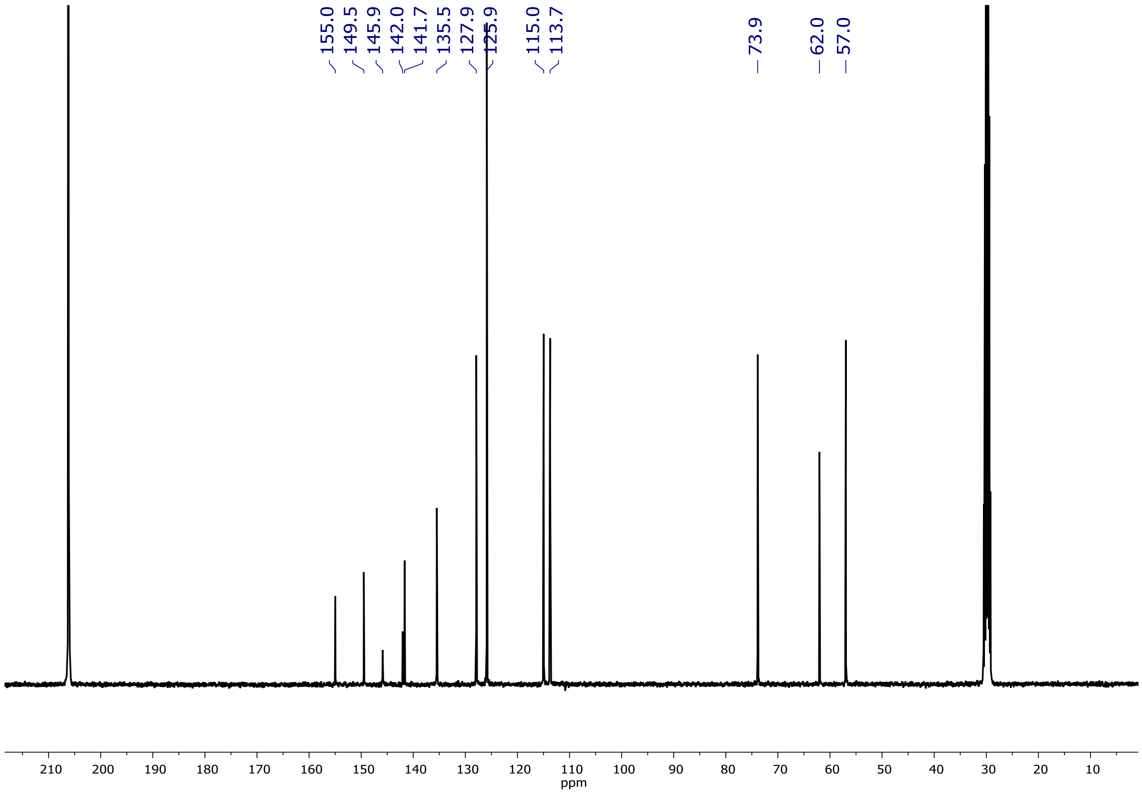

^{13}C NMR (90 MHz, acetone-d$_6$)

Figure S10. ^{1}H and ^{13}C NMR spectra of compound **3c**.

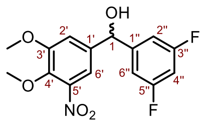

3d

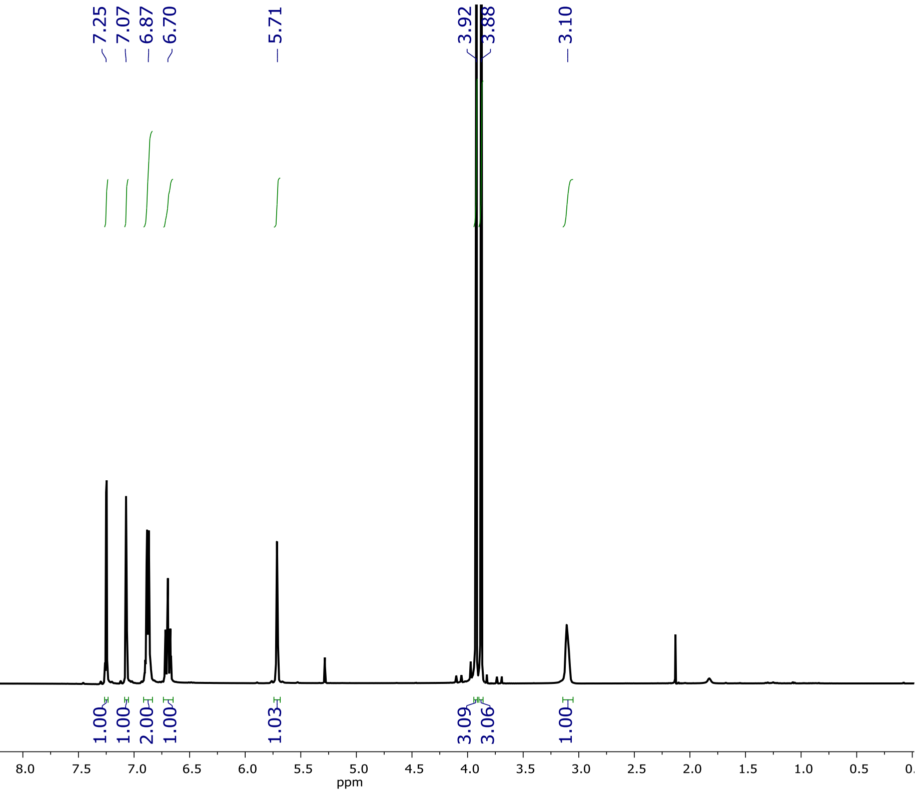

¹H NMR (400 MHz, $CDCl_3$)

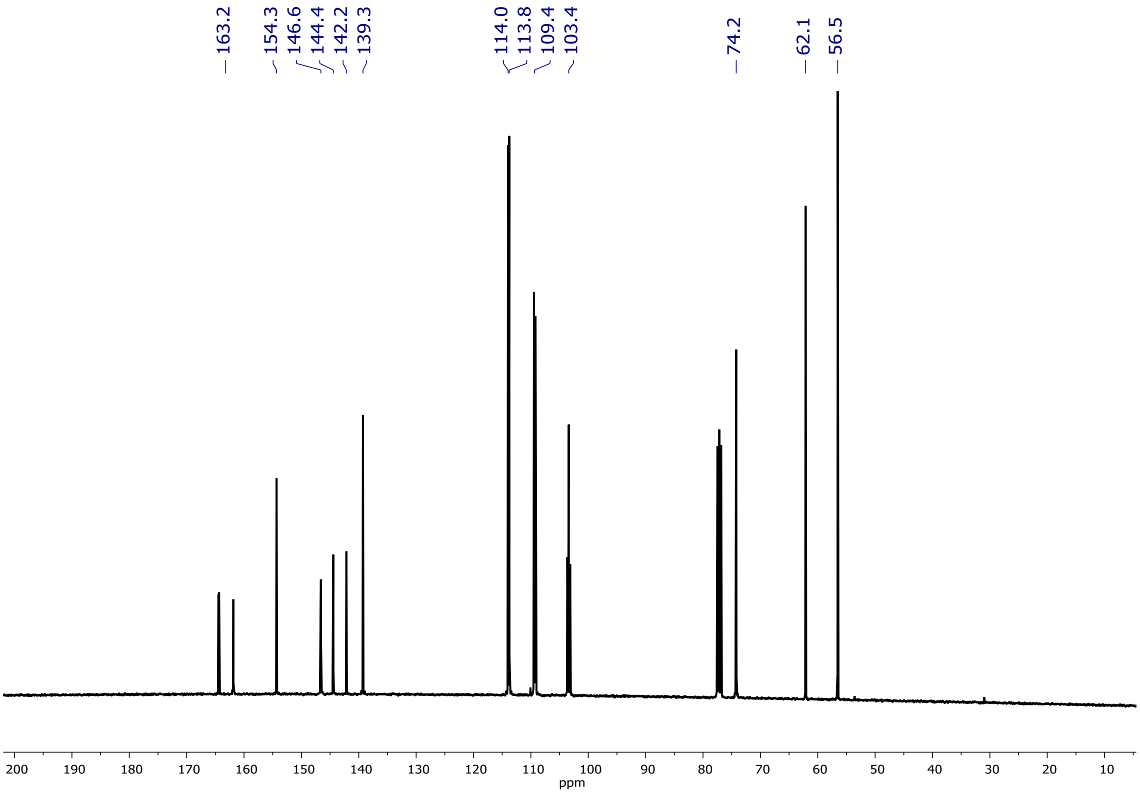

¹³C NMR (100 MHz, $CDCl_3$)

Figure S11. ^{1}H and ^{13}C NMR spectra of compound **3d**.

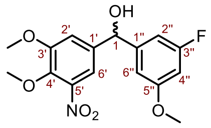

3e

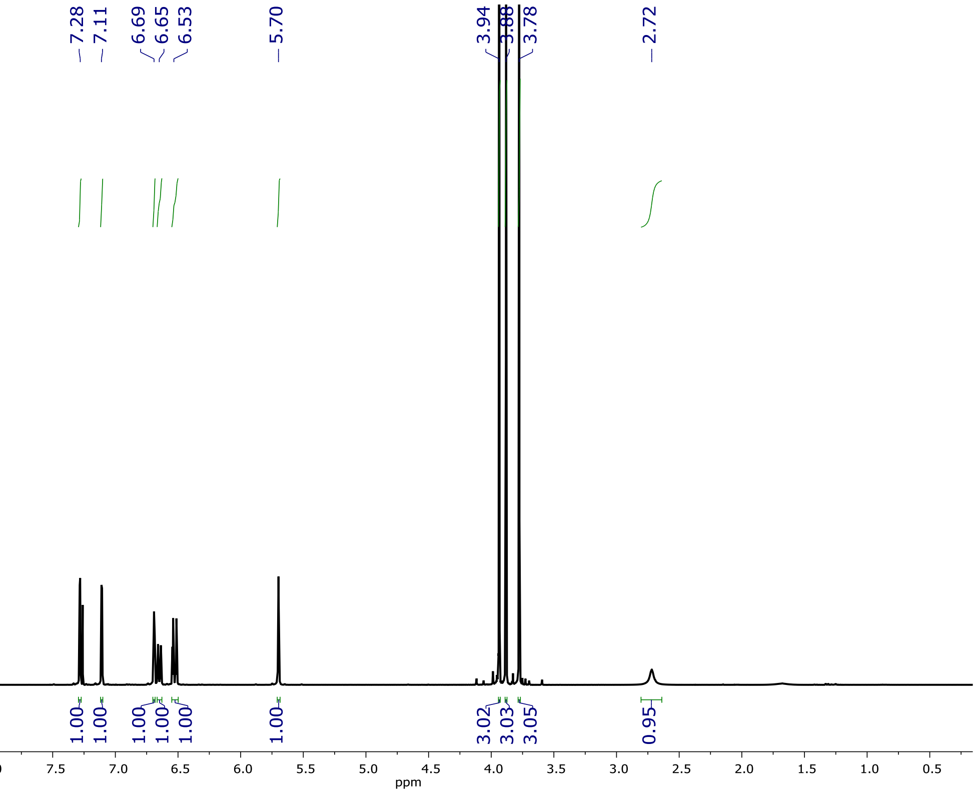

^{1}H NMR (400 MHz, $CDCl_3$)

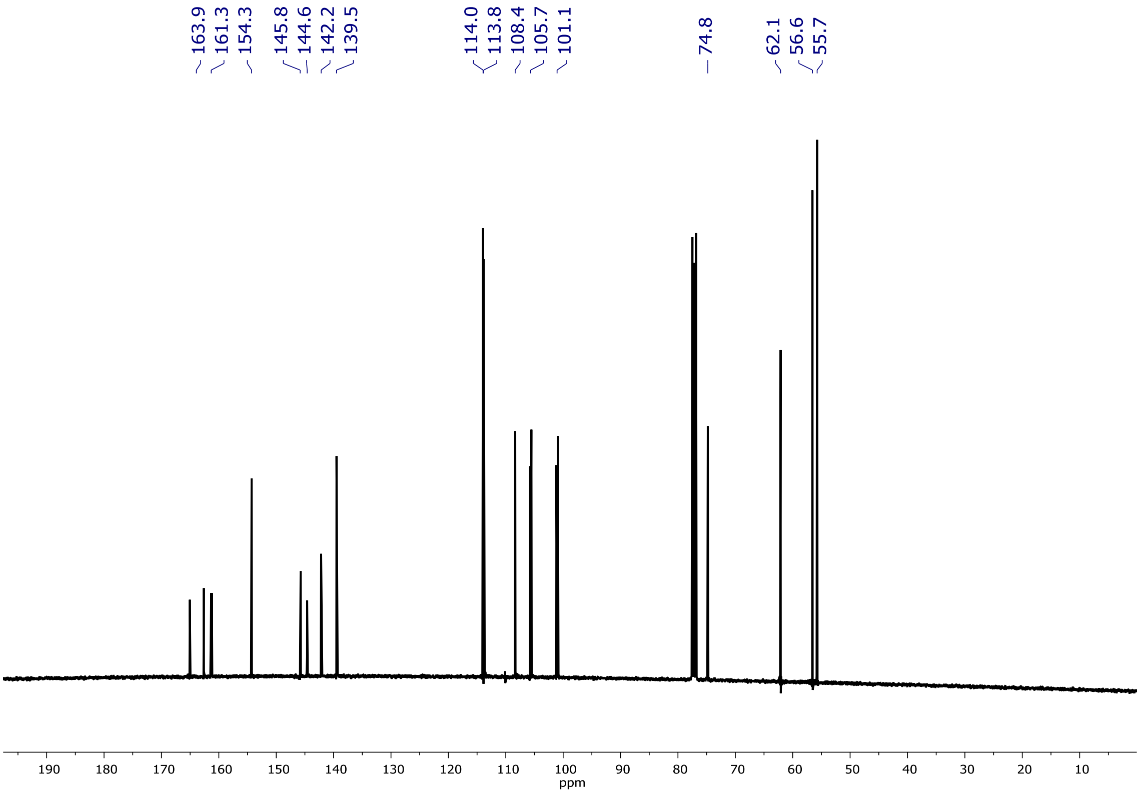

^{13}C NMR (100 MHz, $CDCl_3$)

Figure S12. ^{1}H and ^{13}C NMR spectra of compound **3e**.

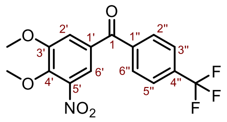

4a

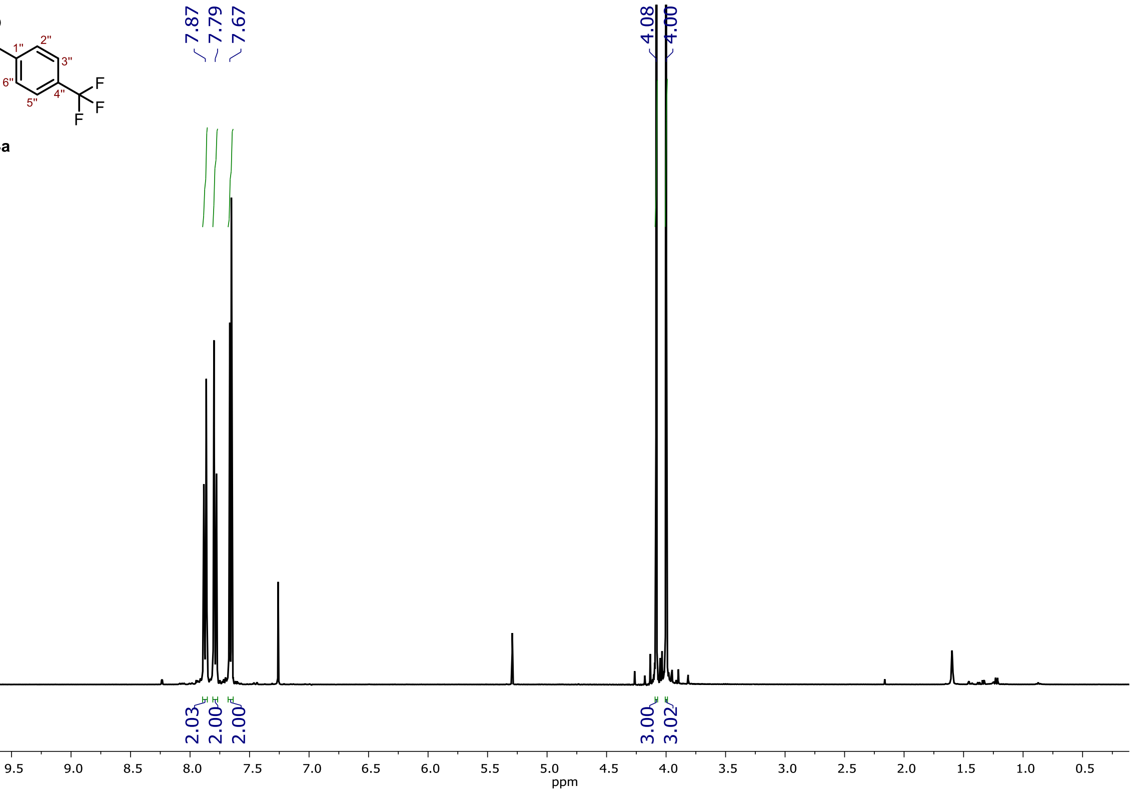

^{1}H NMR (400 MHz, $CDCl_3$)

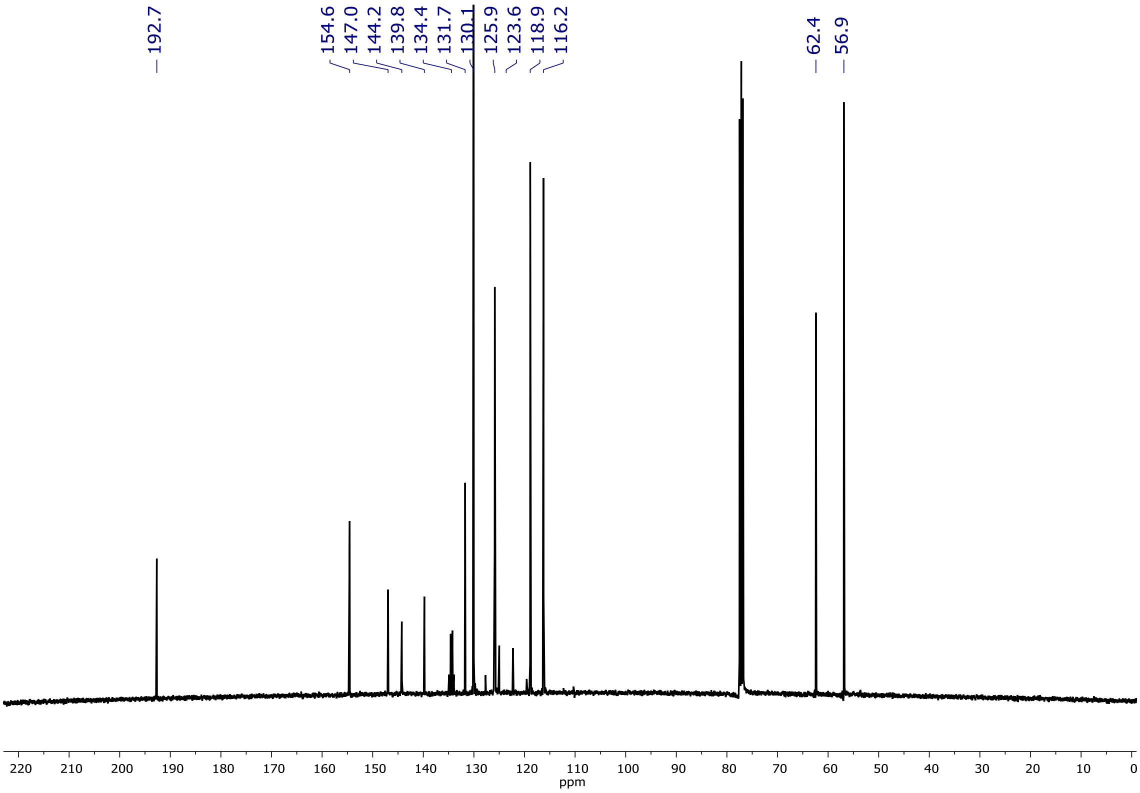

^{13}C NMR (100 MHz, $CDCl_3$)

Figure S13. ^{1}H and ^{13}C NMR spectra of compound **4a**.

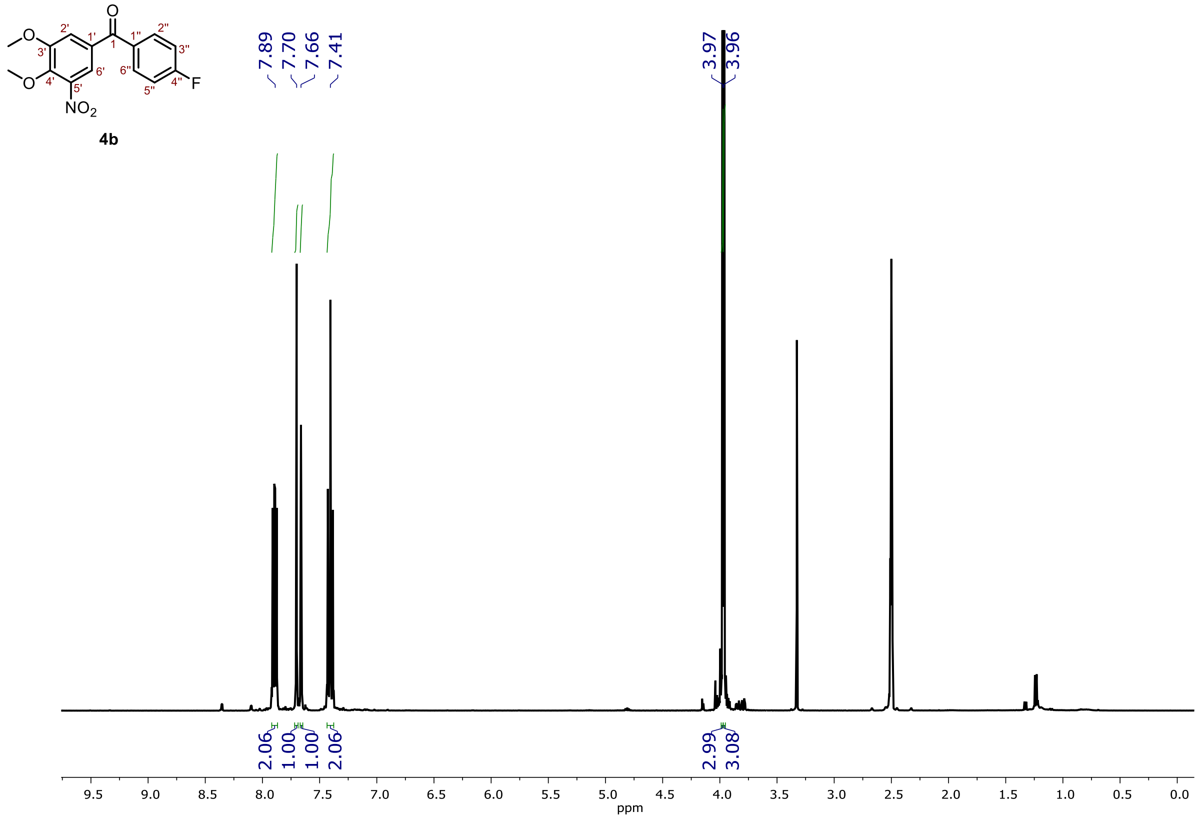

^{1}H NMR (400 MHz, DMSO-d_6)

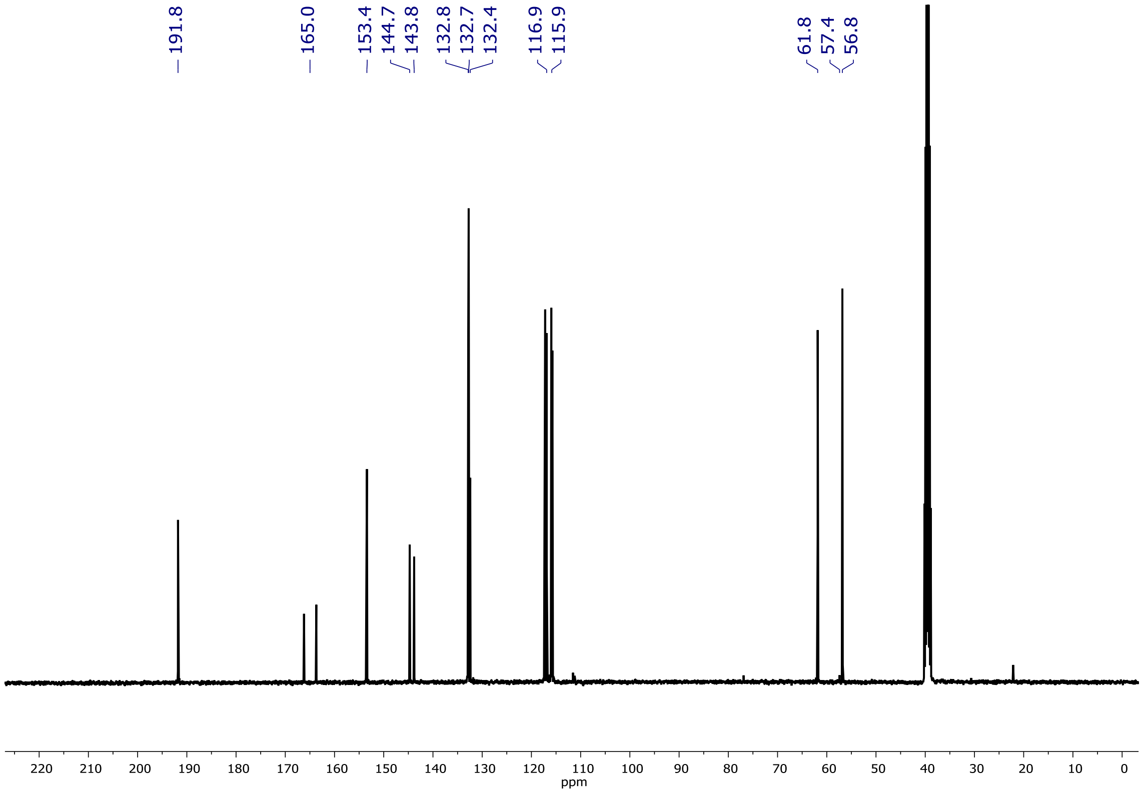

^{13}C NMR (100 MHz, DMSO-d_6)

Figure S14. ^{1}H and ^{13}C NMR spectra of compound **4b**.

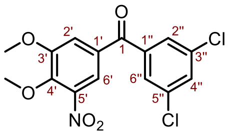

4c

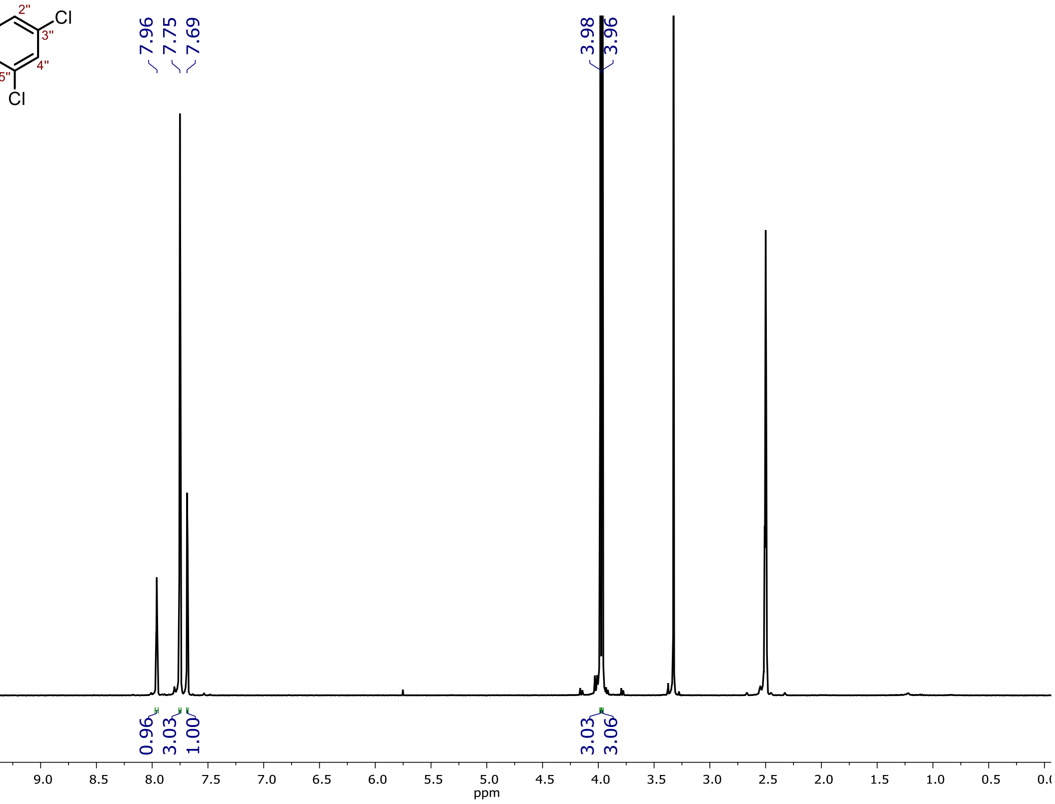

^{1}H NMR (400 MHz, DMSO-d$_6$)

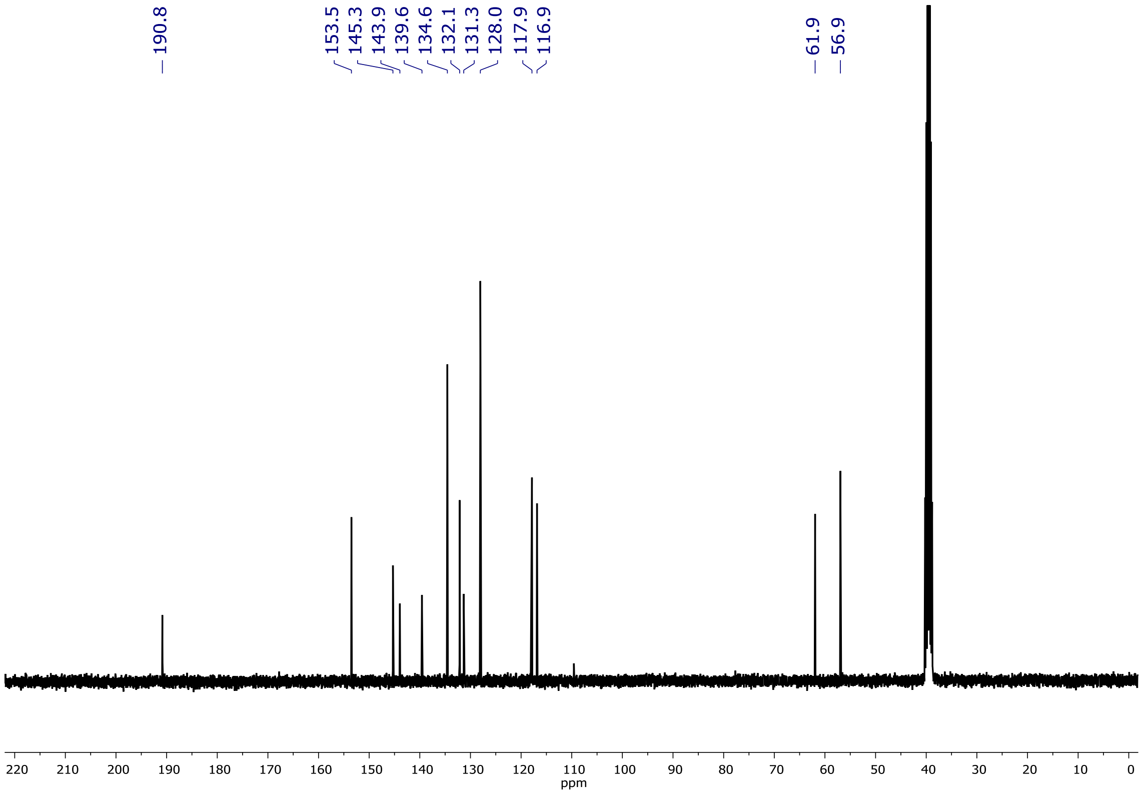

^{13}C NMR (100 MHz, DMSO-d$_6$)

Figure S15. ^{1}H and ^{13}C NMR spectra of compound **4c**.

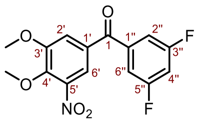

4d

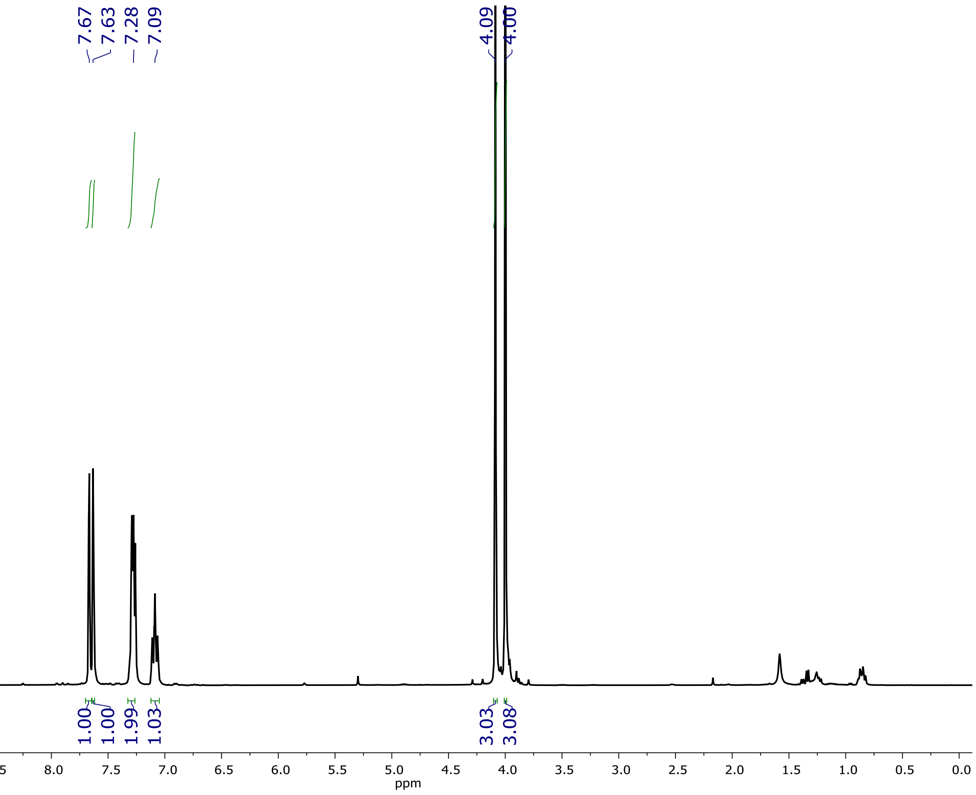

^{1}H NMR (360 MHz, $CDCl_3$)

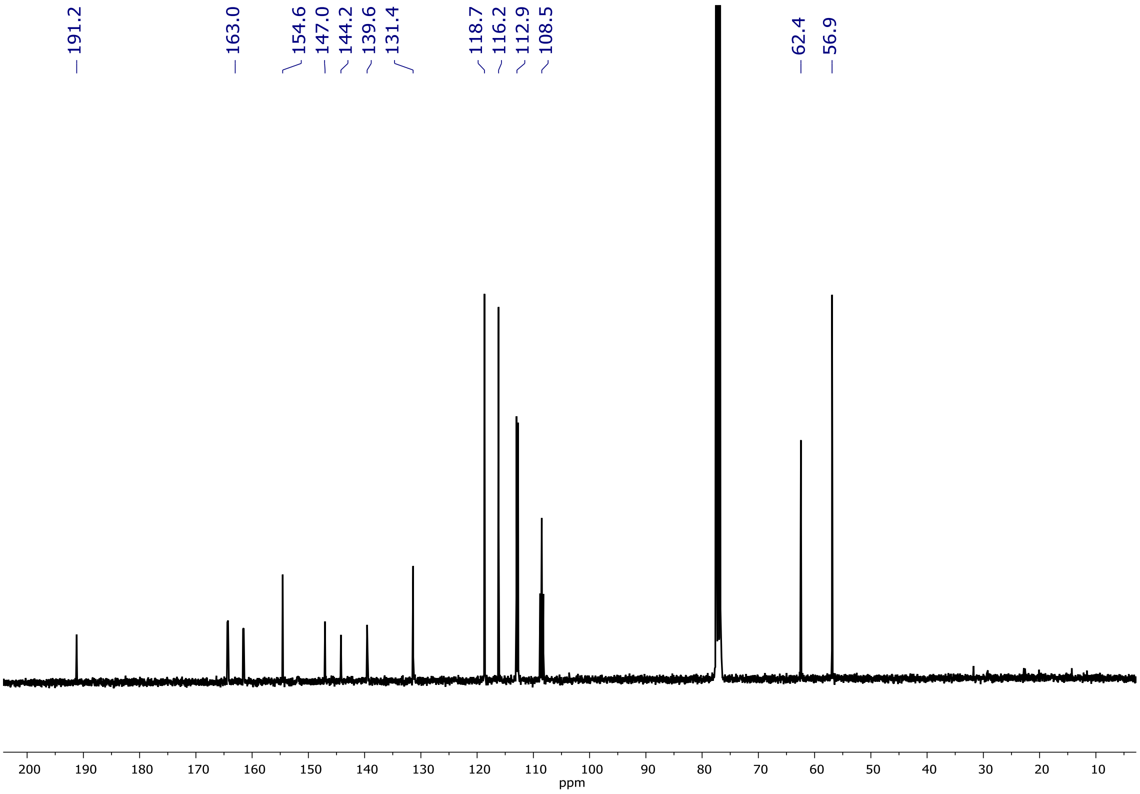

^{13}C NMR (90 MHz, $CDCl_3$)

Figure S16. ^{1}H and ^{13}C NMR spectra of compound **4d**.

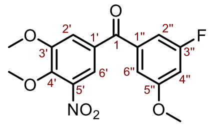

4e

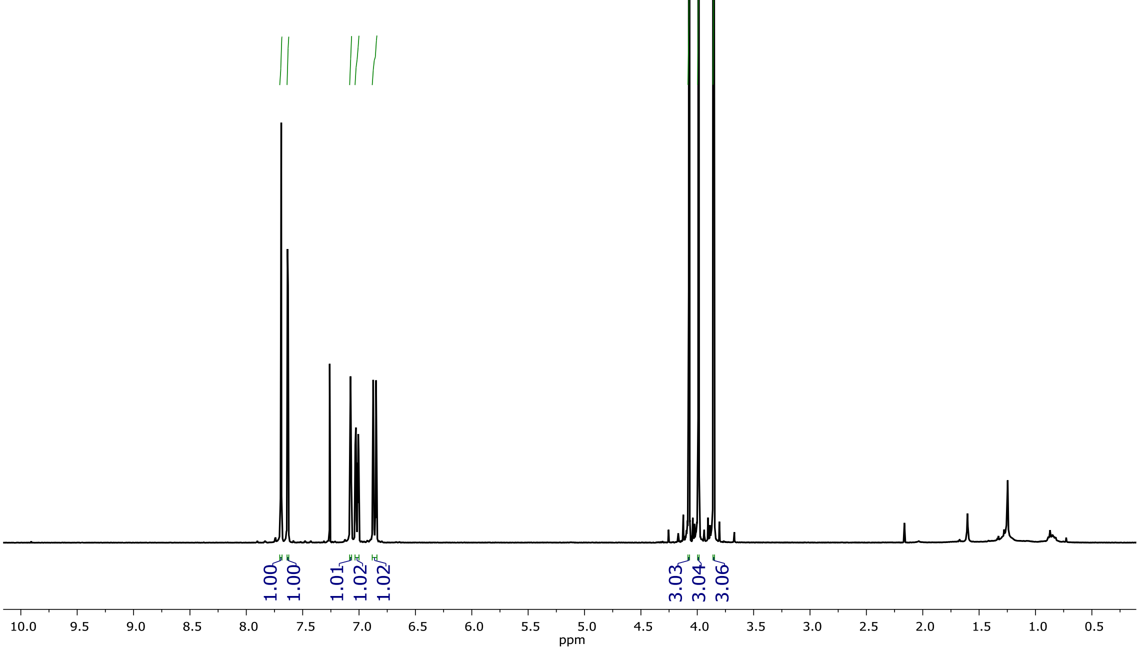

^{1}H NMR (400 MHz, $CDCl_3$)

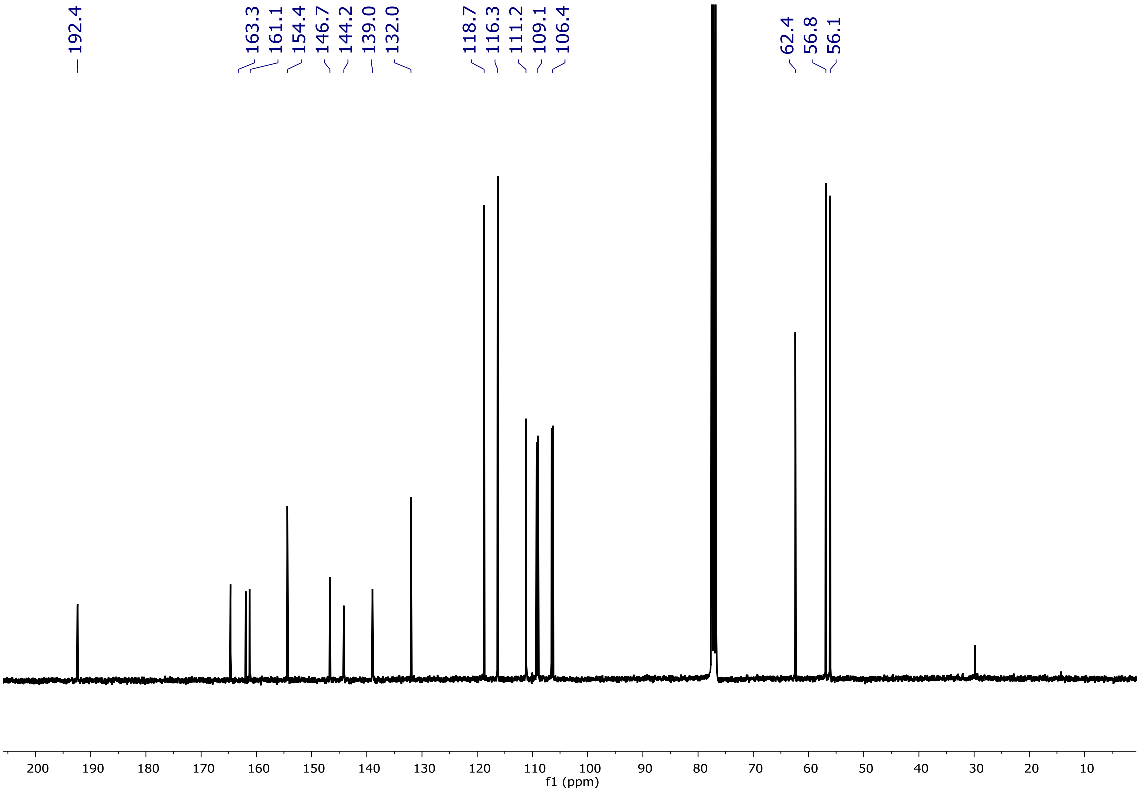

^{13}C NMR (90 MHz, $CDCl_3$)

Figure S17. ^{1}H and ^{13}C NMR spectra of compound **4e**.

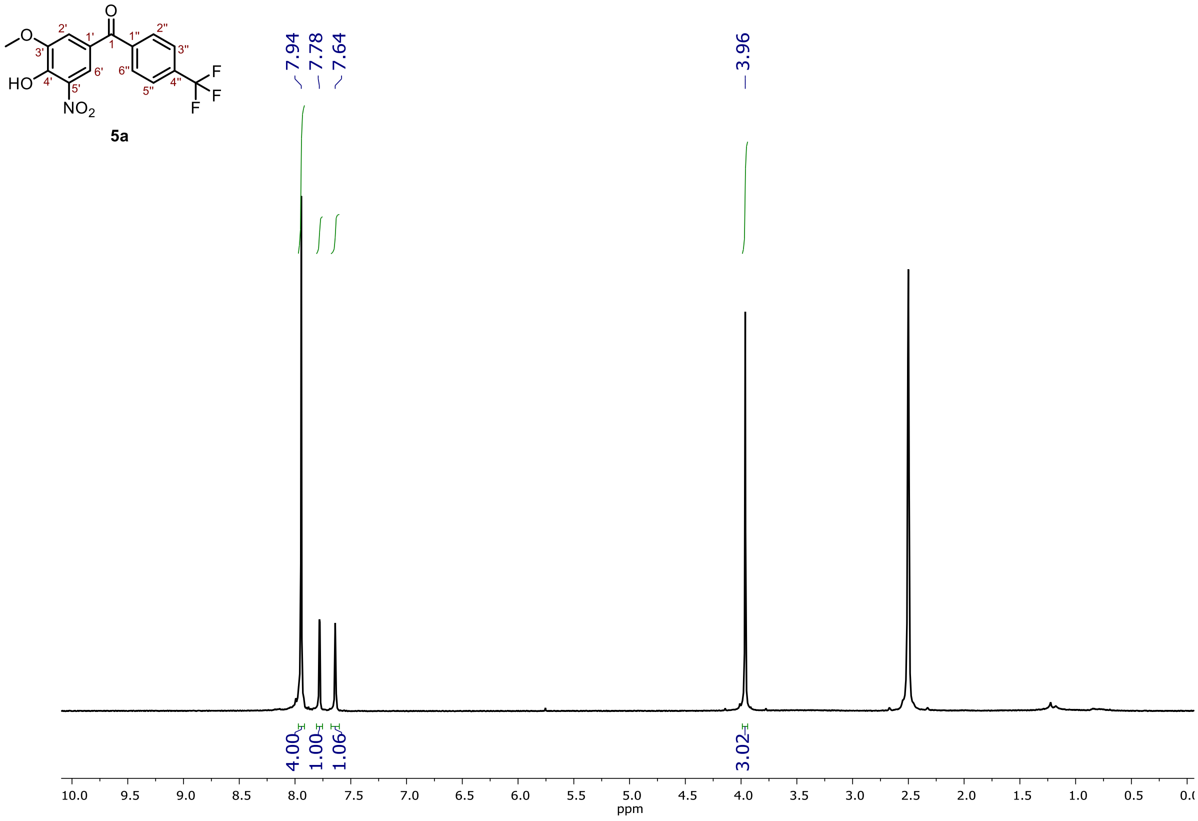

^{1}H NMR (400 MHz, DMSO-d_6)

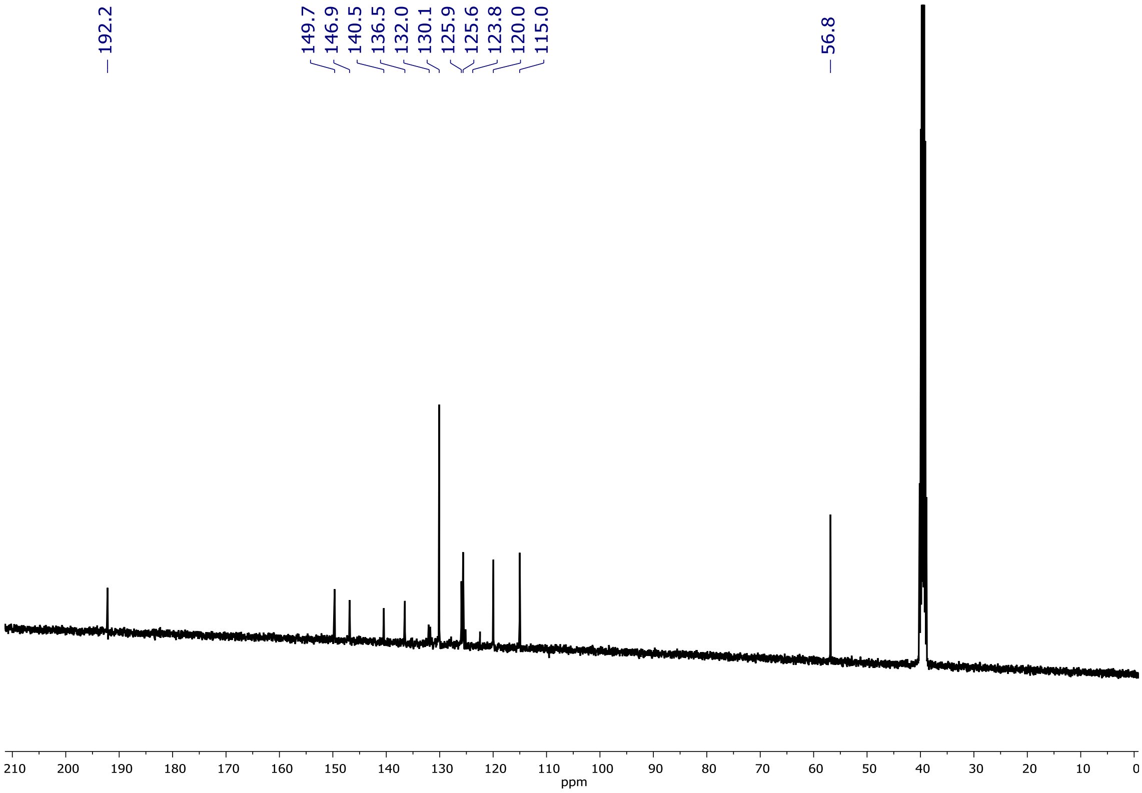

^{13}C NMR (100 MHz, DMSO-d_6)

Figure S18. ^{1}H and ^{13}C NMR and spectra of compound **5a**.

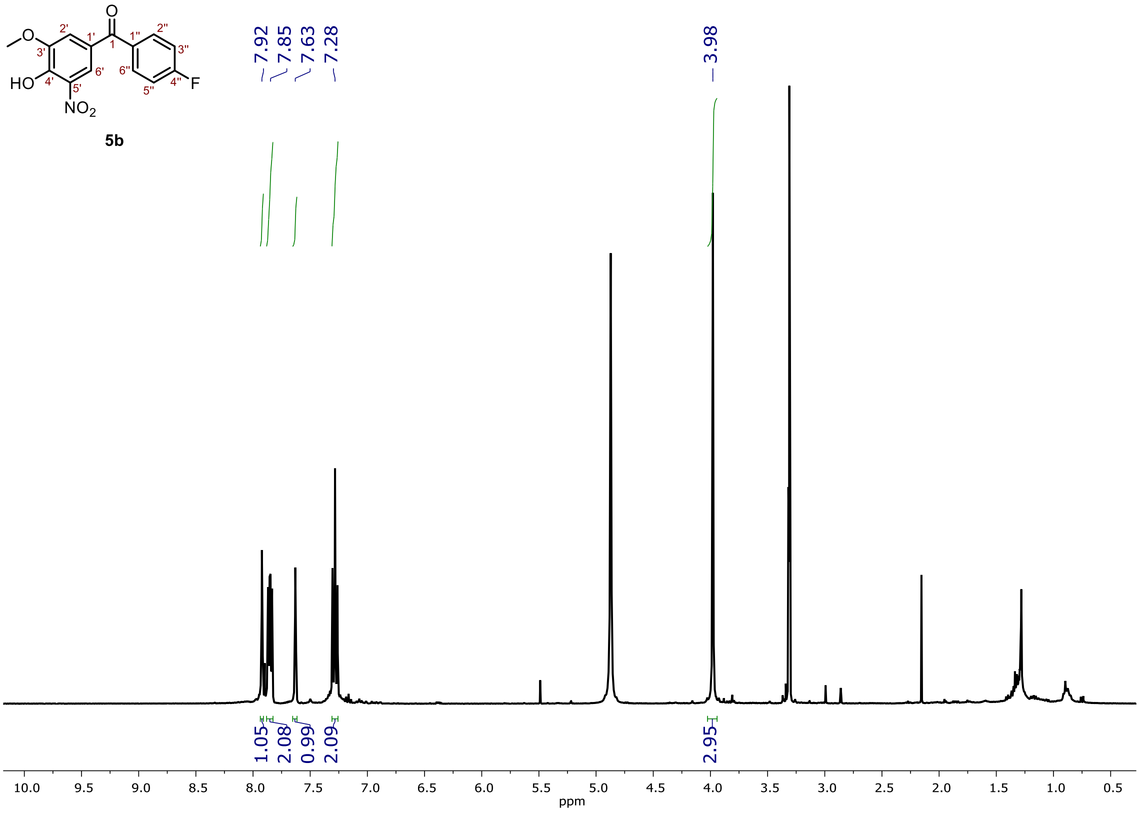

^{1}H NMR (400 MHz, methanol-d_4)

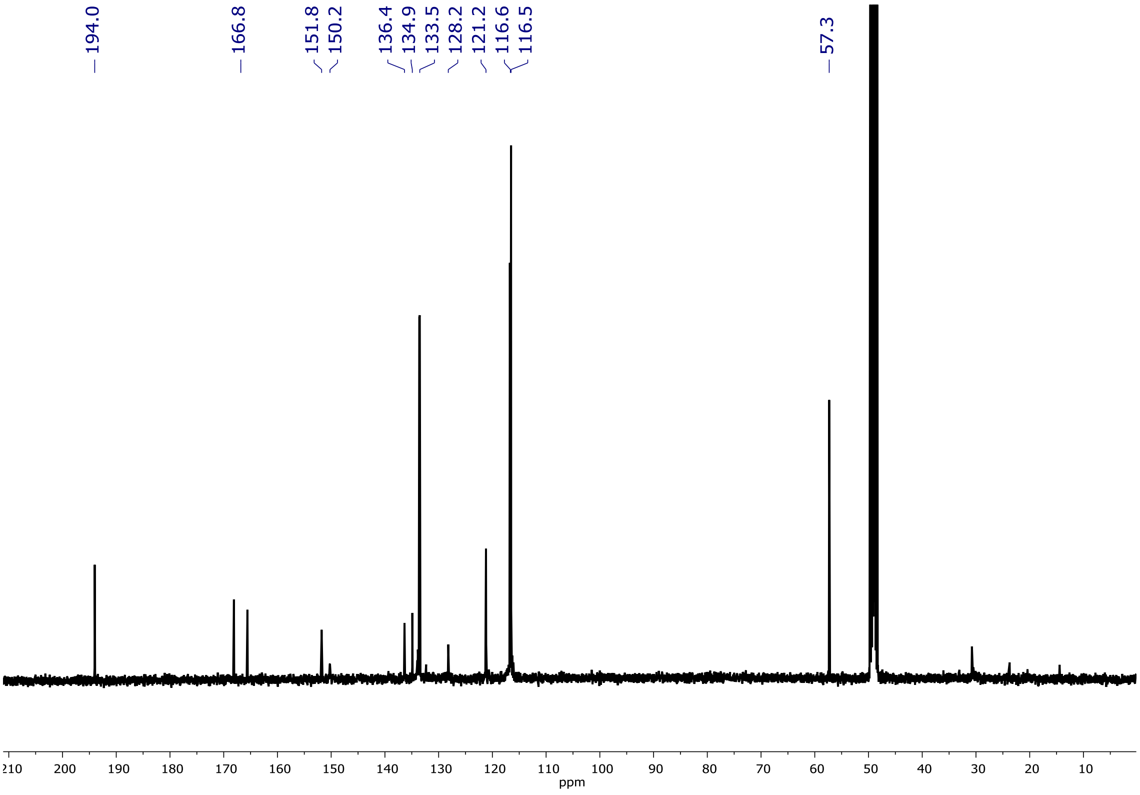

^{13}C NMR (100 MHz, methanol-d_4)

Figure S19. ^{1}H and ^{13}C NMR spectra of compound **5b**.

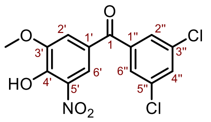

5c

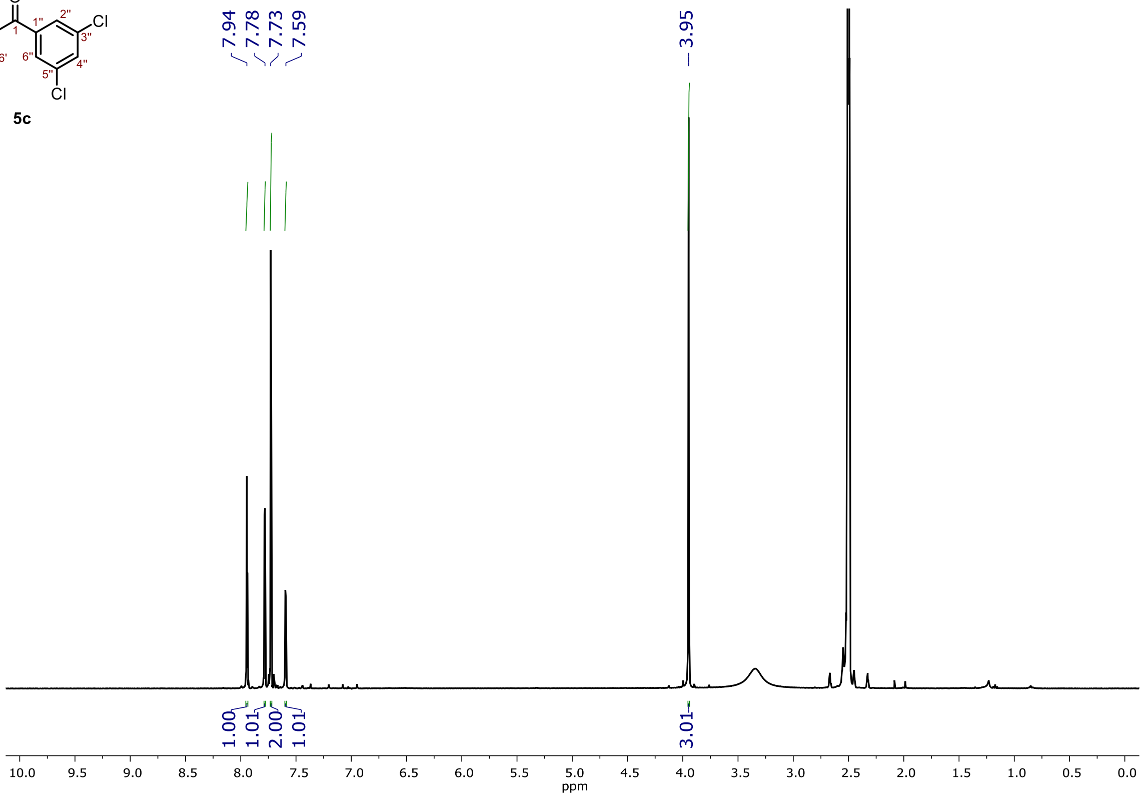

¹H NMR (400 MHz, DMSO-d6)

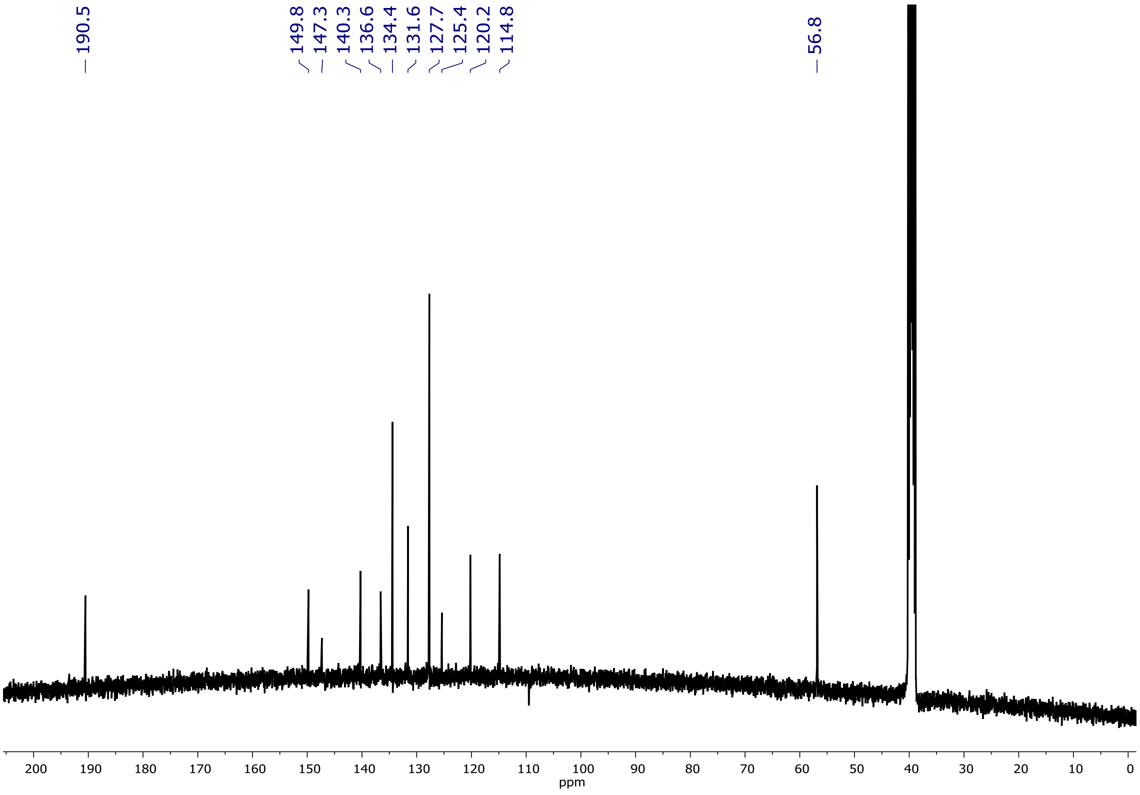

¹³C NMR (100 MHz, DMSO-d6)

Figure S20. ¹H and ¹³C NMR spectra of compound **5c**.

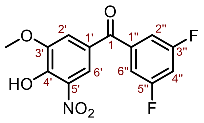

5d

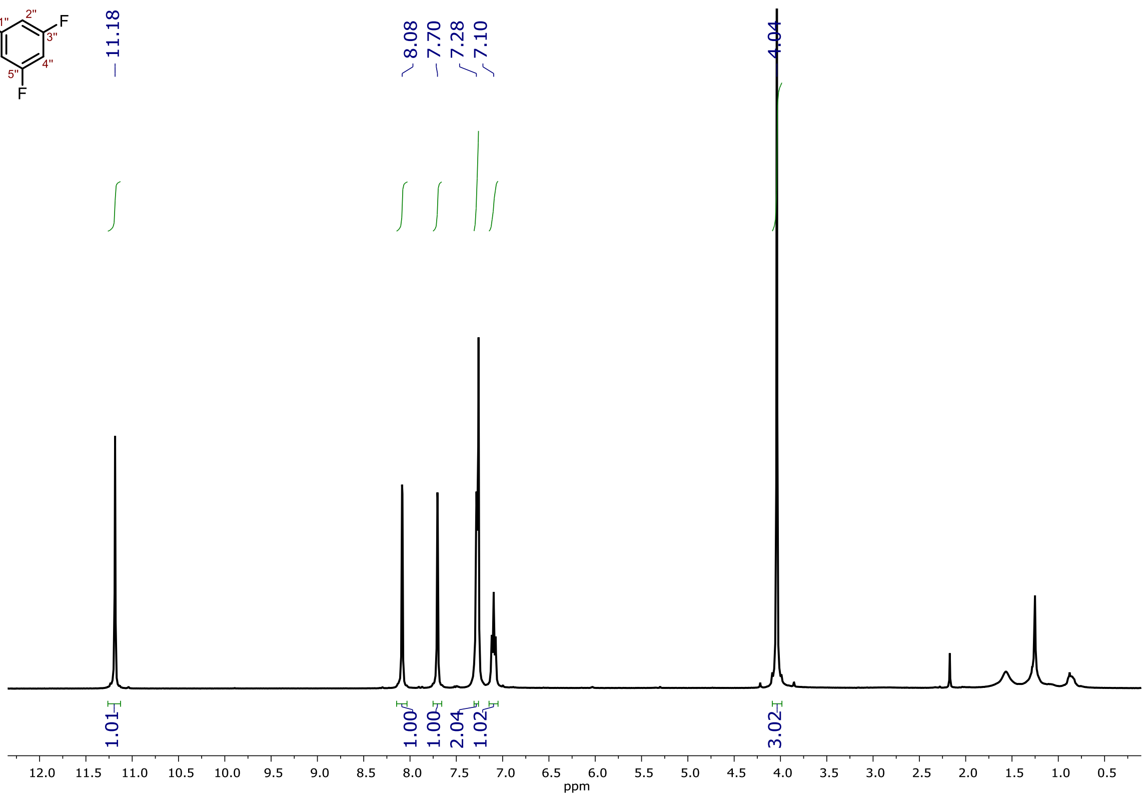

^{1}H NMR (400 MHz, $CDCl_3$)

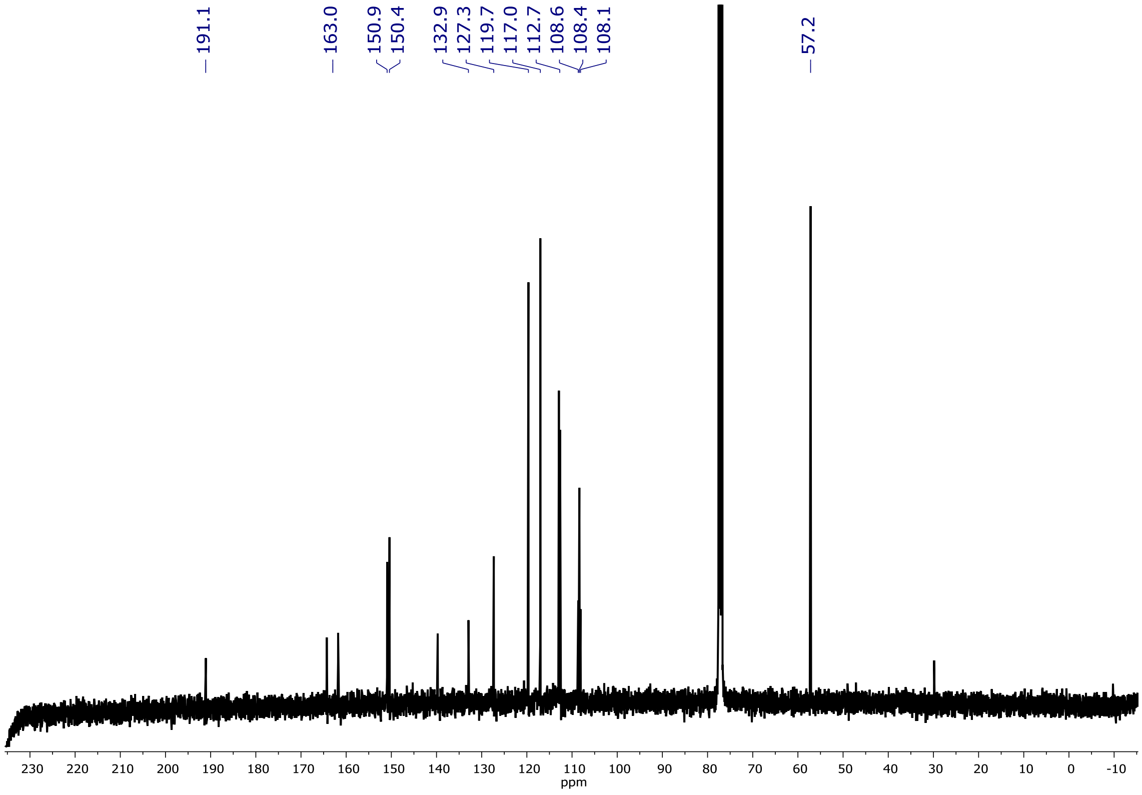

^{13}C NMR (100 MHz, $CDCl_3$)

Figure S21. ^{1}H and ^{13}C NMR spectra of compound **5d**.

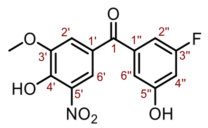

5e

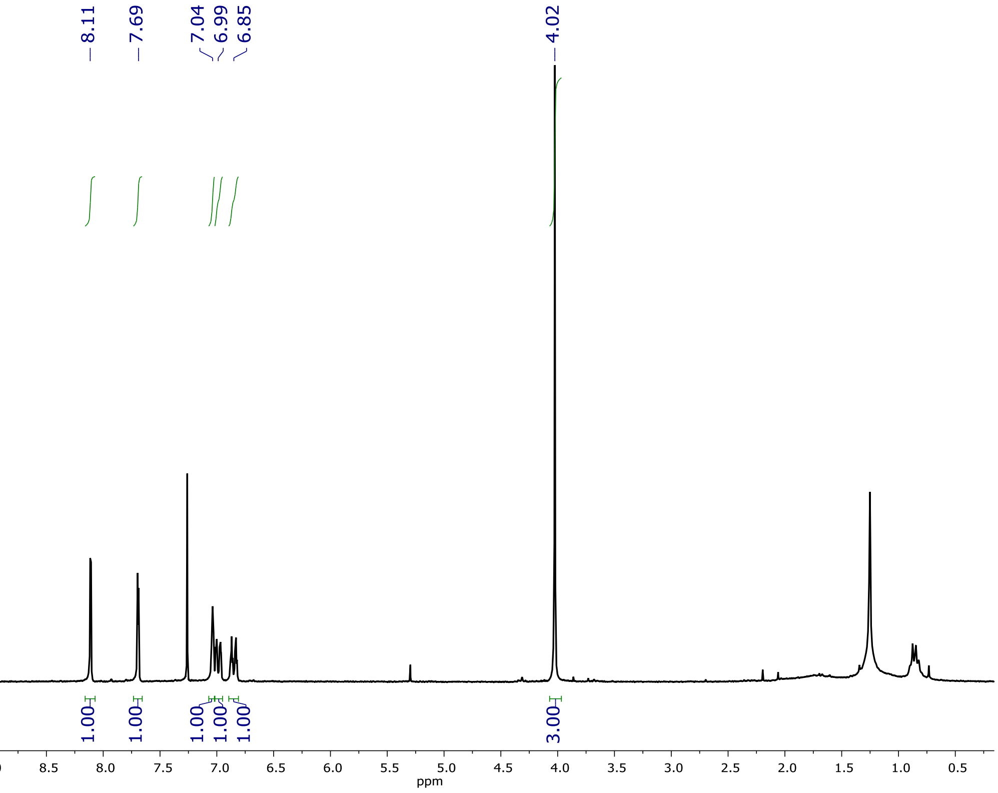

^{1}H NMR (250 MHz, $CDCl_3$)

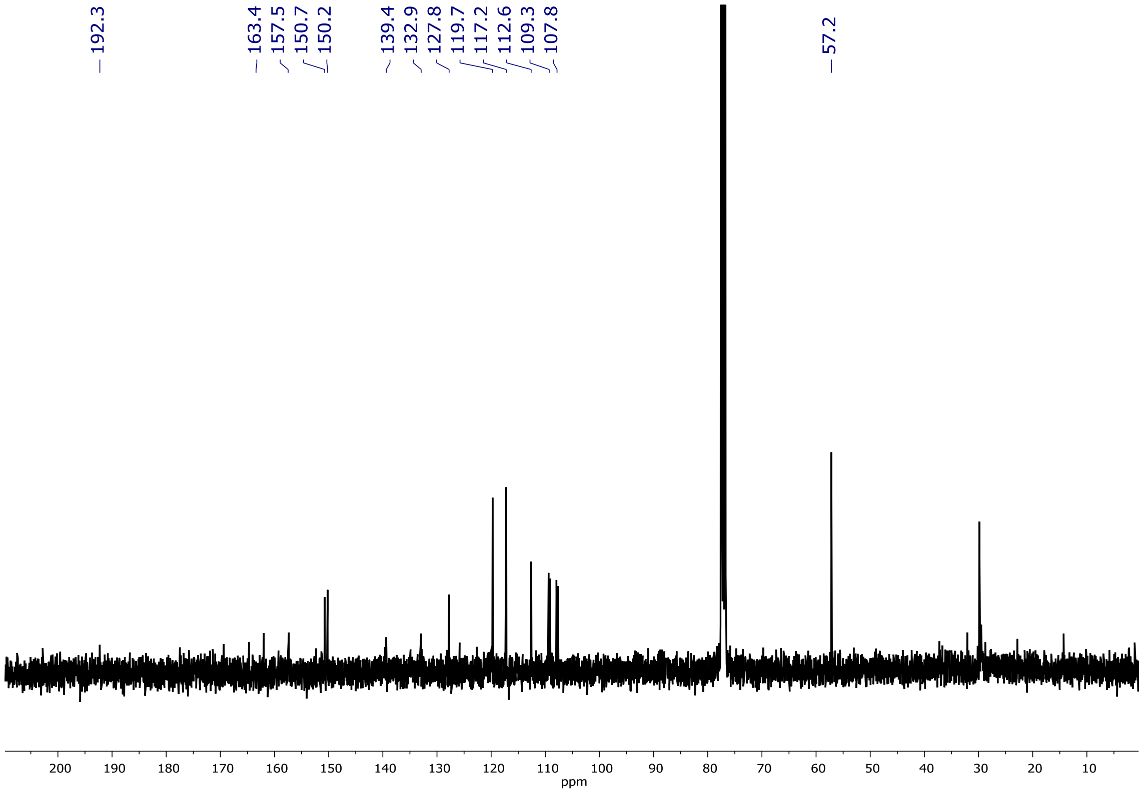

^{13}C NMR (90 MHz, $CDCl_3$)

Figure S22. ^{1}H and ^{13}C NMR spectra of compound **5e**.

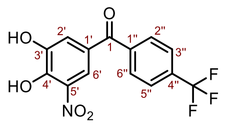

M-14

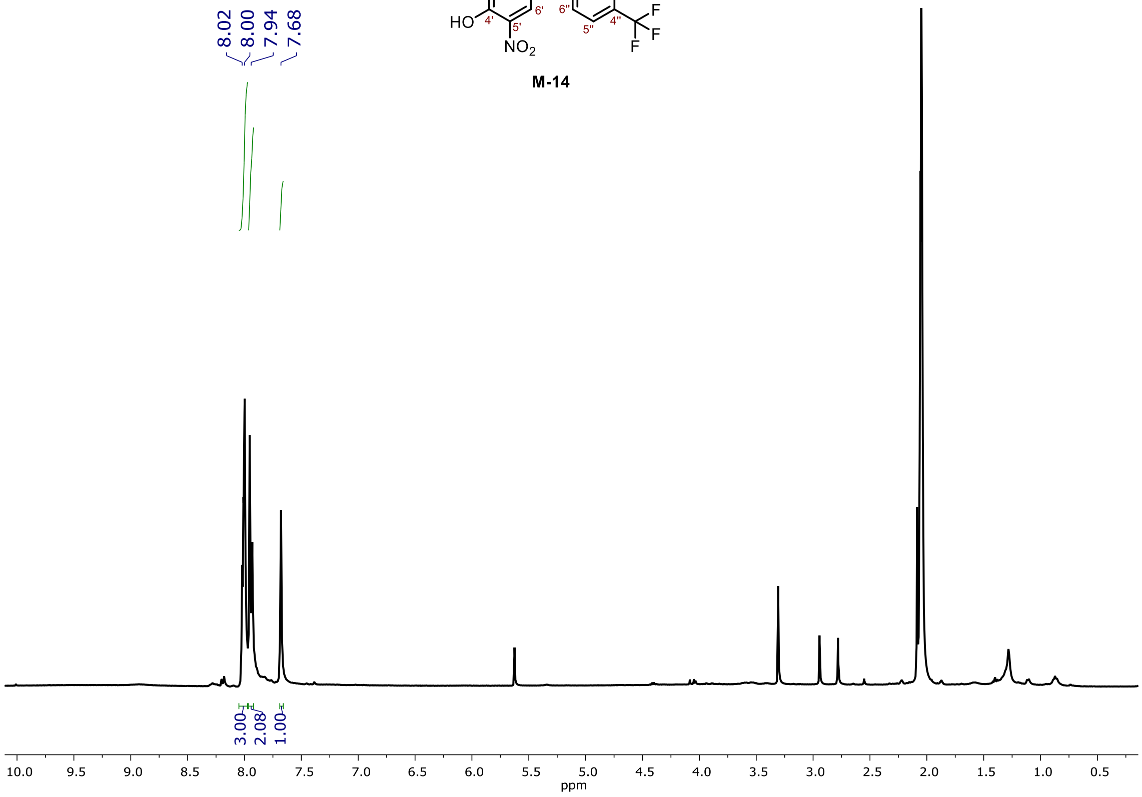

^{1}H NMR (360 MHz, acetone-d$_6$)

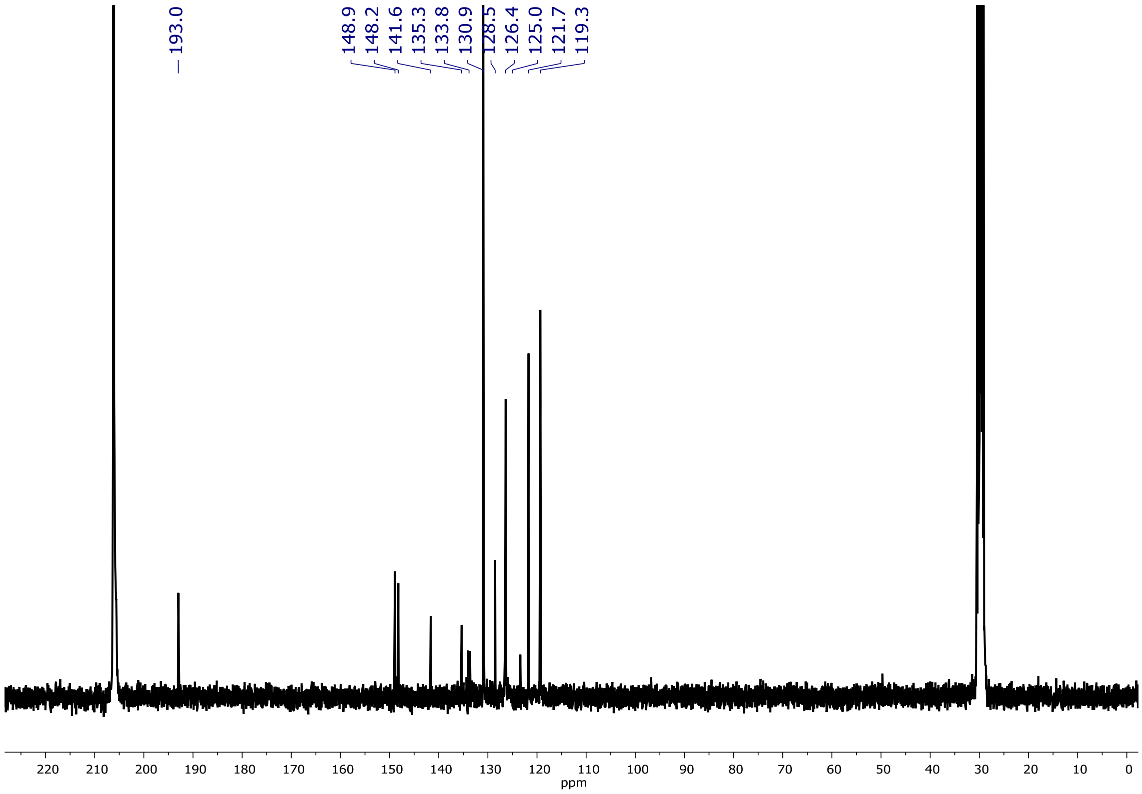

^{13}C NMR (90 MHz, acetone-d$_6$)

Figure S23. ^{1}H and ^{13}C NMR spectra of compound **M-14**.

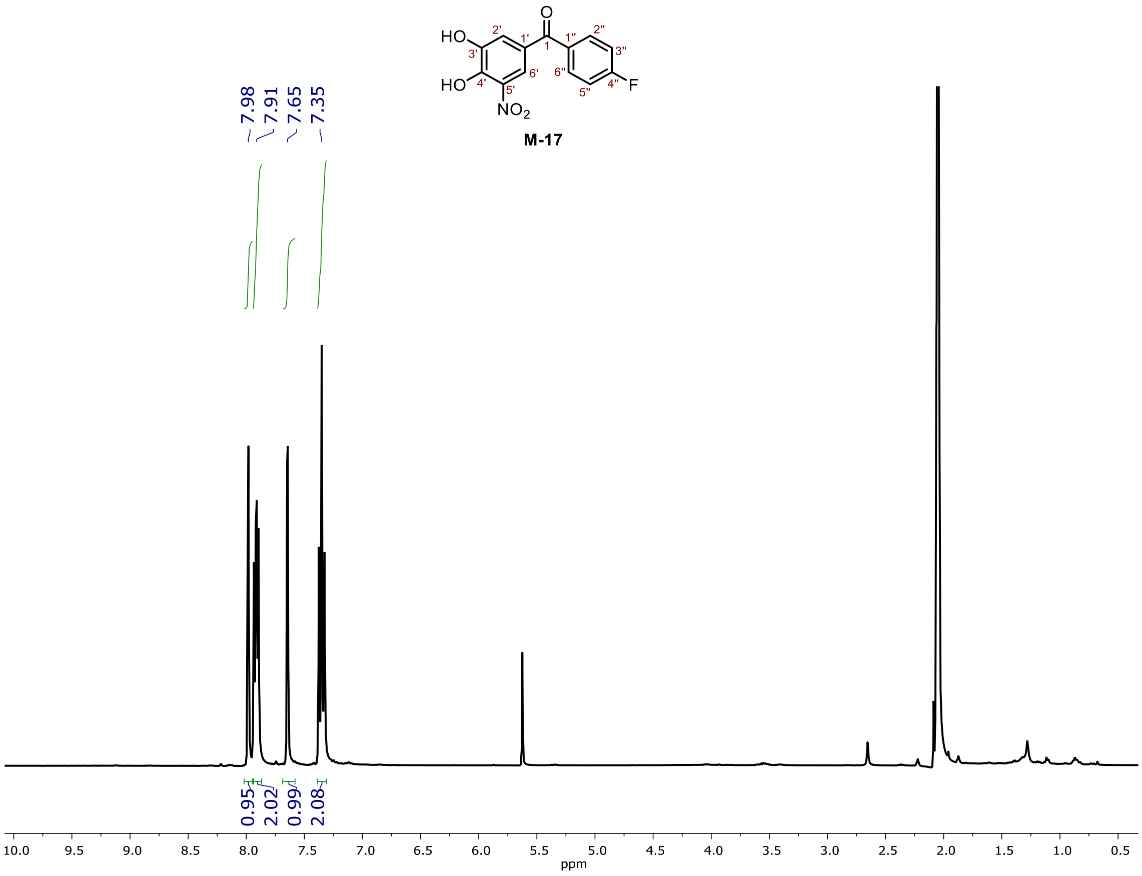

^{1}H NMR (360 MHz, acetone-d$_6$)

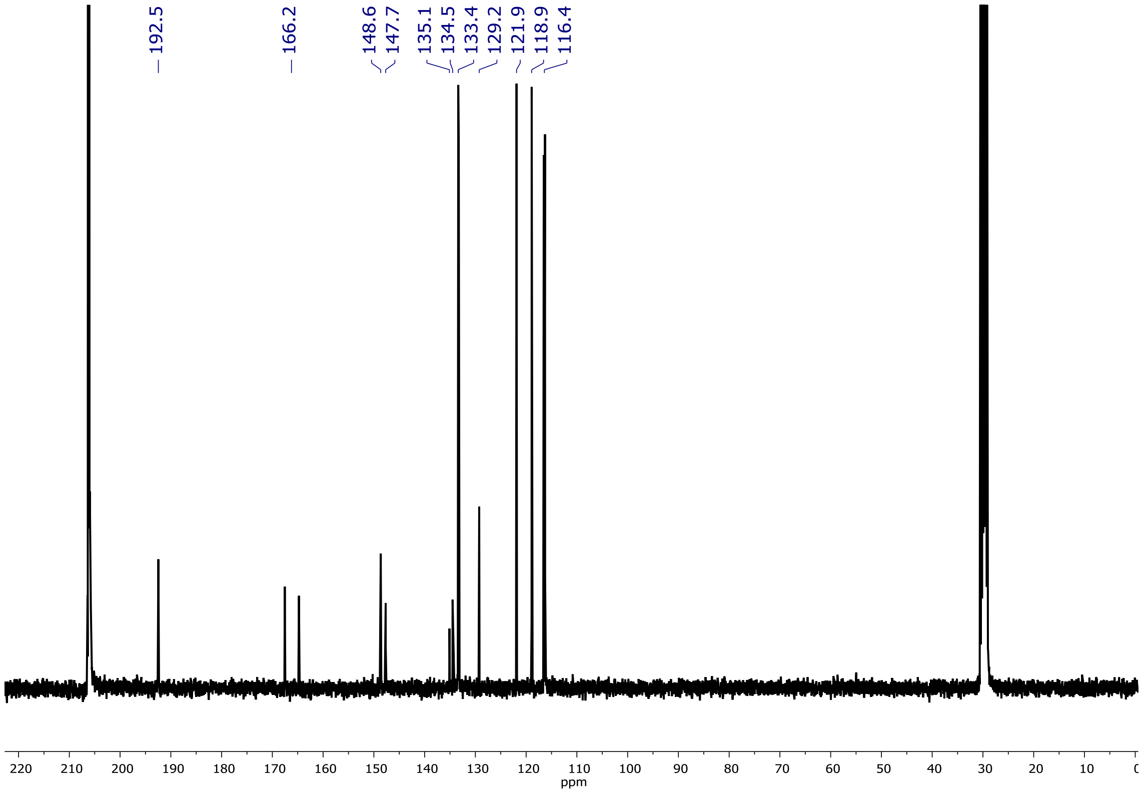

^{13}C NMR (90 MHz, acetone-d$_6$)

Figure S24. ^{1}H and ^{13}C NMR spectra of compound **M-17**.

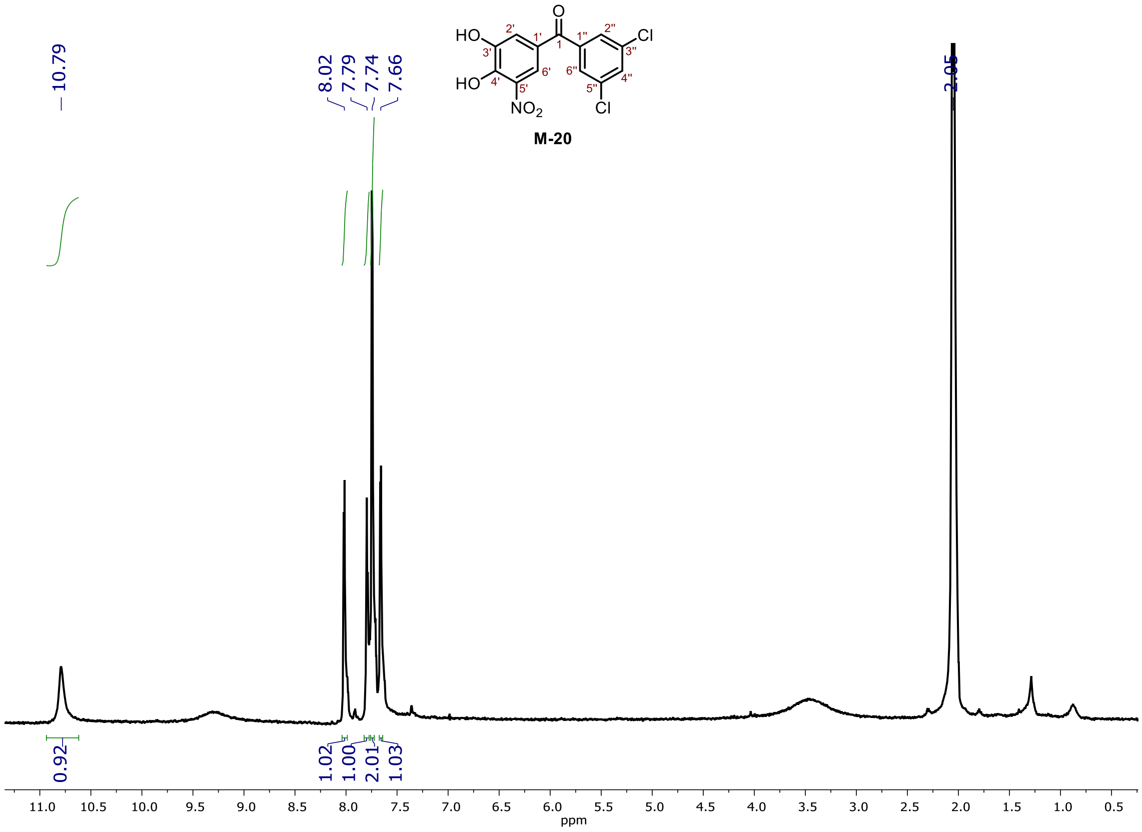

¹H NMR (250 MHz, acetone-d6)

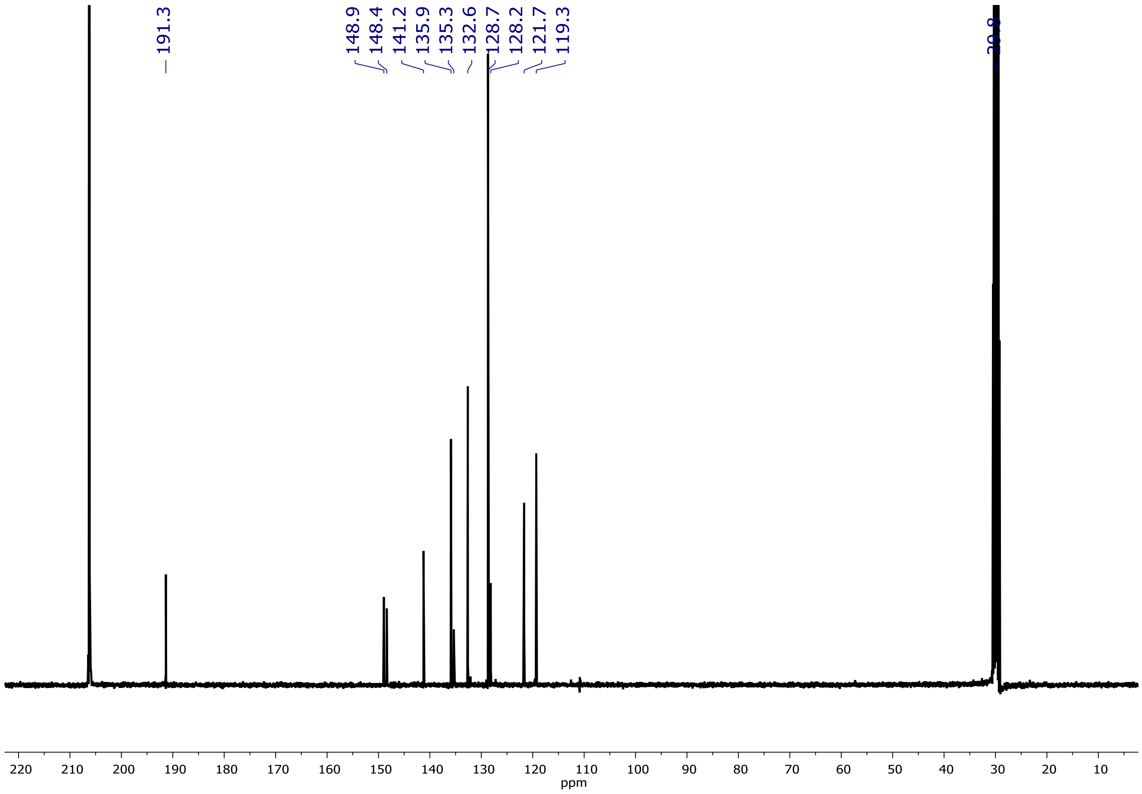

¹³C NMR (100 MHz, acetone-d6)

Figure S25. ¹H and ¹³C NMR spectra of compound **M-20**.

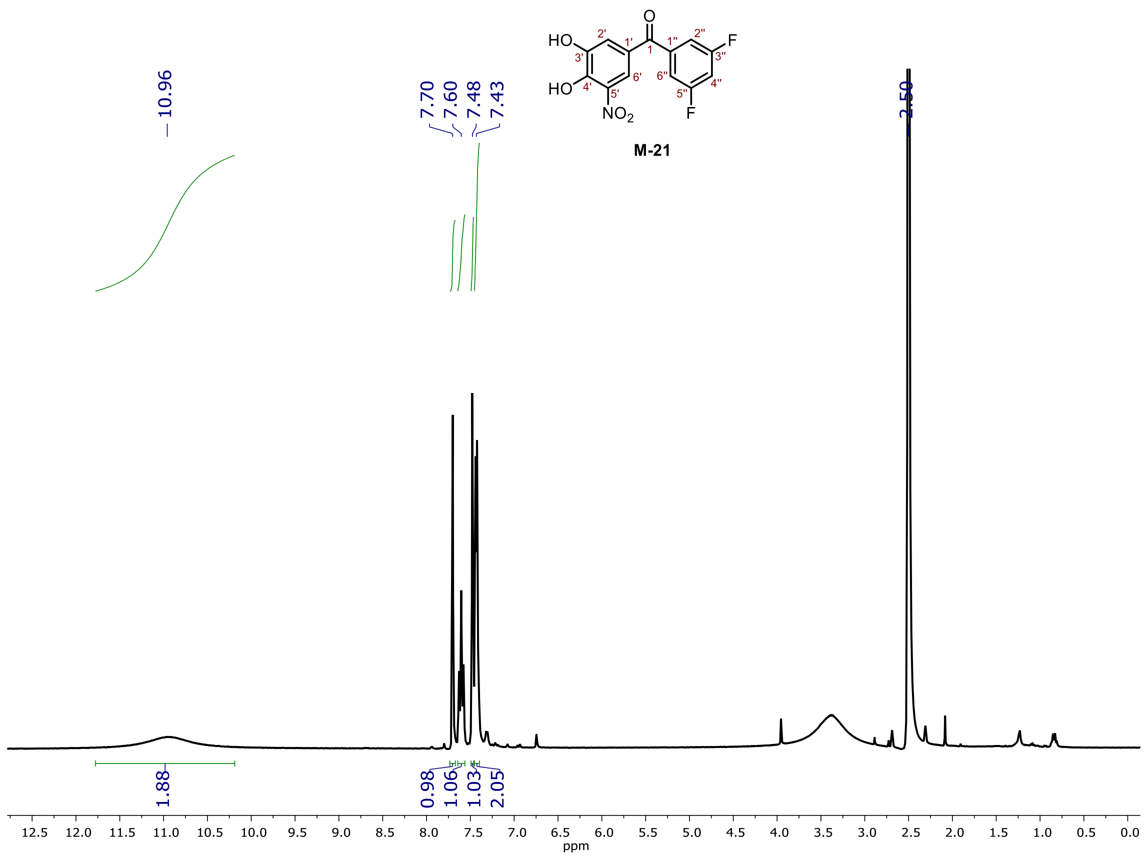

^{1}**H NMR** (360 MHz, DMSO-d$_6$)

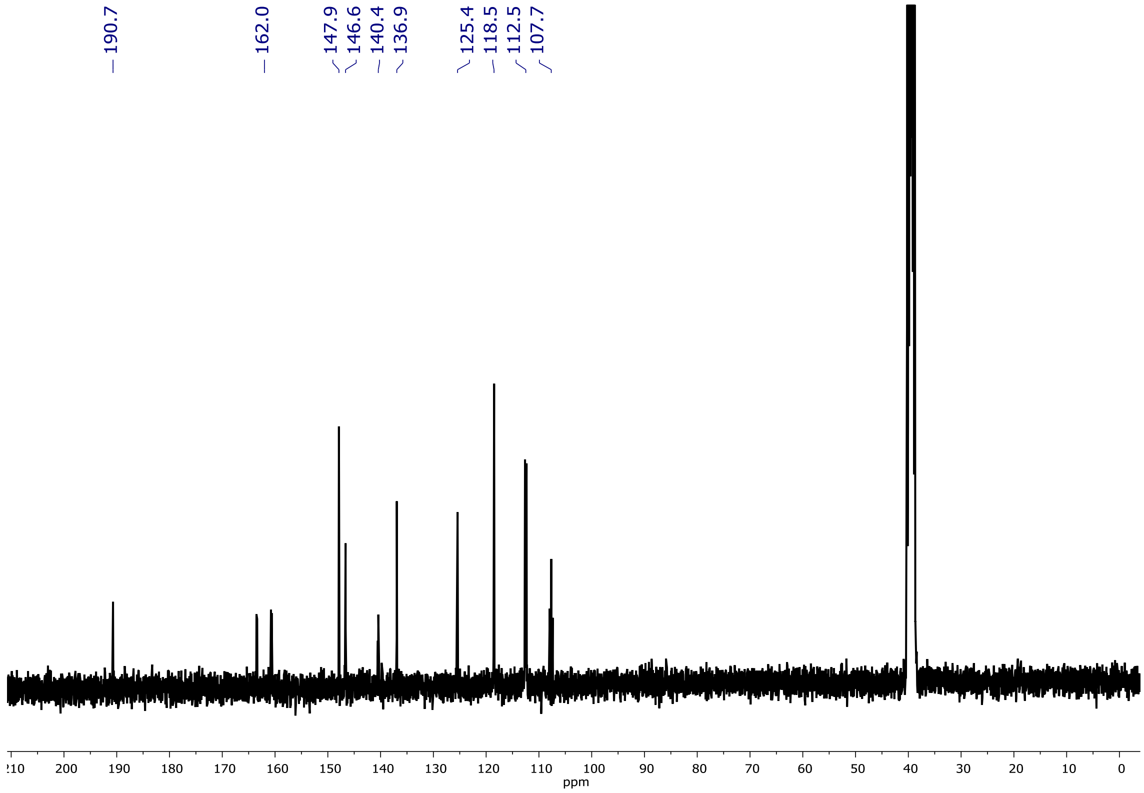

^{13}**C NMR** (90 MHz, DMSO-d$_6$)

Figure S26. ^{1}H and ^{13}C NMR spectra of compound **M-21**.

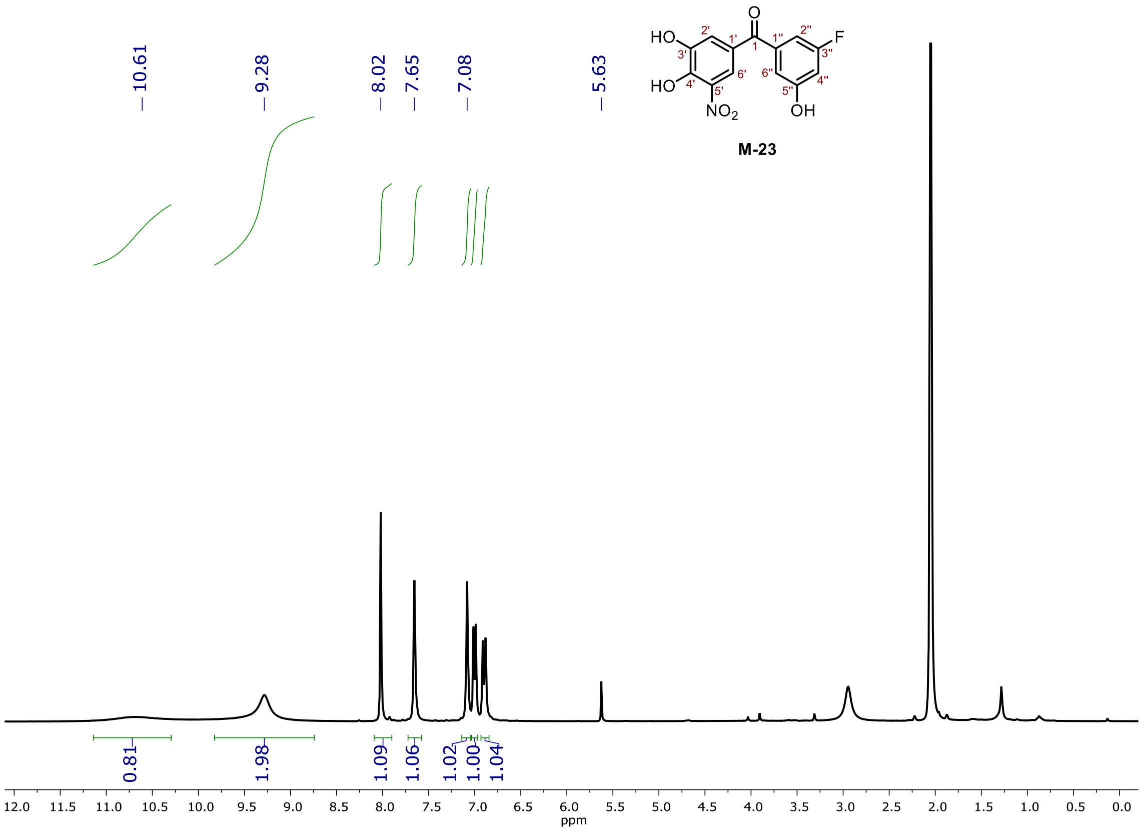

^{1}**H NMR** (360 MHz, acetone-d$_6$)

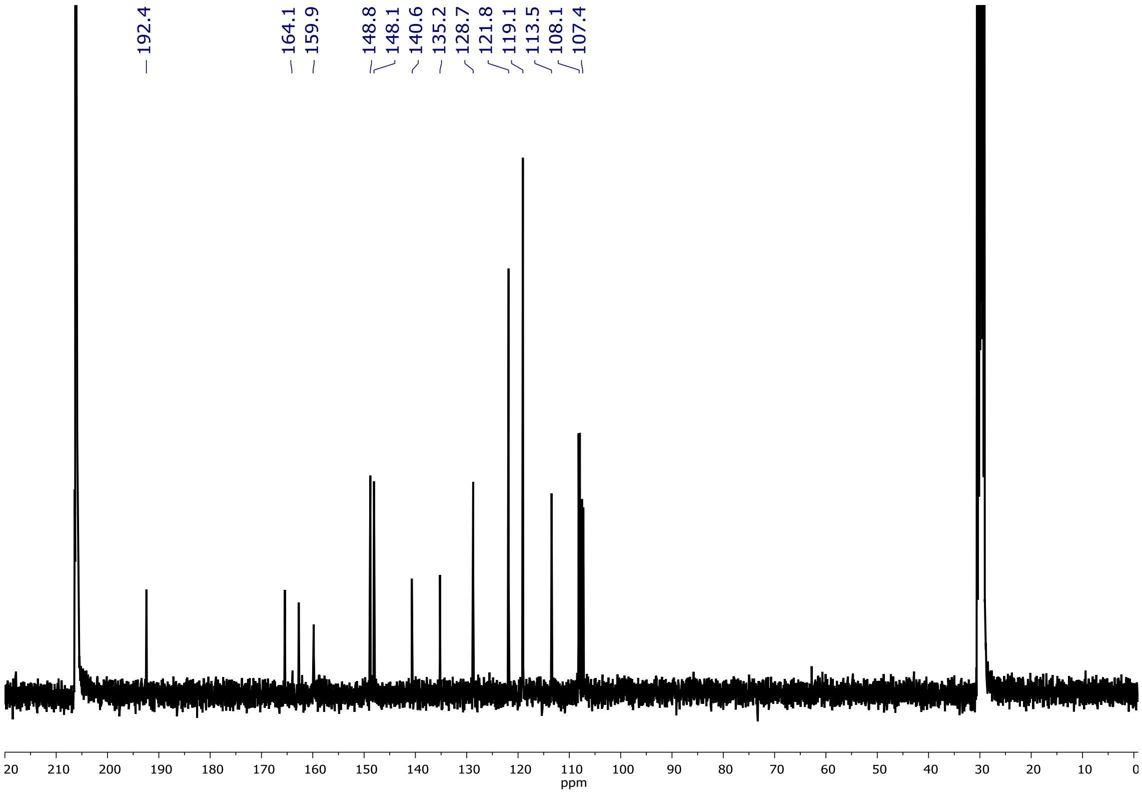

^{13}**C NMR** (90 MHz, acetone-d$_6$)

Figure S27. ^{1}H and ^{13}C NMR spectra of compound **M-23**.

HPLC traces for the final compounds

HPLC analysis of all final compounds was performed on Agilent-1200 HPLC with a binary pump and photodiode array detector (DAD), using a Phenomenex Luna Omega C18 column (150 mm × 4.6 mm, 5 µm). Gradient elution at a flow rate of 1 mL min^{-1} was performed by mixing 0.1% HCOOH in Water / 0.1% HCOOH in CAN.

(3,4-Dihydroxy-5-nitrophenyl)-[4-(trifluoromethyl)phenyl]methanone, **M-14**

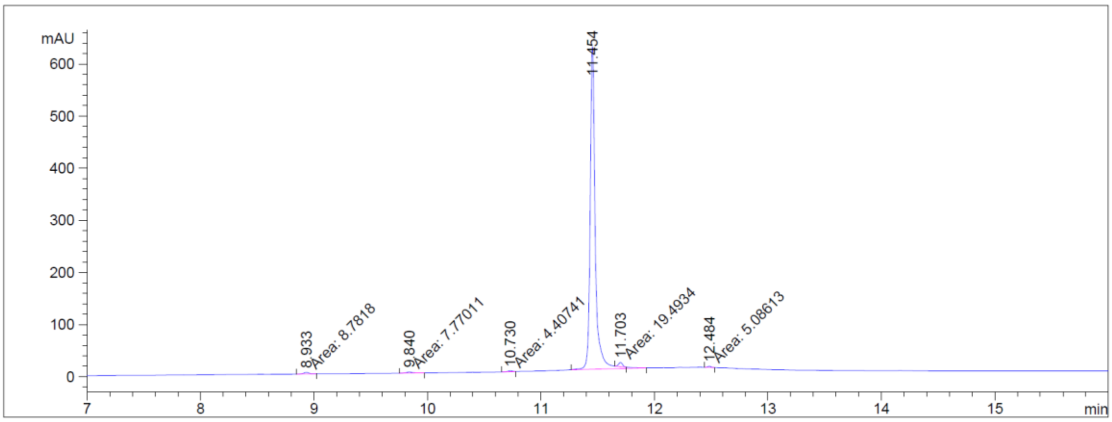

Area Percent Report

Signal 1: DAD1 C, Sig=254,4 Ref=850,100

Peak #	RetTime [min]	Type	Width [min]	Area [mAU*s]	Height [mAU]	Area %
1	8.933	MM	0.0503	8.78180	2.90987	0.4427
2	9.840	MM	0.0607	7.77011	2.13294	0.3917
3	10.730	MM	0.0456	4.40741	1.61013	0.2222
4	11.454	MM R	0.0521	1938.04407	620.13800	97.7042
5	11.703	MM T	0.0398	19.49343	8.17263	0.9827
6	12.484	MM	0.0362	5.08613	2.34038	0.2564
Totals :				1983.58294	637.30395	

*** End of Report ***

Figure S28. HPLC analysis for **M-14.**

(3,4-Dihydroxy-5-nitrophenyl)-(4-fluorophenyl)methanone, **M-17**

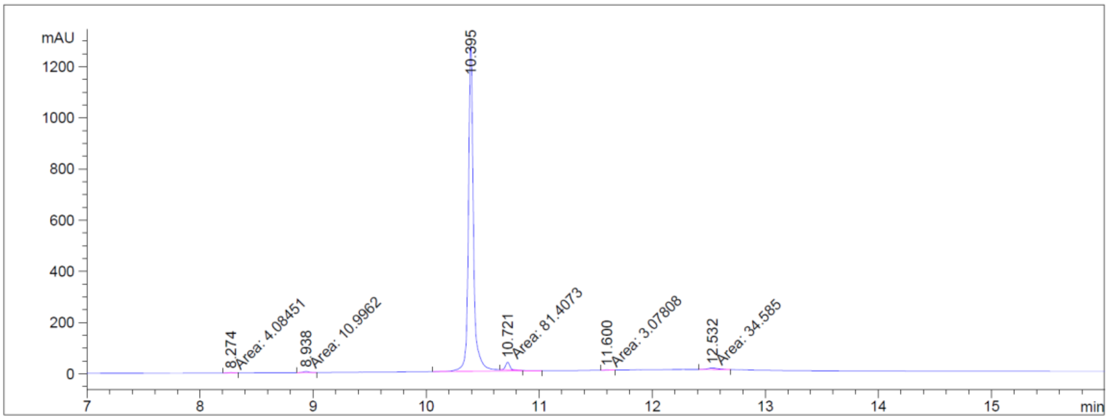

Area Percent Report

Signal 1: DAD1 C, Sig=254,4 Ref=850,100

Peak #	RetTime [min]	Type	Width [min]	Area [mAU*s]	Height [mAU]	Area %
1	8.274	MM	0.0507	4.08451	1.34252	0.0983
2	8.938	MM	0.0518	10.99619	3.53849	0.2647
3	10.395	MM R	0.0527	4020.18896	1272.45691	96.7708
4	10.721	MM T	0.0453	81.40733	29.96630	1.9596
5	11.600	MM	0.0498	3.07808	1.02924	0.0741
6	12.532	MM	0.1162	34.58496	4.96011	0.8325
Totals :				4154.34004	1313.29357	

*** End of Report ***

Figure S29. HPLC analysis for **M-17**.

(3,5-Dichlorophenyl)-(3,4-dihydroxy-5-nitrophenyl)methanone, **M-20**

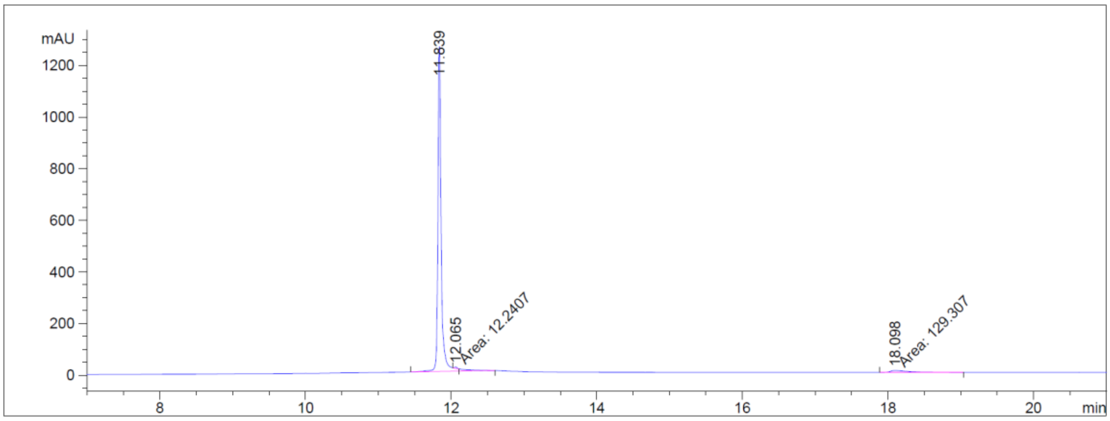


```
=====================================================================
                     Area Percent Report
=====================================================================

Signal 1: DAD1 C, Sig=254,4 Ref=850,100

Peak RetTime Type  Width     Area      Height     Area
  #   [min]        [min]   [mAU*s]     [mAU]       %
----|-------|----|-------|----------|----------|--------|
   1  11.839 MM R  0.0533 4037.51025 1261.70142  96.6129
   2  12.065 MM T  0.0373   12.24067    5.46862   0.2929
   3  18.098 MM    0.3013  129.30652    7.15321   3.0942

Totals :                  4179.05744 1274.32324

=====================================================================
                      *** End of Report ***
```

Figure S30. HPLC analysis for **M-20**.

(3,5-Difluorophenyl)-(3,4-dihydroxy-5-nitrophenyl)methanone, **M-21**

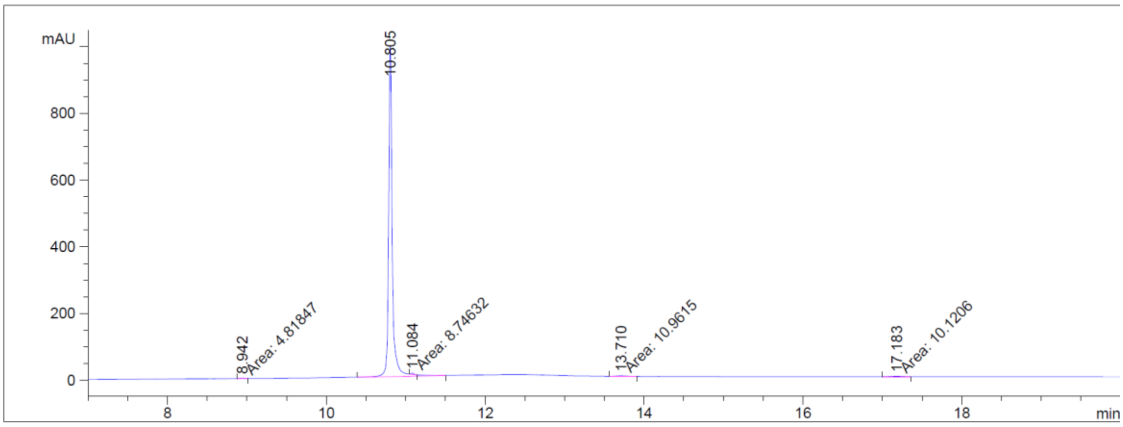

Area Percent Report

Signal 1: DAD1 C, Sig=254,4 Ref=850,100

Peak #	RetTime [min]	Type	Width [min]	Area [mAU*s]	Height [mAU]	Area %
1	8.942	MM	0.0508	4.81847	1.58075	0.1492
2	10.805	MM R	0.0538	3194.64185	989.05627	98.9271
3	11.084	MM T	0.0399	8.74632	3.65545	0.2708
4	13.710	MM	0.1271	10.96149	1.43683	0.3394
5	17.183	MM	0.1415	10.12057	1.19178	0.3134
Totals :				3229.28871	996.92109	

*** End of Report ***

Figure S31. HPLC analysis for **M-21**.

(3,4-dihydroxy-5-nitrophenyl)-(3-fluoro-5-hydroxyphenyl)methanone, **M-23**

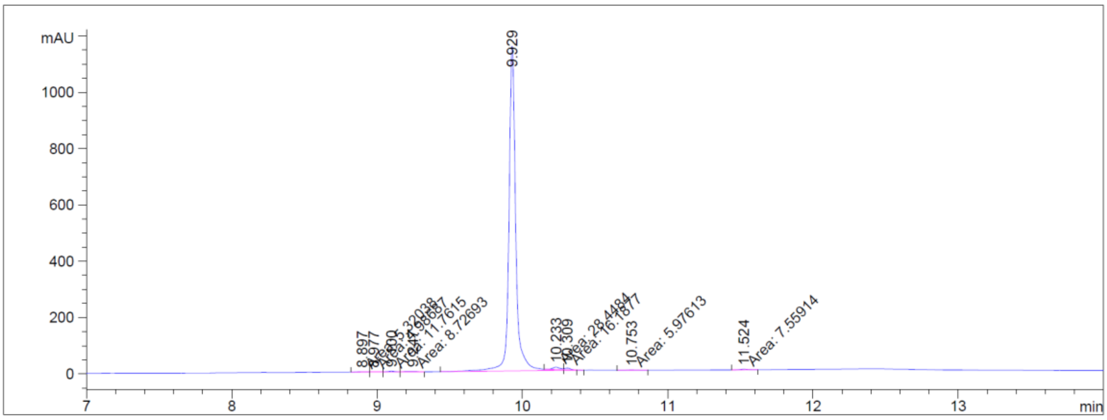

```
=====================================================================
                         Area Percent Report
=====================================================================

Signal 1: DAD1 C, Sig=254,4 Ref=850,100

Peak RetTime Type  Width     Area      Height     Area
  #   [min]        [min]   [mAU*s]     [mAU]        %
----|-------|----|-------|----------|----------|--------|
   1   8.897 MF    0.0803    5.32038    1.10473   0.1368
   2   8.977 FM    0.0770    4.98687    1.07999   0.1282
   3   9.100 FM    0.0652   11.76150    3.00604   0.3024
   4   9.247 FM    0.0938    8.72693    1.55105   0.2244
   5   9.929 MM R  0.0548 3799.80054 1154.64966  97.7122
   6  10.233 MF T  0.0685   28.44840    8.40367   0.7316
   7  10.309 FM T  0.0450   16.18774    5.99999   0.4163
   8  10.753 MM    0.0806    5.97613    1.23617   0.1537
   9  11.524 MM    0.0619    7.55914    2.03585   0.1944

Totals :                  3888.76762 1179.06714

=====================================================================
                          *** End of Report ***
```

Figure S32. HPLC analysis for **M-23**.

References

1. Sant'Anna, R.; Gallego, P.; Robinson, L. Z.; Pereira-Henriques, A.; Ferreira, N.; Pinheiro, F.; Esperante, S.; Pallares, I.; Huertas, O.; Almeida, M. R.; Reixach, N.; Insa, R.; Velazquez-Campoy, A.; Reverter, D.; Reig, N.; Ventura, S. Repositioning tolcapone as a potent inhibitor of transthyretin amyloidogenesis and associated cellular toxicity. *Nat. Commun.* **2016**, 7, 10787.
